# Ligand‐Mediated Regioselective Rhodium‐Catalyzed Benzotriazole–Allene Coupling: Mechanistic Exploration and Quantum Chemical Analysis

**DOI:** 10.1002/chem.201905359

**Published:** 2020-02-04

**Authors:** Tetiana Sergeieva, Trevor A. Hamlin, Sergiy Okovytyy, Bernhard Breit, F. Matthias Bickelhaupt

**Affiliations:** ^1^ Institute for Organic Chemistry University of Freiburg Albertstrasse 21 79104 Freiburg im Breisgau Germany; ^2^ Department of Theoretical Chemistry Amsterdam Center for Multiscale Modeling (ACMM) Vrije Universiteit Amsterdam De Boelelaan 1083 1081 HV Amsterdam The Netherlands; ^3^ Department of Chemistry Oles Honchar Dnipro National University 72 Gagarina Avn. 49000 Dnipro Ukraine; ^4^ Institute for Molecules and Materials (IMM) Radboud University Heyendaalseweg 135 6525 AJ Nijmegen The Netherlands

**Keywords:** activation strain analysis, allylation, DPEphos ligands, N−H activation, rhodium

## Abstract

The ligand‐controlled rhodium‐catalyzed regioselective coupling of 1,2,3‐benzotriazoles and allenes was investigated by DFT calculations. Because allylation can occur at either the N1 or N2 position of the 1,2,3‐benzotriazole, the complete Gibbs free energy profiles for both pathways were computed. A kinetic preference emerged for the experimentally observed N1 allylation with the JoSPOphos ligand, whereas N2 allylation was favored with DPEphos. Analysis of the regiodetermining oxidative addition step by using the activation strain model in conjunction with a matching energy decomposition analysis has revealed that the unprecedented N2 reaction regioselectivity is dictated by the strength of the electrostatic interactions between the 1,2,3‐benzotriazole and the rhodium catalyst. The nature of the electrostatic interaction was rationalized by analysis of the electrostatic potential maps and Hirshfeld charges: a stabilizing electrostatic interaction was found between the key atoms involved in the oxidative addition for the N2 pathway, analogous interactions are weaker in the N1 case.

## Graphical abstract

**The reaction mechanism** of the ligand‐controlled regioselective 1,2,3‐benzotriazoles‐allenes coupling catalyzed by rhodium was analyzed computationally. The mechanistic investigation revealed a kinetic preference for the unusual N2 pathway over the alternative N1 when the DPEphos ligand is employed. Our activation strain and bonding analyses on the regiodetermining oxidative addition step identified the stabilizing electrostatic interaction to be responsible for the reaction regioselectivity (see scheme).

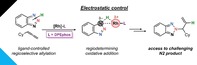

Controlling the regioselectivity of fundamental organic transformations is an outstanding challenge in synthesis.^1^ The N‐selective functionalization of 1,2,3‐benzotriazoles is a particular challenge due to the dynamic equilibrium between two tautomeric forms of the benzotriazole (Scheme 1 a).^2^ Despite of the greater stability of tautomer **1 a**, alkylation of 1,2,3‐benzotriazole has typically been observed to be moderately selective at N1/N3 position and extremely unselective at N2.

**Scheme 1:**
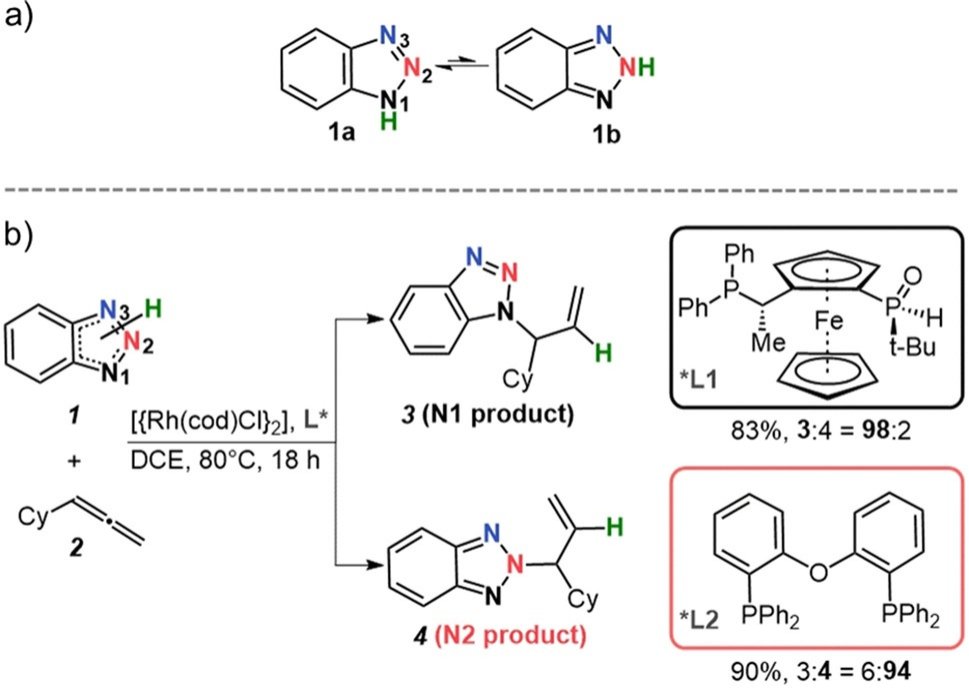
(a) Tautomeric equilibrium of 1,2,3‐benzotriazole; (b) Synthetic protocol of regioselective Rh catalyzed addition of 1,2,3‐benzotriazoles to allenes.

Major advances have been forged for N1‐selective methods.^3^ N2‐selective modification is underdeveloped, primarily because of difficulties in shifting the equilibrium toward **1 b** tautomer. As a consequence, the structural space around valuable N2‐substituted 1,2,3‐benzotriazoles that often occur in pharmaceutical agents, agrochemicals and advanced materials is limited.^3^, ^4^

The rare examples of N2 procedures reported during the last decade mostly rely on structural modification of the coupling partners.^3^ These methods are usually based on sterically bulky groups on the benzotriazole or alkylating agent that block access to the terminal nitrogens N1/N3, thereby making the N2 position the preferred site of nucleophilic attack. Despite being practically successful, these strategies are restricted in functional‐group tolerance and require prefunctionalized reactants. Transition‐metal catalysis offers an alternative approach that circumvents these previously mentioned issues as it involves the direct activation of X−H bonds for functionalizing molecules. In 2014, we proposed an unprecedented rhodium (Rh) catalyzed allylation of 1,2,3‐benzotriazoles. This methodology was attractive in the sense that it gave high N1/N2 regioselectivity and had a high tolerance toward a broad range of 1,2,3‐benzotriazole and allene derivatives.^5^ Importantly, the N1 or N2 regioselectivity could be tuned by judicious choice of the ligand on the Rh center. Among a series of diphosphine ligands used for the initial screening, we found that JoSPOphos (L1) gave the preference of N1 product, whereas DPEphos (L2) gave exclusive formation of the desired N2 substituted benzotriazole (Scheme 1 b). Intrigued by this fact, we performed additional experiments^5^ to probe the reaction mechanism and proposed the catalytic cycle illustrated in Scheme 2. The reaction involves three steps and begins with oxidative addition of benzotriazole tautomers **1 a**/**b** to the Rh^I^ complex. The generated Rh^III^−H intermediates A and B proceed through hydrometalation and transform into σ‐allyl complexes C and D. Intermediates C or D then undergo reductive elimination with the contaminant release of N‐allylated products **4** and **3**, respectively.

**Scheme 2:**
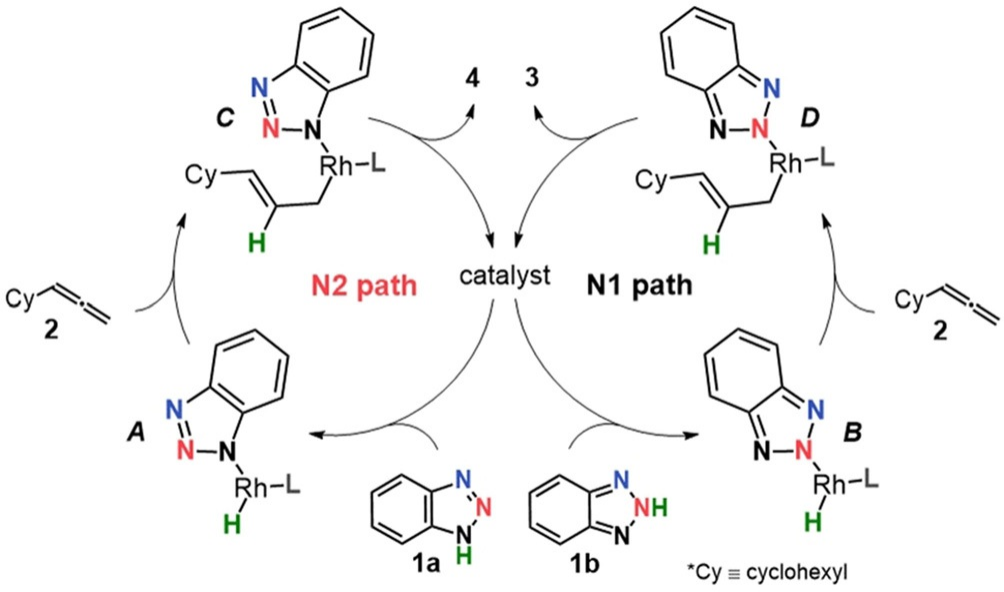
Proposed reaction mechanism for Rh catalyzed coupling of 1,2,3‐benzotriazoles with allenes.

Despite the plausibility of the reaction mechanism outlined in the Scheme 2, the factors determining the unusual N2 regioselectivity remain unclear. This fact has motivated us to study the reaction mechanism using density functional theory (DFT) calculations. To provide general concepts for the rational design of other complementary approaches for the regioselective synthesis of valuable N‐substituted synthons, the following questions must be addressed: 1) What is the reaction mechanism? 2) Which step of the catalytic cycle is regiodetermining? 3) What are the physical factors behind the regioselectivity? To answer these questions, we have performed a systematic in‐depth theoretical investigation based on the accounts of the previously reported experimental data.^5^

The coordination of Rh to L1′ was initially investigated to determine the active form of the catalyst. Figure 1 illustrates the two possible binding modes of the JoSPOphos ligand (L1′) to the Rh center.^6^ The large difference in computed Gibbs free energies (ΔΔ*G*=17.4 kcal mol^−1^) results from a favorable P‐type of binding in Rh‐L1′. Having determined the structure of the catalyst, we then investigated the reaction mechanism.

**Figure 1:**
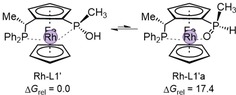
Coordination modes of L1′ to the Rh. To reduce computational cost the *tert*‐butyl group of the ligand (L1) was replaced with methyl (L1′). Relative energies [kcal mol^−1^] were computed at SMD(DCE)‐B3LYP‐D3/6‐31G(d)/LANL2DZ.

All relevant mechanistic pathways (for details, see the Supporting Information, Figure S1) were computationally^7^ explored and the Gibbs free energy profiles of the most favorable catalytic process were constructed (Scheme 3 a). DFT calculations revealed a kinetic preference for N1 allylation (black color pathway) over N2, which is in line with experimental results of N1‐substituted benzotriazole (**3**) being the major product when JoSPOphos is employed.

**Scheme 3:**
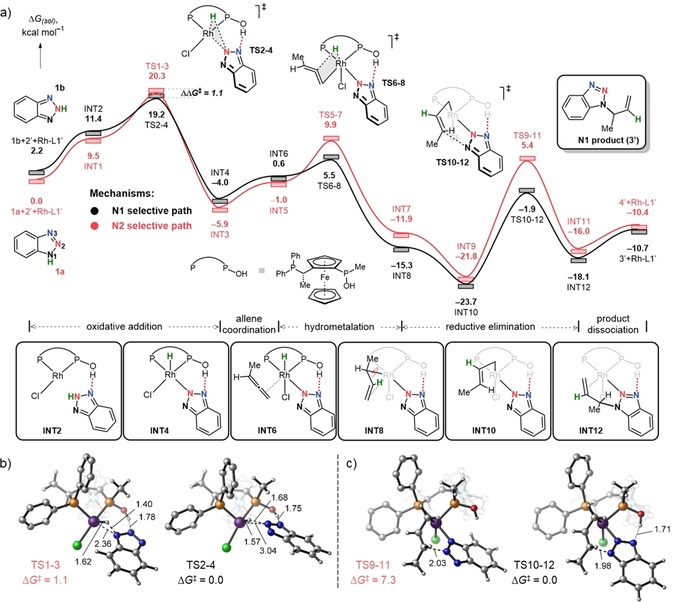
(a) Gibbs free energy profile for coupling of the 1,2,3‐benzotriazoles with allenes catalyzed by Rh‐L1′ at 80 °C and a standard state of 1 mol L^−1^; (b) Regioselectivity determining transition states; (c) Rate‐determining transition states. Bond lengths in Å, Gibbs free energies in kcal mol^−1^. To reduce computational cost the cyclohexyl group of allene (2) was replaced by the methyl group (2′) and *tert*‐butyl group of the ligand (L1) was replaced with methyl (L1′). All data computed at SMD(DCE)‐B3LYP‐D3/6‐311++G(d,p)/SDD//SMD(DCE)‐B3LYP‐D3/6‐31G(d)/LANL2DZ.

The reaction is initiated by coordination of the substrate **1 a**/**b** to Rh‐L1′ to form the pre‐reactant complexes INT1 and INT2. Notably, the hydroxy group on the Rh‐L1′ catalyst acts as a directing group for the incoming benzotriazole and results in an OH⋅⋅⋅N1/N2 hydrogen‐bonding interaction. Either the N1−H (**1 a** tautomer) or N2−H bond (**1 b** tautomer) can undergo oxidative addition depending on the tautomeric form of the benzotriazole. The oxidative addition involves the classical 3‐membered TSs (TS1‐3, TS2‐4) with relative free energies of 20.3 and 19.2 kcal mol^−1^. Computed structures of TS1‐3 and TS2‐4 are illustrated in Scheme 3 b and reveal a late, product‐like character for TS1‐3 with a nearly formed Rh−N bond at 2.36 Å and an earlier TS structure for TS2‐4, in which the Rh−N bond is still relatively long 3.04 Å. Because formation of INT3 and INT4 is downhill with respect to INT1, INT2 by 15.4 kcal mol^−1^ in both cases, the oxidative addition step is predicted to be irreversible. To provide a coordination site necessary for the subsequent hydrometalation of the allene, the Cl ligand migrates to an axial position, whereas the allene binds to the Rh via an external C=C double bond in the equatorial plane. This ligand transformation raises energies to −1.0 and 0.6 kcal mol^−1^ to form INT5 and INT6, respectively. The catalytic cycle continues with the hydrometalation and passes over transition states TS5‐7, TS6‐8 with Gibbs free activation barriers of 8.9 and 4.9 kcal mol^−1^, respectively. The resulting intermediates INT7, INT8 are asymmetrical π‐type complexes located at −11.9 and −15.3 kcal mol^−1^, which further isomerize to more stable σ‐allyl intermediates^8^ INT9, INT10 with relative free energies of −21.8 and −23.7 kcal mol^−1^. The reductive elimination traversing transition states TS9‐11, TS10‐12 and affords the product complexes INT11, INT12. It is worth pointing out that the calculated activation barrier of reductive elimination for the N1‐selective pathway (Scheme 3 a, in black) is significantly lower (ΔΔ*G* ^≠^ *=*5.4 kcal mol^−1^) compared to the N2 route. The optimized transition structures (Scheme 3 c) reveal a stabilizing hydrogen‐bonding interaction in TS10‐12 that is absent in TS9‐11, which may be the origin of the lower barrier for the former. Release of products **3′** and **4′** allows the Rh catalyst to reenter the catalytic cycle and bind a new substrate. The Gibbs free energy profile provided in Scheme 3 a reveals that the oxidative addition is the regioselectivity determining step, while the reductive elimination is the rate‐determining step. Our DFT calculations support the experimental observation^5^ of N1‐allylated regioisomer (**3′**) being a preferred product as a TS2‐4 associated with N1‐selective path is more stable than a competitive TS1‐3 for 1.1 kcal mol^−1^.

To understand why simply changing the JoSPOphos ligand (L1) with DPEphos (L2) completely switches the regioselectivity from N1 to N2, we again sampled various mechanistic possibilities (see the Supporting Information, Figures S2, S3) and outlined the lowest‐energy reaction profiles corresponded to N1 and N2 pathways (Scheme 4 a). In contrast to L1′ (Scheme 3 a), now the N2 reaction channel (red curve, Scheme 4 a) is kinetically favored, which is in line with the experimental regioselective allylation at N2 atom of the benzotriazole.

**Scheme 4:**
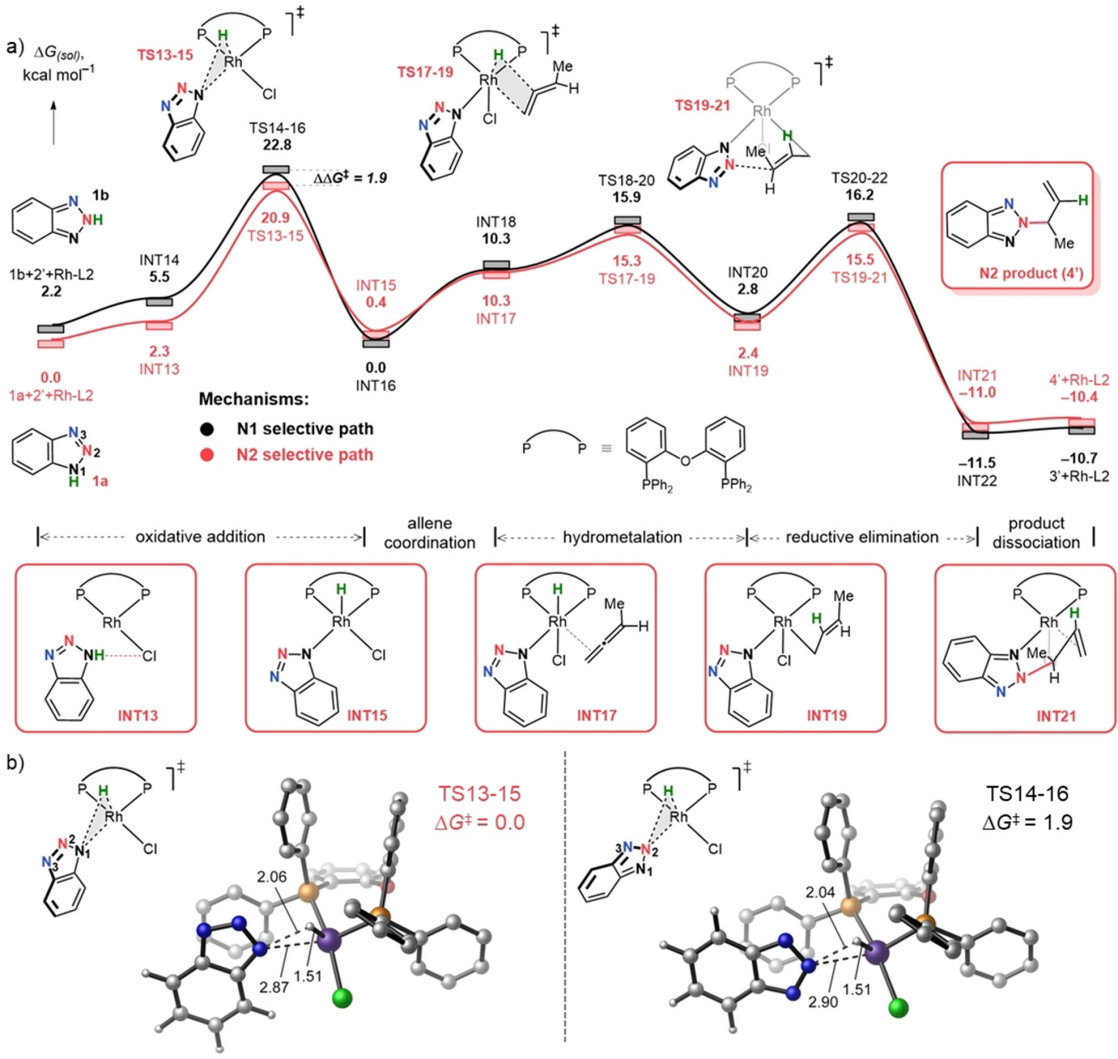
(a) Gibbs free energy profile for coupling of the 1,2,3‐benzotriazoles with allenes catalyzed by Rh‐L2 at 80 °C and a standard state of 1 mol L^−1^; (b) Regioselectivity determining transition states. Bond lengths in Å, Gibbs free energies in kcal mol^−1^. To reduce computational cost the cyclohexyl group of allene (2) was replaced by the methyl group (2′). All data computed at SMD(DCE)‐B3LYP‐D3/6‐311++G(d,p)/SDD//SMD(DCE)‐B3LYP‐D3/6‐31G(d)/LANL2DZ.

The first elementary step of the catalytic cycle (Scheme 4 a), namely, oxidative addition takes place from INT13 or INT14, in which the substrates **1 a** or **1 b** are pre‐coordinated to the catalyst via a weak hydrogen‐bond interaction between N1/2−H⋅⋅⋅Cl. From the substrate‐coordinated complexes INT13 and INT14, the N1/N2−H activation is taking place via TS13‐15/TS14‐16 located at 20.9 and 22.8 kcal mol^−1^. Optimized structures of TS13‐15, TS14‐16 are provided in Scheme 4 b and are late with respect to the N−H bond breaking, displaying N−H distances of approximately 2.0 Å. The Rh^III^ hydride intermediates (INT15, INT16) furnished after the oxidative addition adopt a pentacoordinate structure bearing a hydride in the apical and benzotriazole fragment in the equatorial positions. The formation of these species is exergonic relative to the pre‐reactive complexes (INT13, INT14) by 1.9 and 5.5 kcal mol^−1^, respectively, suggesting oxidative addition to be irreversible. Binding of the incoming allene **2′** during the second stage of the catalytic cycle is uphill in terms of Gibbs free energy by 9.9 (INT17) and 10.3 (INT18) kcal mol^−1^. During this process, the coordination geometry at the Rh center changes from square pyramidal to octahedral, in which Cl and H ligands occupy apical positions. The reaction continues with a fast hydrometalation process via TS17‐19, TS18‐20 with activation free energies of 6.0 and 6.9 kcal mol^−1^ to give σ‐allyl complexes (INT19, INT20). Finally, the hydrometalation adducts undergo reductive elimination with barriers of 13.1 and 13.4 kcal mol^−1^ (TS19‐21 and TS20‐22), and deliver the product complexes INT21 and INT22. The catalytic cycle completes upon dissociation of the *N*‐allylated products **3′** or **4′** from the coordination sphere of the metal with the regeneration of the catalyst. Considering the full Gibbs free energy profile illustrated in Scheme 4 a, the key step determining the regioselectivity of the allylation is the oxidative addition step. Our DFT results show TS13‐15 to be more stable than TS14‐16 by 1.9 kcal mol^−1^. This ΔΔ*G* ^≠^ between the regiodetermining TSs corresponds to a ratio of N1/N2 4:96 with predominance of N2 allylated regioisomer **4′**, which is in perfect agreement with the experimentally isolated ratio of N1/N2 6:94.

To understand why DPEphos (L2) leads to N2 regioselectivity, we quantitatively analyzed the regiodetermining oxidative addition step catalyzed by Rh‐L2 using the activation strain model ASM (Figure 2).^9^ Within the ASM, the relative energy in solution Δ*E*_solution_ along the reaction energy profile is separated into the energy of the solute Δ*E*_solute_ (reaction system in vacuum with the solution phase geometry) and the solvation energy Δ*E*_solvation_[Eq. (1)].

(1) $$\Delta E{}_{\mathrm{solution}}=\Delta E{}_{\mathrm{solute}}+\Delta E{}_{\mathrm{solvation}}$$

**Figure 2:**
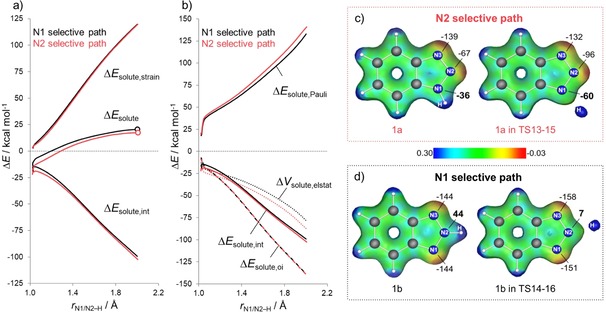
(a) Activation strain analyses and (b) energy decomposition analyses for the oxidative addition of 1,2,3‐benzotriazole **1 a** (N2‐selective pathway) or **1 b** (N1‐selective pathway) to Rh‐L2 computed at ZORA‐B3LYP‐D3/TZ2P level. Dots represent TSs. Electrostatic potential maps along with the Hirshfeld charges (m a.u.) at N1, N2, N3 atoms of the 1,2,3‐benzotriazole fragment (1a/1b) in both equilibrium (1a/1b) and transition state (TS13‐15/TS14‐16) geometries correspond to (c) N2‐ and (d) N1‐selective pathways. ESP maps are plotted on the total electron density of the 1,2,3‐benzotriazole fragment from the ZORA‐B3LYP‐D3/TZ2P calculations by using a consistent surface potential range of −0.03 a.u. to 0.30 a.u. and isovalue 0.045.

The intrinsic energy of the solute Δ*E*_solute_ is split further into two terms: Δ*E*_solute,strain_ (energy required for deformation of fragments from their starting geometries to the geometries they obtain over the course of the reaction) and Δ*E*_solute,int_ (energy of interaction between deformed fragments) [Eq. (2)].

(2) $$\Delta E{}_{\mathrm{solute}}=\Delta E{}_{\mathrm{solute},\mathrm{strain}}+\Delta E{}_{\mathrm{solute},\mathrm{int}}$$

Next, the interaction energy Δ*E*_solute,int_ is decomposed into three terms using a canonical energy decomposition analysis (EDA) [Eq. (3)]:

(3) $$\Delta E{}_{\mathrm{solute},\mathrm{int}}=\Delta V{}_{\mathrm{solute},\mathrm{elstat}}+\Delta E{}_{\mathrm{solute},\mathrm{Pauli}}+\Delta E{}_{\mathrm{solute},\mathrm{oi}}$$

in which Δ*V*_solute,elstat_ corresponds to the electrostatic interaction between unperturbed charges of the deformed reactants, Δ*E*_solute,Pauli_ accounts for the repulsion between occupied orbitals, Δ*E*_solute,oi_ is responsible for interaction between occupied and unoccupied orbitals and polarization. The ASM and EDA terms were projected on the N1/N2−H bond stretch, as this geometrical parameter is critically involved in the reaction and undergoes a well‐defined change^9^ over the course of the oxidative addition step.

Figure 2 a shows that the interaction energies (Δ*E*_solute,int_) control the reaction regiochemistry, whereas the strain energy required for deformation of catalyst (Rh‐L2) and substrate (one of the two 1,2,3‐benzotriazole tautomers, **1 a**/**b**) during oxidative addition (Δ*E*_solute,strain_) is nearly identical for both N1‐ (black) and N2‐selective (red) pathways. Similar Δ*E*_solute,strain_ curves is a consequence of breaking the same N−H bond at either N1 or N2 during the oxidative addition. Because the Δ*E*_solute,int_ is decisive in determining the trend in Δ*E*_solute_ and thus the observed reactivity trends, it was further analyzed by using the EDA, and the results are plotted in Figure 2 b. Differences in the orbital‐interaction curves (Δ*E*_solute,oi_) are minimal. Thus, it becomes evident that the predominance of the N2‐selective pathway can be attributed to the more stabilizing electrostatic interaction (Δ*V*_solute,elstat_), which effectively overrules the less destabilizing Pauli repulsion (Δ*E*_solute,Pauli_) preference for the N1 pathway.

Next, to understand the trend in Δ*V*_solute,elstat_, we examined the electrostatic potential maps (ESP) and Hirshfeld charges^10^ of Rh and N1, N2, N3 atoms of 1,2,3‐benzotriazole fragments (**1 a**/**b**). Figure 2 c and d illustrate the ESP and charge analysis for the 1,2,3‐benzotriazole tautomers at their equilibrium and TSs geometries. Our analysis has identified a causal relationship between the charge of the N atom of the benzotriazole involved in the regiodetermining oxidative addition step and the degree of electrostatic stabilization: a more electronegative N atom leads to a stronger, more stabilizing electrostatic interaction with the electropositive Rh metal center. Thus, the more reactive tautomer **1 a** has an N1 atom positioned between a carbon atom and nitrogen N2. This results in a relatively electronegative N1 (−36 m a.u.), which leads to a strongly stabilizing electrostatic interaction with Rh (20 m a.u.) metal center (Figure 2 c). In contrast, the less reactive tautomer **1 b** has a partially positive (44 m a.u.) N2 atom (Figure 2 d) as a result of being positioned between two nitrogens (N1 and N3 atoms). This leads to less stabilizing electrostatic interaction between the N2 atom and the electropositive Rh. The electrostatic interactions are more stabilizing for the reaction of N2 compared with N1 over the entire reaction coordinate (Figure 2 b), thus this charge and molecular electrostatic potential (MEP) analysis can be performed at any point.

In summary, we have computationally analyzed the reaction mechanism of a newly developed coupling of 1,2,3‐benzotriazoles with allenes catalyzed by Rh‐diphosphine ligand (JoSPOphos/DPEphos) complexes. Our DFT calculations reveal that regardless of the ligand being used, the reaction proceeds via a three‐step catalytic cycle, comprised of first an oxidative addition, then hydrometalation, and finally a reductive elimination. Our activation strain and energy decomposition analyses on the regiodetermining oxidative addition step catalyzed by Rh‐DPEphos show that the preference for the N2‐selective reaction channel arises from a previously unrecognized electronic mechanism, namely, a more stabilizing electrostatic interaction between the electron‐enriched region at the N1 atom of tautomer **1 a** with the positively charged Rh during N1−Rh bond formation in the rate‐determining N1−H oxidative addition step. We envisage that the newly identified electrostatic interactions can be used to further tune the 1,2,3‐benzotriazole and catalyst interaction through the introduction of various functionalities, which may allow new reactions with tailored N2 regioselectivity trend.

## Conflict of interest

The authors declare no conflict of interest.

## Supporting information

As a service to our authors and readers, this journal provides supporting information supplied by the authors. Such materials are peer reviewed and may be re‐organized for online delivery, but are not copy‐edited or typeset. Technical support issues arising from supporting information (other than missing files) should be addressed to the authors.

Supplementary

## References

[1] For selected reviews, see:

[1a] L. Huang , M. Arndt , K. Gooßen , H. Heydt , L. J. Gooßen , Chem. Rev. 2015, 115, 2596–2697; 25721762 10.1021/cr300389u

[1b] P. Koschker , B. Breit , Acc. Chem. Res. 2016, 49, 1524–1536; 27455048 10.1021/acs.accounts.6b00252

[1c] H. J. Davis , R. J. Phipps , Chem. Sci. 2017, 8, 864–877; 28572898 10.1039/c6sc04157d PMC5452277

[1d] L. Ping , D. S. Chung , J. Bouffard , S. Lee , Chem. Soc. Rev. 2017, 46, 4299–4328. 28537608 10.1039/c7cs00064b

[2a] W. Roth , D. Spangenberg , Ch. Janzen , A. Westphal , M. Schmitt , Chem. Phys. 1999, 248, 17–25;

[2b] N. Jagerovic , M. L. Jimeno , I. Alkorta , J. Elguero , R. M. Claramunt , Tetrahedron 2002, 58, 9089–9094;

[2c] L. T. Ueno , R. O. Ribeiro , M. S. Rocha , M. E. V. Suárez-Iha , K. Iha , F. B. C. Machado , J. Mol. Struct. 2003, 664, 207–215;

[2d] J. Poznański , A. Najda , M. Bretner , D. Shugar , J. Phys. Chem. A 2007, 111, 6501–6509. 17585743 10.1021/jp071611h

[3] N1-selective strategies:

[3a] N. Arnau , Y. Arredondo , M. Moreno-Mañas , R. Pleixats , M. Villarroya , J. Heterocycl. Chem. 1995, 32, 1325–1334;

[3b] W. Yan , X. Ye , K. Weise , J. L. Petersen , X. Shi , Chem. Commun. 2012, 48, 3521–3523; 10.1039/c2cc17522c 22382692

[3c] S. Gupta , N. Chandna , A. K. Singh , N. Jain , J. Org. Chem. 2018, 83, 3226–3235. N2-selective strategies:29463081 10.1021/acs.joc.8b00107

[3d] A. R. Katritzky , W. Kuzmierkiewicz , J. V. Greenhill , Recl. Trav. Chim. Pays-Bas. 2010, 110, 369–373;

[3e] J. Wen , L. L. Zhu , Q.-W. Bi , Z.-Q. Shen , X.-X. Li , X. Li , Z. Wang , Z. Chen , Chem. Eur. J. 2014, 20, 974–978; 24375713 10.1002/chem.201302761

[3f] K. Wang , P. Chen , D. Ji , X. Zhang , G. Xu , J. Sun , Angew. Chem. Int. Ed. 2018, 57, 12489–12493; 10.1002/anie.201807039 30094906

[3g] L.-L. Zhu , L. Tian , H. Zhang , L. Xiao , W. Luo , B. Cai , H. Wang , C. Wang , G. Liu , C. Pei , Y. Wang , Adv. Synth. Catal. 2019, 361, 1117–1123.

[4a] I. Briguglio , S. Piras , P. Corona , E. Gavini , M. Nieddu , G. Boatto , A. Carta , Eur. J. Med. Chem. 2015, 97, 612–648; 25293580 10.1016/j.ejmech.2014.09.089 PMC7115563

[4b] P. Worthington in Bioactive Heterocyclic Compound Classes: Agrochemicals (Eds.: C. Lamberth, J. Dinges), Wiley-VCH, New York, 2012, pp. 129–145;

[4c] Y. Cui , Y. Wu , X. Lu , X. Zhang , G. Zhou , F. B. Miapeh , W. Zhu , Z.-S. Wang , Chem. Mater. 2011, 23, 4394–4401.

[5] K. Xu , N. Thieme , B. Breit , Angew. Chem. Int. Ed. 2014, 53, 7268–7271; 10.1002/anie.201403682 24863853

[6] H. Landert , F. Spindler , A. Wyss , H.-U. Blaser , B. Pugin , Y. Ribourduoille , B. Gschwend , B. Ramalingam , A. Pfaltz , Angew. Chem. Int. Ed. 2010, 49, 6873–6876; 10.1002/anie.201002225 20715032

[7] Geometry optimizations and frequency analysis were performed in Gaussian 09^[11]^ at the B3LYP-D3^[12]^ level of theory with a mixed basis set of LANL2DZ^[13]^ ECP for Rh and 6-31G(d)^[14]^ for other atoms in implicit solvent DCE treated with SMD solvation model.^[15]^ Single-point energies were computed at B3LYP-D3 level of theory with the SDD^[16]^ basis set for Rh, and 6–311++G(d,p) for the other atoms in DCE. Gibbs free energies are reported at 80 °C and a standard state of 1 mol L^−1^ Grimme's quasi-harmonic approximation was used to compute the entropic contributions to the Gibbs free energies.^[17]^ To understand the factors governing reaction regioselectivity the activation strain model (ASM)^[9]^ and energy-decomposition analysis (EDA) were applied using ADF.2017.103^[18]^ and PyFrag programs^[19]^ at ZORA^[20]^-B3LYP-D3/TZ2P^[21]^ on SMD(DCE)-B3LYP-D3/6-31G(d)/LANL2DZ geometries. To reduce computational cost, the cyclohexyl group of the allene (2) was replaced by the methyl (2′).

[8] U. Gellrich , A. Meißner , A. Steffani , M. Kähny , H.-J. Drexler , D. Heller , D. A. Plattner , B. Breit , J. Am. Chem. Soc. 2014, 136, 1097–1104. 24377792 10.1021/ja411204d

[9] For reviews on the activation strain analysis, see:

[9a] F. M. Bickelhaupt , K. N. Houk , Angew. Chem. Int. Ed. 2017, 56, 10070–10086; 10.1002/anie.201701486 PMC5601271 28447369

[9b] L. P. Wolters , F. M. Bickelhaupt , WIRES Comput. Mol. Sci. 2015, 5, 324–343; 10.1002/wcms.1221 PMC4696410 26753009

[9c] I. Fernańdez , F. M. Bickelhaupt , Chem. Soc. Rev. 2014, 43, 4953–4967; for the activation strain analysis in the condensed phase, see:24699791 10.1039/c4cs00055b

[9d] T. A. Hamlin , B. van Beek , L. P. Wolters , F. M. Bickelhaupt , Chem. Eur. J. 2018, 24, 5927–5938. 29457865 10.1002/chem.201706075 PMC5947303

[10] B. J. Levandowski , T. A. Hamlin , H. J. Eckvahl , F. M. Bickelhaupt , K. N. Houk , J. Mol. Model. 2019, 25, 33/1–5. 30627945 10.1007/s00894-018-3909-z

[11] M. J. Frisch et al. Gaussian 09, Revision D.01, Gaussian Inc.: Wallingford, CT, **2009**.

[12a] A. D. Becke , J. Chem. Phys. 1993, 98, 5648–5652;

[12b] C. Lee , W. Yang , R. G. Parr , Phys. Rev. B: Condens. Matter Mater. Phys. 1988, 37, 785–789; 10.1103/physrevb.37.785 9944570

[12c] A. D. Becke , J. Chem. Phys. 1993, 98, 1372–1377;

[12d] P. J. Stephens , F. J. Devlin , C. F. Chabalowski , M. J. Frisch , J. Phys. Chem. 1994, 98, 11623–11627;

[12e] T. Sperger , I. A. Sanhueza , I. Kalvet , F. Schoenebeck , Chem. Rev. 2015, 115, 9532–9586; 26207572 10.1021/acs.chemrev.5b00163

[12f] S. Grimme , J. Antony , S. Ehrlich , H. Krieg , J. Chem. Phys. 2010, 132, 154104–154119. 20423165 10.1063/1.3382344

[13] L. E. Roy , P. J. Hay , R. L. Martin , J. Chem. Theory Comput. 2008, 4, 1029–1031. 26636355 10.1021/ct8000409

[14a] R. Ditchfield , W. J. Hehre , J. A. Pople , J. Chem. Phys. 1971, 54, 724–728;

[14b] W. J. Hehre , R. Ditchfield , J. A. Pople , J. Chem. Phys. 1972, 56, 2257–2261.

[15] A. V. Marenich , C. J. Cramer , D. G. Truhlar , J. Phys. Chem. B 2009, 113, 6378–6396. 19366259 10.1021/jp810292n

[16a] U. Häussermann , M. Dolg , H. Stoll , H. Preuss , P. Schwerdtfeger , R. M. Pitzer , Mol. Phys. 1993, 78, 1211–1224;

[16b] W. Küchle , M. Dolg , H. Stoll , H. Preuss , J. Chem. Phys. 1994, 100, 7535–7542.

[17a] I. Funes-Ardoiz, R. S. Paton, *Goodvibes: Goodvibes v2.0.1*, **2016**, https://doi.org/10.5281/zenodo.595246;

[17b] S. Grimme , Chem. Eur. J. 2012, 18, 9955–9964. 22782805 10.1002/chem.201200497

[18a] G. te Velde , F. M. Bickelhaupt , E. J. Baerends , C. Fonseca Guerra , S. J. A. van Gisbergen , J. G. Snijders , T. Ziegler , J. Comput. Chem. 2001, 22, 931–967;

[18b] C. Fonseca Guerra , J. G. Snijders , G. te Velde , E. J. Baerends , Theor. Chem. Acc. 1998, 99, 391–403;

[18c] ADF2017, SCM Theoretical Chemistry; Vrije Universiteit, Amsterdam, The Netherlands, http://www.scm.com.

[19] X. Sun , T. M. Soini , J. Poater , T. A. Hamlin , F. M. Bickelhaupt , J. Comput. Chem. 2019, 40, 2227–2233. 31165500 10.1002/jcc.25871 PMC6771738

[20a] E. van Lenthe , E. J. Baerends , J. G. Snijders , J. Chem. Phys. 1994, 101, 9783–9792;

[20b] E. van Lenthe , R. van Leeuwen , E. J. Baerends , J. G. Snijders , Int. J. Quantum Chem. 1996, 57, 281–293.

[21] E. van Lenthe , E. J. Baerends , J. Comput. Chem. 2003, 24, 1142–1156. 12759913 10.1002/jcc.10255

