# Supplementary material for: Ligand‐Mediated Regioselective Rhodium‐Catalyzed Benzotriazole–Allene Coupling: Mechanistic Exploration and Quantum Chemical Analysis
Source: Chemistry. 2020 Feb 4;26(11):2342–8. doi: 10.1002/chem.201905359 (PMC7064967; doi:10.1002/chem.201905359)
Supplement: Supplementary file 1 — Supplementary [file CHEM-26-2342-s001.pdf]

CHEMISTRY
A European Journal

Supporting Information

Ligand-Mediated Regioselective Rhodium-Catalyzed Benzotriazole–Allene Coupling: Mechanistic Exploration and Quantum Chemical Analysis

Tetiana Sergeieva,[a, b] Trevor A. Hamlin,[b] Sergiy Okovytyy,[c] Bernhard Breit,*[a] and F. Matthias Bickelhaupt*[b, d]

chem_201905359_sm_miscellaneous_information.pdf

Contents

Figure S1. Gibbs free energy profile for competing pathways occurring at the first step of the catalytic cycle for coupling of the 1,2,3-benzotriazoles with allenes catalyzed by Rh-L1'.

Figure S2. Gibbs free energy profile for competing pathways occurring at the first step of the catalytic cycle for coupling of the 1,2,3-benzotriazoles with allenes catalyzed by Rh-L2.

Figure S3. Gibbs free energy profile for alternative mechanisms (in-sphere, out-of-sphere) of the reductive elimination for coupling of the 1,2,3-benzotriazoles with allenes catalyzed by Rh-L2.

Cartesian coordinates and energies

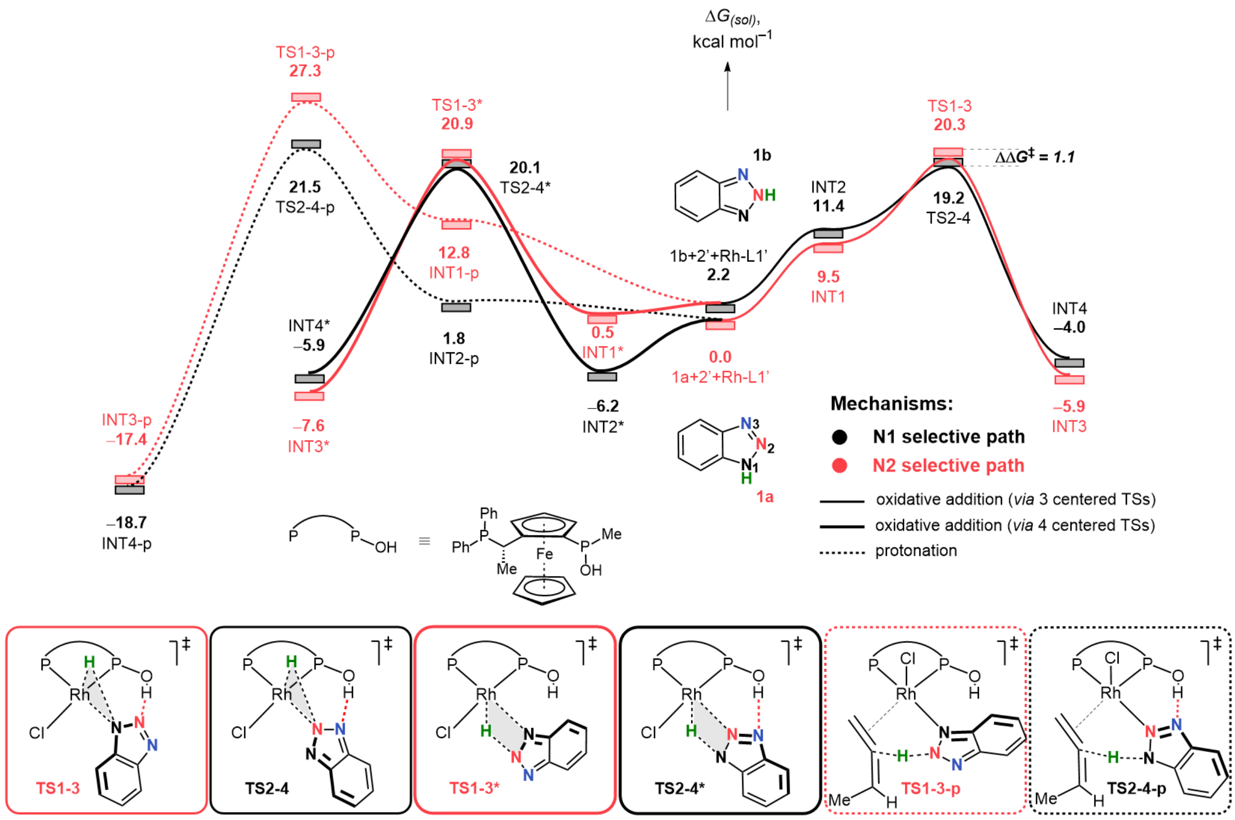

Figure S1. Gibbs free energy profile for competing pathways of the first step of the catalytic cycle for coupling of the 1,2,3-benzotriazoles with allenes catalyzed by Rh-L1' at 80 °C and a standard state of 1 mol L^{-1}; Gibbs free energies in kcal mol^{-1}. To reduce computational cost the cyclohexyl group of allene (2) was replaced by the methyl group (2'). All data computed at SMD(DCE)-B3LYP-D3/6-311++G(d,p)/SDD//SMD(DCE)-B3LYP-D3/6-31G(d)/LanL2DZ.

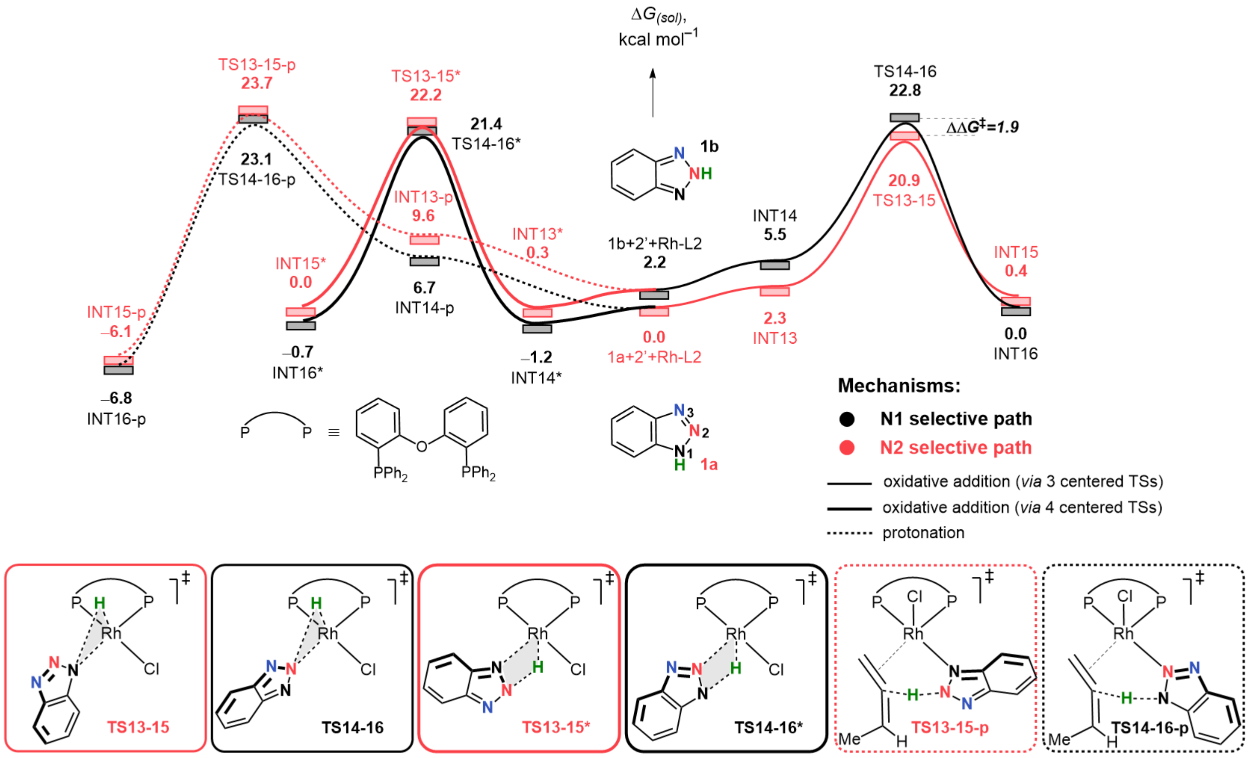

Figure S2. Gibbs free energy profile for competing pathways of the first step of the catalytic cycle for coupling of the 1,2,3-benzotriazoles with allenes catalyzed by Rh-L2 at 80 °C and a standard state of 1 mol L^{-1}; Gibbs free energies in kcal mol^{-1}. To reduce computational cost the cyclohexyl group of allene (2) was replaced by the methyl group (2'). All data computed at SMD(DCE)-B3LYP-D3/6-311++G(d,p)/SDD//SMD(DCE)-B3LYP-D3/6-31G(d)/LanL2DZ.

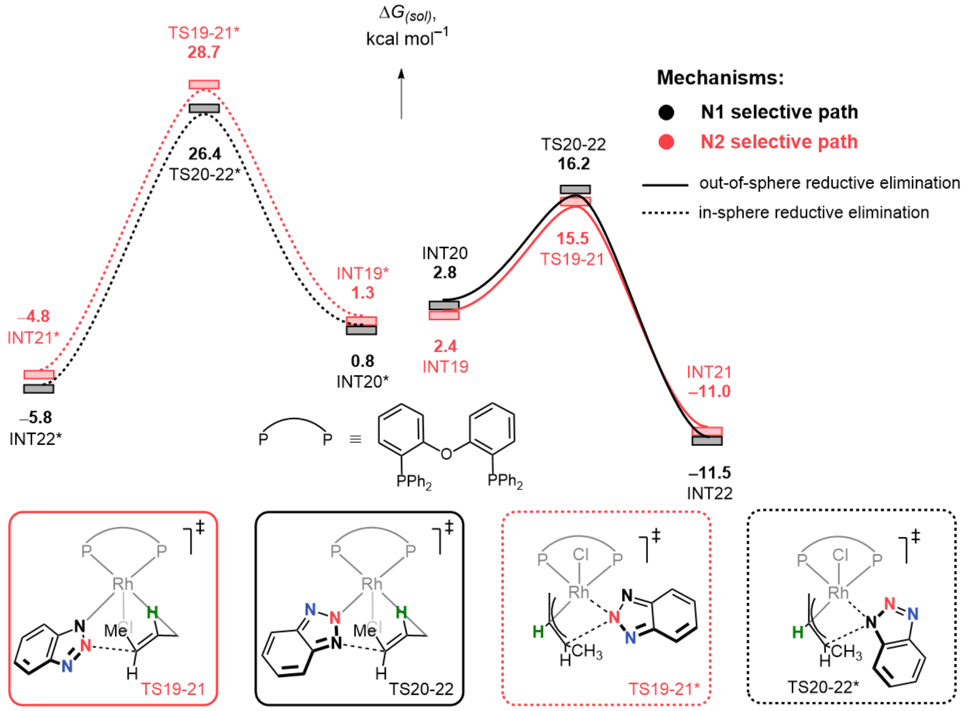

Figure S3. Gibbs free energy profile for alternative mechanisms (in-sphere, out-of-sphere) of the reductive elimination for coupling of the 1,2,3-benzotriazoles with allenes catalyzed by Rh-L2 at 80 °C and a standard state of 1 mol L^{-1}. Gibbs free energies in kcal mol^{-1}. To reduce computational cost the cyclohexyl group of allene (2) was replaced by the methyl group (2'). All data computed at SMD(DCE)-B3LYP-D3/6-311++G(d,p)/SDD//SMD(DCE)-B3LYP-D3/6-31G(d)/LanL2DZ. Gibbs free energies are relative to the sum of energies of separated 1,2,3-benzotriazole (1a), allene (2'), catalyst (Rh-L2).

Cartesian coordinates and energies

Rh-L1'

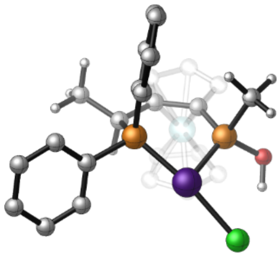

SMD(DCE)-B3LYP-D3/6-31G(d)/LanL2DZ
Thermal correction to Gibbs Free Energy = 0.385707
Sum of electronic and thermal Free Energies = -2419.247233
SMD(DCE)-B3LYP-D3/6-311++G(d,p)/SDD
Single point energy = -2421.5773719
Corrected quasi-harmonic Free Energy = -2421.198454
N_{imag} = 0

Cl	0.579282	-2.348943	3.320498
P	-1.438181	-0.049286	-0.261875
C	1.519458	1.257194	0.053125
C	0.990647	0.992631	-1.272041
C	-0.223902	0.168139	-1.673309
H	0.101401	-0.871555	-1.798506
C	2.701557	2.060972	-0.094479
C	1.862745	1.645769	-2.200566
C	2.911660	2.290886	-1.480808
H	3.745523	2.824254	-1.918845
H	1.768571	1.617689	-3.277211
H	3.342448	2.386761	0.714119
C	-0.849406	0.617245	-3.004525
H	-0.132538	0.473967	-3.820316
H	-1.143339	1.670315	-2.992333
H	-1.733118	0.017065	-3.239832
Fe	2.904634	0.246613	-1.065001
C	4.827340	-0.518308	-1.243290
C	4.238738	-0.931066	-0.008585
C	3.055317	-1.673857	-0.310366
C	4.005801	-1.000570	-2.308243
C	2.911036	-1.715123	-1.730170
H	4.163234	-0.822035	-3.364342
H	2.089483	-2.167477	-2.271328
H	2.352937	-2.073880	0.410138
H	4.589870	-0.678342	0.982655
H	5.713735	0.093638	-1.352556
C	-2.678350	-1.207896	-0.967200
C	-3.872140	-0.789810	-1.576844
C	-2.383235	-2.581516	-0.907518
C	-4.755343	-1.730889	-2.109978
H	-4.114022	0.266448	-1.637260
C	-3.267725	-3.519037	-1.444099
H	-1.455717	-2.916152	-0.446410
C	-4.456657	-3.094789	-2.043507
H	-5.676566	-1.396556	-2.579488
H	-3.028380	-4.577620	-1.391749
H	-5.147604	-3.823677	-2.458313
C	-2.316313	1.546631	-0.027536
C	-3.482388	1.551938	0.758941
C	-1.801932	2.768384	-0.488318
C	-4.132037	2.749493	1.055135

H	-3.884613	0.615401	1.136437
C	-2.451370	3.968207	-0.182743
H	-0.887679	2.799859	-1.070100
C	-3.617439	3.962339	0.584611
H	-5.036303	2.736600	1.657501
H	-2.040765	4.906323	-0.546256
H	-4.121129	4.896002	0.819662
P	0.892972	0.659730	1.640711
C	0.349195	2.186692	2.492192
H	1.183903	2.894129	2.559080
H	-0.472190	2.650060	1.938414
H	0.005731	1.930136	3.498907
O	2.261749	0.305351	2.497950
H	2.183738	-0.623005	2.803509
Rh	-0.492495	-0.998438	1.533346

Rh-L1'a

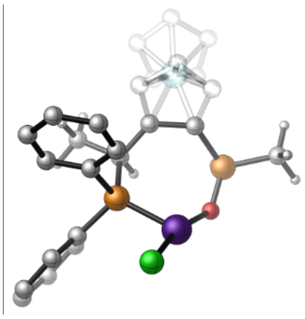

SMD(DCE)-B3LYP-D3/6-31G(d)/LanL2DZ
Thermal correction to Gibbs Free Energy = 0.382756
Sum of electronic and thermal Free Energies = -2419.219529
SMD(DCE)-B3LYP-D3/6-311++G(d,p)/SDD
N_{imag} = 0

Rh	1.519110	1.890017	0.749062
Cl	3.517712	1.429166	1.991624
P	1.436375	-0.081703	-0.179791
C	-1.921179	0.719760	0.539930
C	-1.280469	-0.467198	0.023253
C	-0.211982	-0.507935	-1.034143
H	-0.371590	0.319894	-1.733902
C	-2.802070	0.328572	1.606160
C	-1.773844	-1.568662	0.784872
C	-2.706517	-1.082664	1.747564
H	-3.277772	-1.688873	2.438284
H	-1.510094	-2.605512	0.632195
H	-3.445190	0.986855	2.174391
C	-0.204193	-1.827588	-1.816871
H	-1.184228	-1.973259	-2.284456
H	-0.013275	-2.684476	-1.165279
H	0.556722	-1.823397	-2.601103
Fe	-3.340921	-0.579144	-0.176055
C	-5.345579	-0.163619	-0.536069
C	-4.532230	0.398894	-1.566488
C	-3.811502	-0.663686	-2.193080
C	-5.127395	-1.575788	-0.524612
C	-4.180191	-1.884758	-1.548306
H	-5.567002	-2.281704	0.168217
H	-3.780641	-2.866165	-1.769061
H	-3.087705	-0.557496	-2.990464
H	-4.447242	1.451117	-1.806423
H	-5.983790	0.387464	0.142625
C	2.706656	-0.259733	-1.500744

C	4.014492	-0.652336	-1.168536
C	2.428626	0.119747	-2.824872
C	5.012151	-0.690033	-2.143832
H	4.255387	-0.923355	-0.145553
C	3.428687	0.079998	-3.798413
H	1.433207	0.450833	-3.106443
C	4.722322	-0.327881	-3.462267
H	6.017440	-0.999651	-1.870445
H	3.195329	0.369781	-4.819590
H	5.499979	-0.357487	-4.220792
C	1.675887	-1.534812	0.926608
C	1.281150	-1.416790	2.268362
C	2.145910	-2.773914	0.461328
C	1.349417	-2.514954	3.127173
H	0.925176	-0.457960	2.632762
C	2.219750	-3.870647	1.323487
H	2.460578	-2.885278	-0.571228
C	1.820438	-3.743899	2.656642
H	1.041579	-2.408173	4.163930
H	2.590957	-4.822385	0.952660
H	1.880217	-4.597506	3.326719
P	-1.665777	2.365891	-0.100004
O	-0.185021	2.770781	-0.189704
C	-2.686882	3.493627	0.878375
H	-3.738080	3.191652	0.820424
H	-2.353144	3.479033	1.920456
H	-2.578707	4.506515	0.477677
H	-2.222741	2.389392	-1.392152

1a

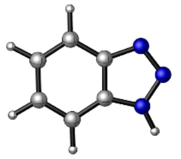

SMD(DCE)-B3LYP-D3/6-31G(d)/LanL2DZ
Thermal correction to Gibbs Free Energy = 0.076546
Sum of electronic and thermal Free Energies = -395.813369
SMD(DCE)-B3LYP-D3/6-311++G(d,p)/SDD
Single point energy = -395.9950225
Corrected quasi-harmonic Free Energy = -395.921708
N_{imag} = 0

H	2.056565	1.903350	0.001164
C	0.273059	-0.721857	0.000293
C	0.277476	0.689383	0.000491
C	-0.904632	1.446384	0.000005
C	-2.089810	0.726626	-0.000210
C	-2.109893	-0.692770	-0.000106
C	-0.939853	-1.434415	0.000186
H	-0.889896	2.531388	-0.000018
H	-3.034165	1.263670	-0.000453
H	-3.068882	-1.202693	-0.000316
H	-0.946667	-2.519931	0.000290
N	1.605316	0.995649	-0.000236
N	2.363753	-0.126240	-0.000245
N	1.580212	-1.163107	-0.000179

1b

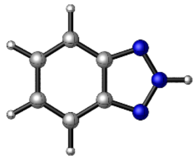

SMD(DCE)-B3LYP-D3/6-31G(d)/LanL2DZ
Thermal correction to Gibbs Free Energy = 0.077033
Sum of electronic and thermal Free Energies = -395.809981
SMD(DCE)-B3LYP-D3/6-311++G(d,p)/SDD
Single point energy = -395.9919928
Corrected quasi-harmonic Free Energy = -395.918162
N_{imag} = 0

H	3.258129	-0.000003	0.000177
C	0.281939	0.713866	0.000041
C	0.281944	-0.713865	-0.000056
C	-0.929463	-1.445237	-0.000115
C	-2.098175	-0.714697	-0.000064
C	-2.098179	0.714691	0.000032
C	-0.929470	1.445236	0.000082
H	-0.927066	-2.530762	-0.000135
H	-3.053002	-1.232983	-0.000075
H	-3.053009	1.232972	0.000028
H	-0.927077	2.530761	0.000074
N	1.567927	-1.140197	0.000137
N	2.242787	0.000002	0.000104
N	1.567921	1.140202	-0.000183

2'

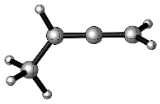

SMD(DCE)-B3LYP-D3/6-31G(d)/LanL2DZ
Thermal correction to Gibbs Free Energy = 0.057352
Sum of electronic and thermal Free Energies = -155.925997
SMD(DCE)-B3LYP-D3/6-311++G(d,p)/SDD
Single point energy = -156.0314544
Corrected quasi-harmonic Free Energy = -155.976515
N_{imag} = 0

C	-0.694622	0.174174	-0.000089
C	-1.944870	-0.213012	0.000013
H	-2.491849	-0.381491	0.927799
H	-2.492246	-0.381384	-0.927544
C	0.557036	0.559051	0.000013
H	0.770893	1.629931	0.000143
C	1.750560	-0.366387	-0.000011
H	2.378655	-0.185894	-0.882312
H	2.378315	-0.186167	0.882568
H	1.447606	-1.417946	-0.000207

INT1

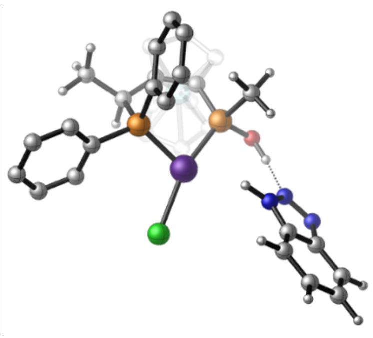

SMD(DCE)-B3LYP-D3/6-31G(d)/LanL2DZ
Thermal correction to Gibbs Free Energy = 0.484638
Sum of electronic and thermal Free Energies = -2815.042578
SMD(DCE)-B3LYP-D3/6-311++G(d,p)/SDD
Single point energy = -2817.5829423
Corrected quasi-harmonic Free Energy = -2817.105002
N_{imag} = 0

Cl	-1.884909	1.385411	-2.484672
P	0.987703	1.527254	-0.015031
C	1.665661	-1.474353	1.096722
C	2.731406	-0.644431	0.568921
C	2.625708	0.629020	-0.254929
H	2.525570	0.342704	-1.308107
C	2.263046	-2.583880	1.784862
C	3.962144	-1.267676	0.949893
C	3.674500	-2.456710	1.684229
H	4.405897	-3.159882	2.061474
H	4.951409	-0.921443	0.685936
H	1.722274	-3.395806	2.252772
C	3.873844	1.522499	-0.141830
H	4.752295	0.975820	-0.500866
H	4.069010	1.841460	0.885860
H	3.770179	2.415024	-0.763589
Fe	2.807157	-2.552911	-0.212265
C	3.262229	-4.370702	-1.107030
C	1.857738	-4.115673	-1.175184
C	1.665367	-2.898605	-1.899855
C	3.938150	-3.308760	-1.783162
C	2.949595	-2.399775	-2.273305
H	5.010683	-3.192083	-1.873624
H	3.141509	-1.470730	-2.795411
H	0.718259	-2.403455	-2.070059
H	1.076797	-4.700814	-0.709364
H	3.735173	-5.196505	-0.590840
C	1.241792	3.046090	-1.017370
C	1.538119	4.288303	-0.435968
C	1.214989	2.929410	-2.416661
C	1.796227	5.398254	-1.244367
H	1.575250	4.394913	0.643127
C	1.484893	4.037813	-3.219536
H	0.965110	1.978161	-2.874961
C	1.772090	5.275367	-2.635369
H	2.019734	6.356727	-0.783547
H	1.461016	3.936691	-4.301164
H	1.974794	6.139751	-3.262048
C	0.890605	2.103095	1.726440
C	-0.252225	2.836641	2.094730
C	1.827743	1.761876	2.712021
C	-0.444588	3.231750	3.417179
H	-0.993646	3.091631	1.341704
C	1.624998	2.148273	4.040859

H	2.704295	1.175025	2.465250
C	0.492856	2.882989	4.396110
H	-1.329367	3.802371	3.685913
H	2.354429	1.868778	4.796120
H	0.337320	3.179807	5.429729
P	-0.097557	-1.175283	0.908715
C	-0.686782	-0.953813	2.638271
H	-0.378959	-1.810216	3.248791
H	-0.278333	-0.035742	3.068253
H	-1.779987	-0.889388	2.637307
O	-0.690626	-2.670571	0.531337
H	-1.665527	-2.567189	0.381902
Rh	-0.724347	0.284671	-0.630303
H	-2.995560	0.072897	0.539475
C	-5.072668	-0.422739	0.190715
C	-5.551425	-1.724754	-0.075822
C	-6.916391	-1.947751	-0.338296
C	-7.748125	-0.841271	-0.327417
C	-7.250659	0.463553	-0.062929
C	-5.911744	0.703024	0.201997
H	-7.287302	-2.946988	-0.542502
H	-8.808215	-0.965054	-0.528118
H	-7.945422	1.298511	-0.070387
H	-5.530618	1.698570	0.403315
N	-4.489518	-2.599900	-0.028104
N	-3.421008	-1.916696	0.248161
N	-3.741875	-0.611607	0.401389

INT2

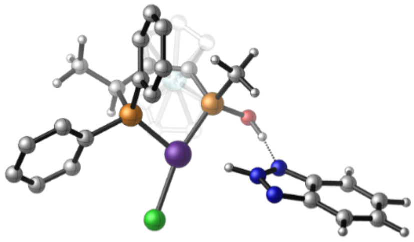

SMD(DCE)-B3LYP-D3/6-31G(d)/LanL2DZ
Thermal correction to Gibbs Free Energy = 0.484469
Sum of electronic and thermal Free Energies = -2815.040520
SMD(DCE)-B3LYP-D3/6-311++G(d,p)/SDD
Single point energy = -2817.5800416
Corrected quasi-harmonic Free Energy = -2817.102043
N_{imag} = 0

Cl	-0.650368	1.718196	-3.042272
P	1.444235	1.280859	0.074116
C	0.915353	-1.761421	1.199036
C	2.265962	-1.322916	0.894170
C	2.736857	-0.083822	0.144954
H	2.789121	-0.337240	-0.919881
C	1.001028	-3.013287	1.898393
C	3.146849	-2.320360	1.419584
C	2.374272	-3.356138	2.023610
H	2.767042	-4.264977	2.461114
H	4.224406	-2.314119	1.334234
H	0.158152	-3.607731	2.225182
C	4.147023	0.376278	0.560084
H	4.873821	-0.408174	0.324095
H	4.222038	0.597689	1.627575
H	4.442019	1.269869	0.003921
Fe	1.851674	-3.145958	0.015271

C	2.317096	-4.910268	-0.973931
C	0.907267	-4.677990	-1.016981
C	0.684905	-3.430665	-1.675405
C	2.965369	-3.804288	-1.605953
C	1.955809	-2.889485	-2.037359
H	4.034575	-3.665710	-1.704711
H	2.121150	-1.931229	-2.513216
H	-0.272502	-2.945535	-1.804796
H	0.144023	-5.314248	-0.587663
H	2.809394	-5.754825	-0.508852
C	2.340507	2.595361	-0.846225
C	2.793083	3.774273	-0.235272
C	2.629106	2.368584	-2.202297
C	3.517769	4.713927	-0.973627
H	2.588184	3.965320	0.812812
C	3.362983	3.303335	-2.931551
H	2.263543	1.470650	-2.689403
C	3.805599	4.480481	-2.319799
H	3.859229	5.626175	-0.491865
H	3.580231	3.117111	-3.979803
H	4.369987	5.212400	-2.891394
C	1.196354	1.946330	1.766244
C	0.257313	2.982976	1.916684
C	1.800296	1.402202	2.908762
C	-0.054230	3.477160	3.181965
H	-0.236610	3.393620	1.039741
C	1.475901	1.891460	4.177841
H	2.497026	0.576652	2.828966
C	0.552287	2.928589	4.317579
H	-0.779016	4.280234	3.283148
H	1.943841	1.453950	5.055472
H	0.299680	3.304529	5.305225
P	-0.594130	-0.887614	0.759386
C	-1.304265	-0.391595	2.384133
H	-1.404530	-1.276196	3.022917
H	-0.663417	0.341085	2.878845
H	-2.293162	0.050791	2.227262
O	-1.626250	-2.093077	0.296587
H	-2.474700	-1.646747	0.041447
Rh	-0.390253	0.552527	-0.904307
H	-2.598568	1.323926	-0.187606
C	-5.660950	0.952893	-0.180149
C	-5.166219	-0.385241	-0.183936
C	-6.042740	-1.494262	-0.181786
C	-7.392947	-1.214225	-0.176419
C	-7.890989	0.125294	-0.173315
C	-7.050156	1.217975	-0.175167
H	-5.662378	-2.510747	-0.184555
H	-8.107780	-2.032258	-0.174645
H	-8.966319	0.278321	-0.169554
H	-7.424784	2.236396	-0.172822
N	-3.813488	-0.325740	-0.184228
N	-3.569918	0.981406	-0.177301
N	-4.600897	1.800318	-0.177644

TS1-3

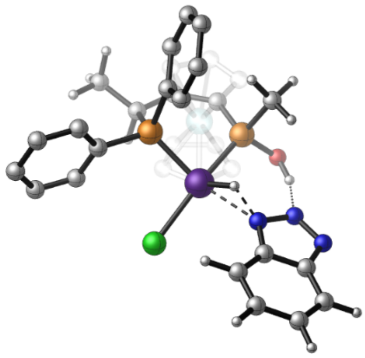

SMD(DCE)-B3LYP-D3/6-31G(d)/LanL2DZ
Thermal correction to Gibbs Free Energy = 0.478414
Sum of electronic and thermal Free Energies = -2815.019695
SMD(DCE)-B3LYP-D3/6-311++G(d,p)/SDD
Single point energy = -2817.5593036
Corrected quasi-harmonic Free Energy = -2817.087729
N_{imag} = 1, ν = -1221.5785i cm^{-1}

Cl	-2.040459	0.864304	-2.321741
P	0.561422	1.602309	0.005916
C	2.001658	-1.148182	1.077041
C	2.781539	-0.137707	0.386302
C	2.316127	1.077354	-0.402274
H	2.180034	0.764752	-1.444437
C	2.911862	-2.116437	1.624037
C	4.155413	-0.510484	0.535357
C	4.232297	-1.722297	1.282638
H	5.137968	-2.269785	1.509807
H	4.998577	0.013245	0.107589
H	2.625496	-3.010547	2.161308
C	3.351318	2.218423	-0.409199
H	4.265763	1.877342	-0.905245
H	3.616928	2.551140	0.597252
H	2.979687	3.080016	-0.968715
Fe	3.077271	-2.001454	-0.437595
C	3.659523	-3.738101	-1.418102
C	2.238605	-3.692330	-1.282381
C	1.769598	-2.519756	-1.953053
C	4.070398	-2.592179	-2.166062
C	2.900827	-1.840978	-2.498239
H	5.090481	-2.320290	-2.405951
H	2.881074	-0.897179	-3.028608
H	0.742830	-2.178228	-1.992161
H	1.625233	-4.380543	-0.717747
H	4.316439	-4.481953	-0.985424
C	0.367854	3.105788	-1.027189
C	0.166930	4.378055	-0.473833
C	0.486059	2.972354	-2.421136
C	0.079503	5.498999	-1.304584
H	0.083802	4.506199	0.599893
C	0.413217	4.094902	-3.243947
H	0.623790	1.991282	-2.862421
C	0.204423	5.361153	-2.687817
H	-0.080056	6.479562	-0.864398
H	0.508150	3.979063	-4.320094
H	0.139264	6.234369	-3.331278
C	0.490487	2.173033	1.745843
C	-0.766692	2.557651	2.246968
C	1.595076	2.155637	2.608774
C	-0.909087	2.936352	3.580510
H	-1.633952	2.551608	1.592235

C	1.445087	2.523001	3.949439
H	2.567680	1.832347	2.258912
C	0.197558	2.916192	4.436527
H	-1.884334	3.236087	3.953733
H	2.306484	2.496267	4.610917
H	0.084463	3.199734	5.479222
P	0.214869	-1.279165	1.192758
C	-0.189110	-1.032203	2.961776
H	0.381202	-1.744191	3.568162
H	0.042953	-0.014097	3.279914
H	-1.259843	-1.215409	3.100935
O	-0.048375	-2.882700	0.978461
H	-1.038124	-2.996573	0.875785
Rh	-0.916843	-0.092389	-0.356378
H	-2.165741	-0.206644	0.673145
C	-4.227077	-1.088919	-0.116117
C	-4.847500	-2.309581	0.231155
C	-6.238616	-2.482950	0.097034
C	-6.965171	-1.403262	-0.375184
C	-6.334781	-0.173924	-0.708931
C	-4.966043	0.009105	-0.589166
H	-6.710712	-3.424595	0.360260
H	-8.041585	-1.490024	-0.493749
H	-6.948390	0.645573	-1.073134
H	-4.478806	0.937975	-0.859378
N	-3.880021	-3.167884	0.698317
N	-2.744594	-2.532392	0.654919
N	-2.893263	-1.277177	0.144309

TS2-4

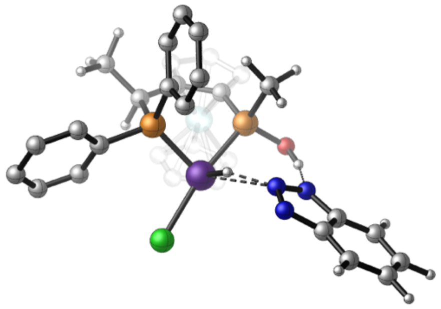

SMD(DCE)-B3LYP-D3/6-31G(d)/LanL2DZ
Thermal correction to Gibbs Free Energy = 0.480088
Sum of electronic and thermal Free Energies = -2815.021704
SMD(DCE)-B3LYP-D3/6-311++G(d,p)/SDD
Single point energy = -2817.5626195
Corrected quasi-harmonic Free Energy = -2817.089528
N_{imag} = 1, ν = -157.2694i cm^{-1}

Cl	-0.659372	1.201282	-3.001432
P	1.195553	1.385660	0.107884
C	1.174003	-1.715919	1.470295
C	2.415996	-1.053789	1.112228
C	2.683033	0.278201	0.422525
H	2.929750	0.040889	-0.618525
C	1.492926	-2.989367	2.051653
C	3.470168	-1.945489	1.484455
C	2.906896	-3.126389	2.051459
H	3.461053	-3.995408	2.381507
H	4.524435	-1.770089	1.319792
H	0.772079	-3.724564	2.383019
C	3.915733	1.005238	1.005412
H	4.822630	0.466820	0.712333

H	3.892329	1.042106	2.096564
H	4.001253	2.025693	0.621300
Fe	2.196249	-2.848050	0.107870
C	2.927933	-4.396578	-1.059279
C	1.505766	-4.492064	-0.955716
C	0.942707	-3.294867	-1.490408
C	3.243564	-3.136934	-1.656788
C	2.016193	-2.455528	-1.922250
H	4.238315	-2.750336	-1.837697
H	1.916897	-1.465416	-2.349259
H	-0.109851	-3.046122	-1.505619
H	0.953348	-5.307940	-0.507838
H	3.642145	-5.129488	-0.706587
C	1.928497	2.650141	-1.000217
C	2.019332	3.997490	-0.622933
C	2.455356	2.241897	-2.237211
C	2.629790	4.922801	-1.474371
H	1.621446	4.331619	0.329108
C	3.070897	3.166589	-3.077772
H	2.369254	1.207731	-2.553673
C	3.156633	4.510498	-2.699161
H	2.692243	5.965297	-1.175028
H	3.472668	2.840159	-4.032930
H	3.630361	5.231888	-3.359322
C	0.642838	2.288786	1.595047
C	-0.560846	3.008970	1.486245
C	1.327029	2.269182	2.818519
C	-1.064450	3.703618	2.584442
H	-1.106476	3.018952	0.547219
C	0.810953	2.959132	3.918622
H	2.242349	1.704303	2.935567
C	-0.381392	3.676562	3.804241
H	-1.995451	4.254778	2.488803
H	1.343170	2.930520	4.865203
H	-0.780605	4.209362	4.662769
P	-0.449175	-1.071216	1.098031
C	-1.104609	-0.369821	2.655270
H	-1.294461	-1.201384	3.342617
H	-0.389828	0.316585	3.110186
H	-2.039122	0.159667	2.453314
O	-1.419805	-2.354241	0.823013
H	-2.319229	-1.940705	0.580736
Rh	-0.398551	0.124073	-0.819893
H	-1.633473	0.842940	-0.158282
C	-5.332827	0.468429	-0.364742
C	-4.895842	-0.855835	-0.092938
C	-5.786595	-1.948794	-0.158512
C	-7.098630	-1.672108	-0.501015
C	-7.538935	-0.345535	-0.774350
C	-6.674995	0.733823	-0.710873
H	-5.452274	-2.960723	0.053004
H	-7.818206	-2.484463	-0.563644
H	-8.580707	-0.184859	-1.039251
H	-7.008442	1.746590	-0.918761
N	-3.572199	-0.782389	0.210935
N	-3.251929	0.523427	0.136470
N	-4.258146	1.300823	-0.216802

INT3

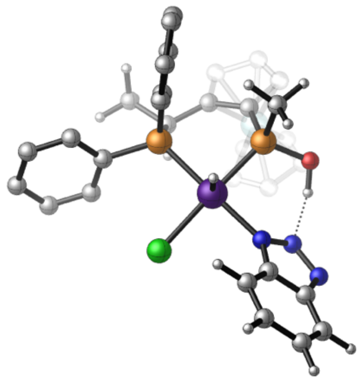

SMD(DCE)-B3LYP-D3/6-31G(d)/LanL2DZ
Thermal correction to Gibbs Free Energy = 0.481625
Sum of electronic and thermal Free Energies = -2815.057892
SMD(DCE)-B3LYP-D3/6-311++G(d,p)/SDD
Single point energy = -2817.604019
Corrected quasi-harmonic Free Energy = -2817.129537
N_{imag} = 0

Cl	-2.020226	2.118354	-1.604726
P	1.097569	1.459719	-0.138445
C	1.465924	-1.610193	1.001153
C	2.416156	-1.040604	0.065069
C	2.273812	0.175242	-0.836722
H	1.722458	-0.127772	-1.735650
C	2.064183	-2.779550	1.586541
C	3.580639	-1.871856	0.110274
C	3.361241	-2.935701	1.033102
H	4.047224	-3.747359	1.237678
H	4.470322	-1.741790	-0.488708
H	1.581712	-3.441670	2.292943
C	3.631349	0.728496	-1.307253
H	4.159613	-0.042733	-1.877124
H	4.267357	1.034258	-0.471927
H	3.496270	1.586266	-1.969095
Fe	1.906388	-2.966253	-0.467027
C	1.913535	-4.852745	-1.328490
C	0.599130	-4.521610	-0.876301
C	0.181755	-3.339089	-1.561160
C	2.311424	-3.871840	-2.288327
C	1.240214	-2.936838	-2.430785
H	3.270623	-3.821601	-2.787368
H	1.245492	-2.055327	-3.059848
H	-0.757457	-2.820971	-1.414979
H	0.033808	-5.046122	-0.117518
H	2.520554	-5.674849	-0.971452
C	1.329573	2.883526	-1.267148
C	1.835380	4.113501	-0.825578
C	1.042009	2.704960	-2.631385
C	2.046113	5.152482	-1.736945
H	2.070911	4.268333	0.221788
C	1.268563	3.738566	-3.537958
H	0.636696	1.762162	-2.983210
C	1.767094	4.967045	-3.091621
H	2.434163	6.103794	-1.383459
H	1.045064	3.588770	-4.590568
H	1.935195	5.774847	-3.798613
C	1.688241	2.007523	1.503342
C	0.864914	2.890623	2.225426
C	2.873654	1.540703	2.088948
C	1.228487	3.304797	3.505996

H	-0.062184	3.250384	1.786936
C	3.226359	1.947511	3.378681
H	3.517536	0.846842	1.562499
C	2.407948	2.828392	4.087818
H	0.586216	3.989386	4.052746
H	4.142055	1.571135	3.826006
H	2.684795	3.140970	5.090837
P	-0.201074	-1.070743	1.412395
C	-0.123953	-0.565652	3.161869
H	0.215254	-1.413457	3.766642
H	0.569871	0.268588	3.286509
H	-1.122976	-0.260289	3.487601
O	-1.074033	-2.440400	1.502890
H	-1.766860	-2.448210	0.772757
Rh	-1.036884	0.476617	-0.057973
H	-1.355002	1.329184	1.157100
C	-4.201859	-0.011151	0.127358
C	-5.041272	-1.140794	-0.043596
C	-6.445347	-1.020262	0.035666
C	-6.959745	0.237197	0.294019
C	-6.110867	1.366977	0.471413
C	-4.733173	1.268083	0.389692
H	-7.086276	-1.887100	-0.096785
H	-8.035344	0.374076	0.366736
H	-6.564551	2.333744	0.673581
H	-4.084369	2.128288	0.507139
N	-4.247691	-2.234637	-0.252357
N	-3.001790	-1.805382	-0.217608
N	-2.928332	-0.476674	0.003927

INT4

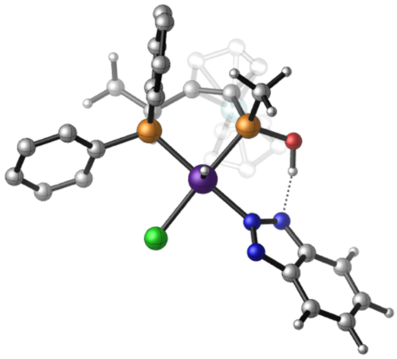

SMD(DCE)-B3LYP-D3/6-31G(d)/LanL2DZ
Thermal correction to Gibbs Free Energy = 0.483264
Sum of electronic and thermal Free Energies = -2815.055957
SMD(DCE)-B3LYP-D3/6-311++G(d,p)/SDD
Single point energy = -2817.6021382
Corrected quasi-harmonic Free Energy = -2817.126541
N_{imag} = 0

Cl	-0.347321	2.877468	-1.700350
P	1.908979	0.687970	-0.120494
C	0.407118	-2.034860	0.953593
C	1.463273	-2.111049	-0.039895
C	2.083588	-1.012324	-0.890267
H	1.459782	-0.884728	-1.783345
C	0.159258	-3.365395	1.439906
C	1.843906	-3.488086	-0.125060
C	1.043111	-4.252278	0.772350
H	1.072197	-5.328048	0.887014
H	2.585252	-3.893909	-0.798441
H	-0.603229	-3.635615	2.157807

C	3.501262	-1.365679	-1.377807
H	3.455834	-2.256581	-2.012354
H	4.187291	-1.569741	-0.551449
H	3.920784	-0.558405	-1.981563
Fe	-0.170031	-3.243867	-0.597469
C	-1.411428	-4.640906	-1.495984
C	-2.185496	-3.565636	-0.961771
C	-1.761782	-2.358081	-1.597869
C	-0.505154	-4.098049	-2.458041
C	-0.722511	-2.687241	-2.519651
H	0.243670	-4.651287	-3.010303
H	-0.167681	-1.984687	-3.129275
H	-2.146951	-1.367830	-1.390927
H	-2.932441	-3.642121	-0.182894
H	-1.467280	-5.677866	-1.190407
C	2.919794	1.749149	-1.219356
C	4.024341	2.472785	-0.749026
C	2.605443	1.784611	-2.589362
C	4.800480	3.224408	-1.635893
H	4.291062	2.452593	0.301935
C	3.391608	2.523870	-3.471116
H	1.746775	1.239839	-2.966346
C	4.488660	3.249628	-2.995760
H	5.652577	3.783436	-1.259053
H	3.141394	2.540549	-4.528202
H	5.096109	3.830868	-3.684147
C	2.734851	0.713152	1.509475
C	2.600807	1.877140	2.287641
C	3.425578	-0.388126	2.036091
C	3.158987	1.940262	3.563487
H	2.052293	2.730829	1.898903
C	3.970127	-0.325173	3.321606
H	3.523931	-1.306100	1.469568
C	3.840104	0.835978	4.085707
H	3.049680	2.845619	4.153672
H	4.492927	-1.188566	3.723452
H	4.262993	0.880044	5.085459
P	-0.554325	-0.614560	1.507973
C	-0.080148	-0.325044	3.244388
H	-0.349699	-1.210051	3.830847
H	0.991712	-0.145882	3.335645
H	-0.630323	0.542968	3.619911
O	-2.064903	-1.182476	1.690358
H	-2.671773	-0.765006	0.995342
Rh	-0.402456	1.110047	0.007256
H	-0.135892	2.025425	1.189566
C	-4.413557	2.374139	0.253124
C	-4.591131	0.975873	0.087943
C	-5.878538	0.402364	0.030104
C	-6.954401	1.266118	0.138909
C	-6.778389	2.670183	0.301441
C	-5.520281	3.242787	0.360326
H	-6.013074	-0.668224	-0.094639
H	-7.964980	0.868248	0.100129
H	-7.659505	3.301327	0.379993
H	-5.381975	4.312963	0.484177
N	-3.349867	0.421609	0.022335
N	-2.494548	1.451817	0.144298
N	-3.073173	2.626995	0.286668

INT5

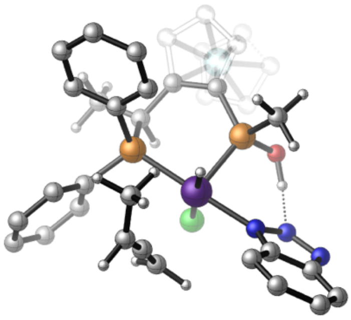

SMD(DCE)-B3LYP-D3/6-31G(d)/LanL2DZ
Thermal correction to Gibbs Free Energy = 0.564300
Sum of electronic and thermal Free Energies = -2970.981122
SMD(DCE)-B3LYP-D3/6-311++G(d,p)/SDD
Single point energy = -2973.6538511
Corrected quasi-harmonic Free Energy = -2973.098280
N_{imag} = 0

C	-3.251201	1.758578	-0.457477
C	-2.975003	1.061161	-1.552118
H	-2.287140	1.443473	-2.301696
H	-3.549156	0.174677	-1.814268
C	-3.628002	2.528074	0.530571
H	-4.419085	3.241793	0.287442
Cl	-0.407677	-0.673412	-2.659532
P	0.312550	1.609198	-0.140220
C	1.938702	-1.067436	0.932443
C	2.565761	0.113821	0.373767
C	2.010306	1.014870	-0.701617
H	1.769444	0.410687	-1.581024
C	2.834373	-1.617540	1.916927
C	3.818229	0.274234	1.042146
C	3.986258	-0.790424	1.974998
H	4.860834	-0.963671	2.588318
H	4.547758	1.043632	0.833483
H	2.664120	-2.517726	2.491579
C	2.991975	2.124120	-1.092598
H	3.916612	1.667234	-1.463737
H	3.247284	2.760770	-0.240222
H	2.586592	2.756518	-1.885020
Fe	3.727272	-1.551516	0.046629
C	5.368553	-2.733307	-0.413589
C	4.161322	-3.480518	-0.581378
C	3.361418	-2.812963	-1.558865
C	5.311972	-1.602700	-1.285179
C	4.069711	-1.652142	-1.991463
H	6.059854	-0.823968	-1.365178
H	3.704080	-0.911512	-2.691241
H	2.359041	-3.090394	-1.852340
H	3.884653	-4.373717	-0.036186
H	6.166405	-2.959785	0.282109
C	-0.167417	2.969689	-1.270810
C	-0.850721	4.100147	-0.794673
C	0.055547	2.836395	-2.652756
C	-1.281187	5.089485	-1.680790
H	-1.052275	4.212728	0.265213
C	-0.374953	3.829610	-3.533437
H	0.539667	1.948890	-3.043958
C	-1.041962	4.958997	-3.050508
H	-1.806797	5.959593	-1.297027
H	-0.191857	3.716080	-4.598350
H	-1.377659	5.729926	-3.738719

C	0.656445	2.389921	1.483892
C	0.363108	1.702901	2.671021
C	1.319187	3.628674	1.560605
C	0.723231	2.238020	3.909287
H	-0.154372	0.751740	2.628044
C	1.672350	4.164455	2.799659
H	1.552697	4.180968	0.656470
C	1.377620	3.469906	3.976224
H	0.487262	1.692653	4.818863
H	2.178960	5.124567	2.844060
H	1.654570	3.888968	4.939719
P	0.272113	-1.662627	0.635114
C	-0.287682	-2.305945	2.249153
H	0.353555	-3.136329	2.562103
H	-0.262618	-1.515122	3.003971
H	-1.314802	-2.665085	2.137890
O	0.385096	-2.975205	-0.310188
H	-0.507987	-3.117575	-0.761908
Rh	-1.197540	-0.179927	-0.242562
H	-1.641291	0.079184	1.227197
C	-3.815287	-1.951629	0.212427
C	-4.234440	-3.261348	-0.130157
C	-5.510048	-3.733931	0.240294
C	-6.323683	-2.873202	0.957349
C	-5.892748	-1.564091	1.309468
C	-4.645244	-1.082557	0.948155
H	-5.831050	-4.736749	-0.026592
H	-7.313527	-3.199761	1.264831
H	-6.564644	-0.927022	1.878852
H	-4.318603	-0.083685	1.213121
N	-3.210823	-3.870450	-0.805867
N	-2.236880	-2.989228	-0.884332
N	-2.556099	-1.821402	-0.296010
C	-3.117607	2.565085	1.944791
H	-2.657978	3.538604	2.159121
H	-3.949791	2.440203	2.648963
H	-2.379454	1.783531	2.128106

INT6

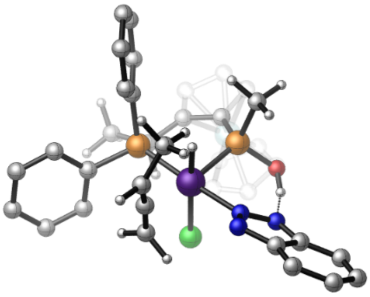

SMD(DCE)-B3LYP-D3/6-31G(d)/LanL2DZ
Thermal correction to Gibbs Free Energy = 0.563444
Sum of electronic and thermal Free Energies = -2970.978638
SMD(DCE)-B3LYP-D3/6-311++G(d,p)/SDD
Single point energy = -2973.6505624
Corrected quasi-harmonic Free Energy = -2973.095645
N_{imag} = 0

C	2.269396	-2.814328	-0.568479
C	1.980190	-2.151344	-1.688443
H	1.092481	-2.365936	-2.271999
H	2.734518	-1.520846	-2.154254
C	2.740388	-3.590151	0.375793
H	3.227521	-4.496268	0.005329

Cl	0.533198	0.702961	-2.410641
P	-1.152789	-1.472219	-0.155502
C	-1.545195	1.550694	1.116676
C	-2.597827	0.874499	0.392416
C	-2.445507	-0.207039	-0.654488
H	-1.951187	0.235855	-1.525673
C	-2.157523	2.522187	1.984835
C	-3.836150	1.434217	0.840542
C	-3.564162	2.444975	1.808887
H	-4.300378	3.074927	2.291060
H	-4.817143	1.166214	0.475220
H	-1.629889	3.207694	2.634479
C	-3.791987	-0.792872	-1.105268
H	-4.416973	0.012569	-1.506809
H	-4.336681	-1.272811	-0.286726
H	-3.655151	-1.527535	-1.901112
Fe	-2.621168	2.900198	0.003556
C	-3.219222	4.792983	-0.594411
C	-1.792268	4.719962	-0.535189
C	-1.364117	3.697649	-1.436225
C	-3.672017	3.812135	-1.529960
C	-2.523958	3.136739	-2.049582
H	-4.704352	3.590596	-1.769306
H	-2.530229	2.307926	-2.745432
H	-0.353556	3.336466	-1.563170
H	-1.150846	5.302773	0.112938
H	-3.849600	5.441739	0.000533
C	-1.383568	-2.817932	-1.385101
C	-1.683525	-4.136811	-1.017064
C	-1.267316	-2.492557	-2.749915
C	-1.856618	-5.117176	-1.998521
H	-1.788474	-4.408228	0.027402
C	-1.448954	-3.474047	-3.723553
H	-1.021731	-1.476396	-3.046150
C	-1.740496	-4.790052	-3.350312
H	-2.086639	-6.136495	-1.700609
H	-1.357771	-3.210338	-4.773615
H	-1.876388	-5.554577	-4.110338
C	-1.601236	-2.190457	1.469953
C	-0.713010	-3.124707	2.031161
C	-2.754935	-1.834637	2.183641
C	-0.971914	-3.690338	3.278172
H	0.176327	-3.414335	1.483752
C	-3.004919	-2.394318	3.440236
H	-3.458037	-1.116492	1.782300
C	-2.117311	-3.320270	3.990309
H	-0.276277	-4.413223	3.695175
H	-3.897981	-2.102589	3.985978
H	-2.315341	-3.752574	4.967246
P	0.221591	1.227515	1.093248
C	0.642443	0.980230	2.854808
H	0.360009	1.859832	3.442852
H	0.114592	0.097907	3.230749
H	1.721286	0.814452	2.936020
O	0.903013	2.630382	0.677587
H	1.833331	2.432813	0.285333
Rh	0.990660	-0.514400	-0.174290
H	1.240236	-1.189657	1.197112
C	5.090427	0.498474	0.010830
C	4.534576	1.802752	-0.042771
C	5.353021	2.951962	-0.027345
C	6.719462	2.745697	0.041859

C	7.280645	1.437328	0.093824
C	6.486633	0.304334	0.079185
H	4.924786	3.949119	-0.069485
H	7.389137	3.601509	0.055986
H	8.361244	1.334478	0.145790
H	6.912167	-0.694335	0.118290
N	3.186440	1.652121	-0.102960
N	2.961551	0.334006	-0.081800
N	4.062007	-0.396409	-0.009732
C	2.790009	-3.394356	1.864806
H	2.331904	-4.242405	2.388858
H	3.839125	-3.348651	2.184778
H	2.295404	-2.471740	2.175716

TS5-7

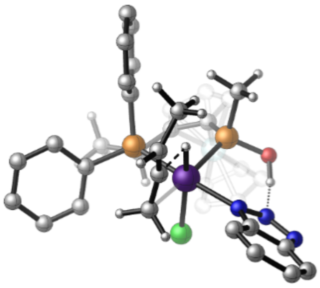

SMD(DCE)-B3LYP-D3/6-31G(d)/LanL2DZ
Thermal correction to Gibbs Free Energy = 0.562943
Sum of electronic and thermal Free Energies = -2970.960388
SMD(DCE)-B3LYP-D3/6-311++G(d,p)/SDD
Single point energy = -2973.635357
Corrected quasi-harmonic Free Energy = -2973.080878
N_{imag} = 1, ν = -662.0878i cm^{-1}

C	-2.769831	1.714393	0.130719
C	-2.625669	1.334953	-1.196969
H	-2.104276	1.981040	-1.895872
H	-3.333976	0.636217	-1.636668
C	-3.426788	2.406543	1.051854
H	-4.212114	3.054787	0.659347
Cl	-0.444001	-1.045873	-2.249120
P	0.639469	1.640920	-0.327128
C	2.061080	-0.940062	1.120907
C	2.773327	-0.135446	0.153027
C	2.192066	0.765941	-0.917660
H	1.772848	0.135128	-1.707076
C	3.034424	-1.619956	1.931993
C	4.170882	-0.328407	0.398904
C	4.327809	-1.241710	1.482970
H	5.268982	-1.618040	1.862881
H	4.977774	0.112750	-0.168187
H	2.810462	-2.325700	2.720957
C	3.238704	1.697049	-1.549754
H	4.038963	1.090244	-1.986884
H	3.684513	2.382171	-0.822000
H	2.801009	2.288475	-2.356593
Fe	3.248499	-2.131654	-0.060496
C	4.226195	-3.878198	-0.603444
C	2.869241	-4.159478	-0.251755
C	2.022313	-3.415647	-1.128811
C	4.216343	-2.958535	-1.697297
C	2.853473	-2.673518	-2.020426
H	5.088157	-2.519603	-2.165854
H	2.501255	-1.978546	-2.770644

H	0.944411	-3.354715	-1.084367
H	2.540023	-4.787143	0.566094
H	5.106642	-4.261894	-0.103564
C	0.356485	2.934234	-1.596142
C	0.453864	4.304319	-1.315323
C	0.075654	2.515476	-2.910075
C	0.267479	5.243064	-2.333989
H	0.678348	4.647487	-0.311517
C	-0.101258	3.457734	-3.922449
H	-0.012618	1.455469	-3.131717
C	-0.008193	4.823791	-3.636531
H	0.342495	6.302542	-2.104806
H	-0.316409	3.124260	-4.933862
H	-0.150186	5.556438	-4.426255
C	1.019428	2.535805	1.229076
C	-0.016939	3.256029	1.848596
C	2.281987	2.503548	1.841761
C	0.199910	3.917018	3.056703
H	-0.992344	3.307597	1.380869
C	2.493506	3.160975	3.056517
H	3.103877	1.961560	1.393565
C	1.455221	3.863830	3.669632
H	-0.613682	4.467830	3.520166
H	3.474892	3.118927	3.520629
H	1.622529	4.370709	4.615976
P	0.279610	-1.082983	1.370985
C	0.065065	-0.528445	3.101658
H	0.696812	-1.117241	3.775645
H	0.336726	0.528968	3.179700
H	-0.985020	-0.654051	3.383465
O	0.003055	-2.680686	1.445175
H	-0.765816	-2.890279	0.805906
Rh	-1.130846	0.089810	-0.127704
H	-1.801542	0.945583	1.073424
C	-4.014675	-1.583978	0.025660
C	-4.303625	-2.972391	-0.050789
C	-5.626607	-3.454599	-0.108189
C	-6.647482	-2.522320	-0.079250
C	-6.364936	-1.133070	0.009366
C	-5.069806	-0.644987	0.062620
H	-5.822946	-4.521348	-0.166295
H	-7.682824	-2.849970	-0.118252
H	-7.193412	-0.429967	0.039816
H	-4.887203	0.416925	0.147920
N	-3.120647	-3.655151	-0.034804
N	-2.174043	-2.748921	0.028303
N	-2.645898	-1.489843	0.068344
C	-3.258388	2.384224	2.539591
H	-3.083166	3.394561	2.929235
H	-4.177711	2.013907	3.012057
H	-2.428428	1.741065	2.849054

TS6-8

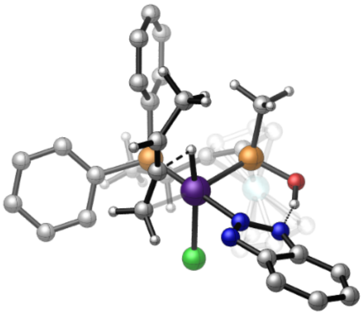

SMD(DCE)-B3LYP-D3/6-31G(d)/LanL2DZ
Thermal correction to Gibbs Free Energy = 0.562057
Sum of electronic and thermal Free Energies = -2970.968155
SMD(DCE)-B3LYP-D3/6-311++G(d,p)/SDD
Single point energy = -2973.641525
Corrected quasi-harmonic Free Energy = -2973.087866
N_{imag} = 1, ν = -620.4714i cm^{-1}

C	1.317240	-3.108484	0.188957
C	1.282939	-2.794808	-1.163529
H	0.439348	-3.109867	-1.769825
H	2.219279	-2.608651	-1.681804
C	1.596023	-3.964524	1.161090
H	1.926002	-4.948187	0.822797
Cl	0.862545	0.454842	-2.288187
P	-1.537501	-1.116500	-0.306175
C	-1.162731	1.836575	1.124684
C	-2.243454	1.573530	0.200013
C	-2.323031	0.496340	-0.863431
H	-1.645907	0.768966	-1.679148
C	-1.541993	2.957580	1.941464
C	-3.267447	2.536546	0.474287
C	-2.833315	3.386084	1.534269
H	-3.375737	4.236569	1.926763
H	-4.200790	2.634329	-0.061229
H	-0.926583	3.413389	2.705846
C	-3.736057	0.333854	-1.444958
H	-4.064289	1.290357	-1.865702
H	-4.464361	0.021123	-0.690174
H	-3.744259	-0.398310	-2.254988
Fe	-1.491884	3.480465	-0.058586
C	-1.313920	5.462522	-0.640691
C	-0.031783	4.927275	-0.302626
C	0.224844	3.817190	-1.163759
C	-1.849187	4.680822	-1.710969
C	-0.897336	3.663971	-2.032240
H	-2.822200	4.810290	-2.167855
H	-1.017460	2.876064	-2.763580
H	1.075760	3.152826	-1.119172
H	0.610411	5.270883	0.497964
H	-1.811151	6.286358	-0.144462
C	-2.095008	-2.318359	-1.574718
C	-3.029996	-3.325752	-1.296275
C	-1.607634	-2.169727	-2.886084
C	-3.464503	-4.178739	-2.314601
H	-3.427011	-3.448990	-0.294533
C	-2.052080	-3.019604	-3.898334
H	-0.879673	-1.394211	-3.107019
C	-2.979098	-4.027857	-3.614790
H	-4.186300	-4.958544	-2.087569
H	-1.670391	-2.896100	-4.908058
H	-3.320295	-4.691768	-4.404379

C	-2.309486	-1.657185	1.266358
C	-1.846449	-2.852886	1.843673
C	-3.300228	-0.918885	1.932152
C	-2.358856	-3.297250	3.061448
H	-1.084047	-3.436204	1.339584
C	-3.803895	-1.363181	3.158048
H	-3.677903	0.006895	1.517282
C	-3.334657	-2.548408	3.726438
H	-1.991433	-4.224216	3.492602
H	-4.564694	-0.776891	3.665790
H	-3.726416	-2.889010	4.680907
P	0.370686	0.913902	1.344043
C	0.234185	0.328018	3.075437
H	0.077856	1.174868	3.752594
H	-0.608607	-0.364400	3.163511
H	1.158581	-0.190543	3.349055
O	1.527545	2.049938	1.407135
H	2.351303	1.682481	0.918219
Rh	0.799446	-0.861863	-0.158720
H	0.862698	-1.923042	1.061204
C	5.037027	-0.755106	-0.253098
C	4.772710	0.589627	0.112708
C	5.814446	1.526426	0.278392
C	7.103631	1.071097	0.065869
C	7.372776	-0.278499	-0.302631
C	6.357899	-1.204712	-0.465700
H	5.607773	2.555668	0.557001
H	7.938450	1.757568	0.179901
H	8.404899	-0.580897	-0.458781
H	6.561338	-2.233912	-0.747387
N	3.425622	0.703784	0.236196
N	2.925409	-0.503068	-0.036875
N	3.843417	-1.408161	-0.332739
C	1.521250	-3.751958	2.641963
H	0.886251	-4.512808	3.112516
H	2.519247	-3.847503	3.089362
H	1.124766	-2.762731	2.893545

INT7

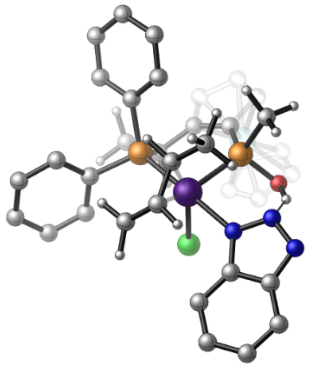

SMD(DCE)-B3LYP-D3/6-31G(d)/LanL2DZ
Thermal correction to Gibbs Free Energy = 0.574116
Sum of electronic and thermal Free Energies = -2971.001614
SMD(DCE)-B3LYP-D3/6-311++G(d,p)/SDD
Single point energy = -2973.6802408
Corrected quasi-harmonic Free Energy = -2973.115727
N_{imag} = 0

C	-2.756589	1.969143	1.075316
C	-2.574094	2.572402	-0.141296
H	-1.714920	3.194544	-0.350624

H	-3.372002	2.581789	-0.876402
C	-1.682002	1.711028	2.006010
H	-0.897489	2.466830	2.027287
Cl	-1.163209	-0.618238	-2.041110
P	0.812349	1.602223	-0.210779
C	1.831839	-1.366729	0.805374
C	2.553276	-0.558115	-0.156929
C	1.979720	0.445636	-1.129164
H	1.295948	-0.074451	-1.805244
C	2.788589	-2.217088	1.467127
C	3.933478	-0.914724	-0.048943
C	4.073936	-1.935155	0.936205
H	4.994078	-2.440843	1.198718
H	4.734204	-0.519147	-0.656647
H	2.559421	-2.961325	2.217572
C	3.068127	1.141269	-1.955336
H	3.601094	0.390091	-2.549166
H	3.799407	1.651876	-1.322578
H	2.640292	1.868260	-2.648881
Fe	2.770997	-2.562077	-0.571023
C	3.356440	-4.429815	-1.262565
C	1.964367	-4.430308	-0.939171
C	1.320537	-3.446167	-1.751727
C	3.574806	-3.440957	-2.270388
C	2.314932	-2.835158	-2.572074
H	4.531299	-3.171203	-2.699739
H	2.149370	-2.017877	-3.262019
H	0.284553	-3.143718	-1.686712
H	1.486633	-5.033577	-0.178739
H	4.119882	-5.036499	-0.792194
C	0.483268	2.984173	-1.371808
C	0.500469	4.325029	-0.954584
C	0.083881	2.680092	-2.685456
C	0.141128	5.343562	-1.841034
H	0.781171	4.581596	0.061423
C	-0.268033	3.702106	-3.566988
H	0.024855	1.646468	-3.009895
C	-0.240181	5.036041	-3.148375
H	0.158228	6.376450	-1.504465
H	-0.569631	3.453765	-4.580872
H	-0.519296	5.829701	-3.835949
C	1.860070	2.309863	1.122201
C	1.673329	1.908770	2.452478
C	2.929522	3.175244	0.821285
C	2.531703	2.351327	3.461084
H	0.854919	1.248904	2.704694
C	3.785529	3.617982	1.829894
H	3.091142	3.514584	-0.195975
C	3.590860	3.205951	3.151623
H	2.367801	2.027952	4.485260
H	4.605574	4.285760	1.581021
H	4.259497	3.552965	3.934539
P	0.087909	-1.298967	1.246901
C	0.106826	-1.443724	3.074966
H	0.593351	-2.388641	3.336629
H	0.651590	-0.621732	3.542343
H	-0.922639	-1.465233	3.436137
O	-0.481831	-2.773101	0.812208
H	-1.436385	-2.778436	1.072717
Rh	-1.184609	0.397253	0.328788
H	-3.710637	1.486006	1.273089
C	-4.085003	-0.865688	-0.045649

C	-4.870918	-1.863456	0.583118
C	-6.180178	-2.146503	0.139039
C	-6.658729	-1.417525	-0.934709
C	-5.859555	-0.428567	-1.574643
C	-4.574142	-0.138839	-1.150829
H	-6.778309	-2.911998	0.625182
H	-7.661170	-1.605707	-1.309896
H	-6.270939	0.104129	-2.428201
H	-3.953065	0.585835	-1.661294
N	-4.128385	-2.418503	1.587626
N	-2.965633	-1.792471	1.591189
N	-2.901223	-0.846756	0.631987
C	-2.035134	1.155281	3.364104
H	-1.145024	0.868673	3.929065
H	-2.560675	1.920398	3.955258
H	-2.691072	0.283286	3.280502

INT8

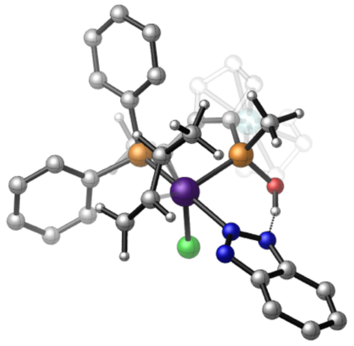

SMD(DCE)-B3LYP-D3/6-31G(d)/LanL2DZ
Thermal correction to Gibbs Free Energy = 0.570340
Sum of electronic and thermal Free Energies = -2971.009460
SMD(DCE)-B3LYP-D3/6-311++G(d,p)/SDD
Single point energy = -2973.6827058
Corrected quasi-harmonic Free Energy = -2973.121037
N_{imag} = 0

C	2.098518	-2.758355	1.005720
C	1.752152	-3.204775	-0.256871
H	0.786670	-3.636914	-0.481286
H	2.534203	-3.361860	-0.995083
C	1.165856	-2.215627	1.945513
H	0.179349	-2.672761	1.986788
Cl	1.233865	-0.186563	-2.301582
P	-1.184484	-1.458042	-0.218442
C	-1.321167	1.652591	0.806010
C	-2.310935	1.025764	-0.039851
C	-2.040263	-0.016458	-1.092534
H	-1.274395	0.350628	-1.780724
C	-2.006178	2.576007	1.669486
C	-3.585432	1.547659	0.340047
C	-3.395262	2.506313	1.378090
H	-4.170138	3.108637	1.834454
H	-4.530704	1.302695	-0.122897
H	-1.539479	3.224612	2.398494
C	-3.299083	-0.386892	-1.883119
H	-3.695747	0.519816	-2.354506
H	-4.081871	-0.802808	-1.242279
H	-3.080509	-1.108145	-2.672463
Fe	-2.324193	3.058636	-0.326296
C	-2.883666	4.982696	-0.869091
C	-1.461272	4.894959	-0.757012

C	-1.005219	3.907794	-1.684023
C	-3.306168	4.044775	-1.860426
C	-2.143638	3.380261	-2.362934
H	-4.329932	3.842067	-2.148458
H	-2.132980	2.578886	-3.090821
H	0.011708	3.556009	-1.781469
H	-0.840879	5.451408	-0.066360
H	-3.532260	5.614240	-0.275290
C	-1.381770	-2.930579	-1.292954
C	-1.696007	-4.192918	-0.761023
C	-1.084036	-2.820179	-2.662351
C	-1.738264	-5.316580	-1.589542
H	-1.900634	-4.308436	0.298200
C	-1.133566	-3.945288	-3.485456
H	-0.787490	-1.863308	-3.077011
C	-1.462588	-5.195231	-2.953095
H	-1.984295	-6.285746	-1.164501
H	-0.906460	-3.844671	-4.543145
H	-1.496452	-6.070285	-3.596267
C	-2.303901	-1.736676	1.216557
C	-1.976327	-1.218759	2.478606
C	-3.569194	-2.326654	1.037450
C	-2.882614	-1.289927	3.537781
H	-1.014557	-0.751063	2.639819
C	-4.472900	-2.399821	2.098402
H	-3.854004	-2.734451	0.073953
C	-4.132946	-1.882470	3.351086
H	-2.607019	-0.882525	4.506506
H	-5.443888	-2.861849	1.942939
H	-4.839007	-1.940250	4.174932
P	0.431257	1.235665	0.883895
C	0.886205	1.600582	2.619889
H	0.761974	2.672263	2.807182
H	0.265546	1.037483	3.320648
H	1.935920	1.331503	2.766904
O	1.187250	2.394755	0.039220
H	2.125763	2.033861	-0.162891
Rh	1.096232	-0.898328	0.148049
H	3.147525	-2.540885	1.181051
C	5.318428	-0.054682	0.187626
C	4.794253	1.225221	-0.122100
C	5.639126	2.321013	-0.398852
C	7.001518	2.085485	-0.353121
C	7.531967	0.800216	-0.041443
C	6.711371	-0.279686	0.231501
H	5.233054	3.299372	-0.638795
H	7.691997	2.898487	-0.561319
H	8.610916	0.672242	-0.020589
H	7.114230	-1.260229	0.468461
N	3.443714	1.113817	-0.083475
N	3.176600	-0.154883	0.235258
N	4.266615	-0.891782	0.404887
C	1.679203	-1.713535	3.272148
H	0.944195	-1.084077	3.779801
H	1.893058	-2.562445	3.938790
H	2.604138	-1.139587	3.152699

INT9

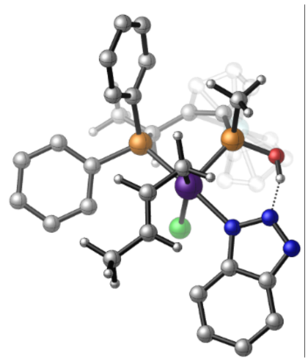

SMD(DCE)-B3LYP-D3/6-31G(d)/LanL2DZ
Thermal correction to Gibbs Free Energy = 0.572142
Sum of electronic and thermal Free Energies = -2971.019410
SMD(DCE)-B3LYP-D3/6-311++G(d,p)/SDD
Single point energy = -2973.6946447
Corrected quasi-harmonic Free Energy = -2973.131468
N_{imag} = 0

C	-2.431312	2.089000	1.207815
C	-1.616282	1.196140	2.033242
H	-2.172722	0.510138	2.675038
H	-0.810947	1.692345	2.572387
C	-3.676022	1.806836	0.751017
H	-4.167258	0.900339	1.098283
H	-1.963130	3.004295	0.848680
Cl	-0.980981	-0.863018	-2.026640
P	0.695508	1.626278	-0.287992
C	2.226866	-1.052676	0.783605
C	2.742424	-0.308717	-0.347936
C	2.028078	0.700832	-1.232005
H	1.413165	0.156924	-1.955468
C	3.300476	-1.844802	1.325037
C	4.123311	-0.663928	-0.473201
C	4.461266	-1.603107	0.544933
H	5.422522	-2.084734	0.669436
H	4.795980	-0.306202	-1.239398
H	3.215985	-2.527712	2.159732
C	2.996732	1.608236	-2.002475
H	3.632293	0.996776	-2.651844
H	3.643489	2.186073	-1.334873
H	2.450712	2.302115	-2.646202
Fe	2.916898	-2.355760	-0.635599
C	1.333080	-3.525408	-1.275484
C	1.933011	-2.860369	-2.386772
C	3.302064	-3.263025	-2.458004
C	2.329745	-4.341692	-0.658651
C	3.548102	-4.179188	-1.388874
H	2.197786	-4.938517	0.234647
H	4.500061	-4.635734	-1.148635
H	4.036443	-2.902545	-3.167107
H	1.436554	-2.136396	-3.017744
H	0.322149	-3.371792	-0.925122
C	0.030555	2.893794	-1.441717
C	0.704485	4.113090	-1.633906
C	-1.160262	2.652449	-2.144375
C	0.194380	5.068784	-2.513194
H	1.623763	4.320319	-1.095939
C	-1.668260	3.613595	-3.021092
H	-1.680197	1.710091	-2.019637
C	-0.994266	4.822489	-3.206532
H	0.725720	6.005963	-2.654295

H	-2.591757	3.413258	-3.557404
H	-1.391853	5.569671	-3.888018
C	1.525478	2.623519	1.016609
C	0.814893	3.677312	1.620665
C	2.821476	2.337039	1.476488
C	1.378540	4.412324	2.663159
H	-0.175866	3.941097	1.267336
C	3.384392	3.077785	2.518740
H	3.401572	1.537678	1.033989
C	2.664890	4.112027	3.119384
H	0.812052	5.221758	3.115061
H	4.389630	2.841643	2.856631
H	3.104062	4.684158	3.931871
P	0.609315	-0.974504	1.558759
C	0.961975	-0.285783	3.216047
H	1.729615	-0.906832	3.689323
H	1.325088	0.741847	3.140397
H	0.052582	-0.310969	3.822958
O	0.238791	-2.520909	1.861386
H	-0.767735	-2.578765	2.007903
Rh	-0.963122	0.085104	0.368470
C	-4.291043	-2.636663	0.923894
C	-3.668255	-1.594074	0.193080
C	-4.304931	-0.995062	-0.913720
C	-5.563852	-1.464507	-1.244210
C	-6.195909	-2.506574	-0.508306
C	-5.575881	-3.104348	0.574057
H	-3.815264	-0.215836	-1.485108
H	-6.086259	-1.032412	-2.093745
H	-7.184951	-2.837974	-0.813301
H	-6.048812	-3.903889	1.137039
N	-2.464339	-1.380688	0.798359
N	-2.374409	-2.256553	1.814941
N	-3.438729	-3.025146	1.919924
C	-4.449395	2.663872	-0.200094
H	-3.877745	3.541541	-0.520069
H	-4.733108	2.089607	-1.093102
H	-5.389432	3.006252	0.255072

INT10

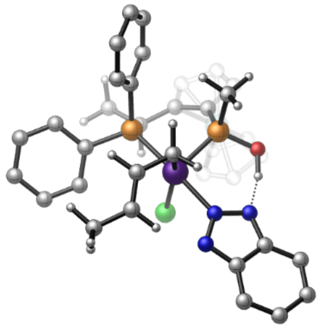

SMD(DCE)-B3LYP-D3/6-31G(d)/LanL2DZ
Thermal correction to Gibbs Free Energy = 0.568971
Sum of electronic and thermal Free Energies = -2971.023986
SMD(DCE)-B3LYP-D3/6-311++G(d,p)/SDD
Single point energy = -2973.6946429
Corrected quasi-harmonic Free Energy = -2973.134382
N_{imag} = 0

C	0.205290	-3.406589	0.941923
C	0.468093	-2.240029	1.793673
H	1.453186	-2.213250	2.263931
H	-0.322903	-2.023489	2.510503

C	1.130621	-4.070437	0.206294
H	2.174805	-3.780603	0.293994
H	-0.835757	-3.696753	0.806151
Cl	1.060185	-0.021742	-2.206578
P	-1.750178	-0.805752	-0.201486
C	-0.890461	2.136050	0.824177
C	-1.863771	1.993278	-0.238158
C	-2.126863	0.784784	-1.117555
H	-1.362833	0.746812	-1.900791
C	-1.072623	3.433649	1.421556
C	-2.619716	3.208201	-0.270455
C	-2.135060	4.086813	0.742681
H	-2.482626	5.095227	0.926442
H	-3.410722	3.435332	-0.971082
H	-0.473154	3.845463	2.222425
C	-3.508657	0.810118	-1.785609
H	-3.588291	1.694212	-2.427397
H	-4.322883	0.840829	-1.054965
H	-3.646847	-0.068345	-2.421277
Fe	-0.598260	3.605363	-0.577690
C	1.210639	4.606390	-0.701445
C	1.287268	3.363446	-1.400228
C	0.299955	3.374620	-2.431299
C	0.174985	5.388047	-1.301886
C	-0.387480	4.625811	-2.372029
H	-0.156748	6.365394	-0.974562
H	-1.218005	4.927079	-2.997954
H	0.083767	2.548302	-3.093450
H	1.923049	2.525439	-1.153112
H	1.799297	4.885561	0.162858
C	-2.287047	-2.162860	-1.314777
C	-3.586494	-2.694061	-1.247932
C	-1.386333	-2.665306	-2.268341
C	-3.971332	-3.719042	-2.113717
H	-4.298154	-2.313916	-0.522649
C	-1.779130	-3.688027	-3.133675
H	-0.385671	-2.253007	-2.339126
C	-3.068263	-4.220470	-3.055124
H	-4.977561	-4.124208	-2.051062
H	-1.072461	-4.070469	-3.864908
H	-3.369185	-5.021391	-3.724987
C	-2.884883	-0.908724	1.245333
C	-2.989055	-2.122548	1.950376
C	-3.612142	0.199344	1.712470
C	-3.782077	-2.219893	3.093386
H	-2.466881	-3.005530	1.601889
C	-4.406453	0.098319	2.857211
H	-3.566214	1.149558	1.197950
C	-4.490097	-1.107231	3.554949
H	-3.847384	-3.168054	3.619656
H	-4.960472	0.968486	3.198184
H	-5.105394	-1.182282	4.447174
P	0.318192	0.954975	1.454192
C	-0.233625	0.646550	3.169458
H	-0.316087	1.606408	3.690413
H	-1.204300	0.145397	3.183041
H	0.508075	0.023607	3.677297
O	1.667806	1.806879	1.649647
H	2.490933	1.271812	1.289439
Rh	0.577097	-0.887096	0.185209
C	4.762850	-1.469665	0.105269
C	4.733680	-0.141693	0.601857

C	5.921407	0.574973	0.856203
C	7.113059	-0.079262	0.596544
C	7.145949	-1.412308	0.096620
C	5.985664	-2.122977	-0.155651
H	5.896618	1.591902	1.236502
H	8.053761	0.434279	0.776881
H	8.110506	-1.876997	-0.089530
H	6.007060	-3.139829	-0.536836
N	3.424231	0.186748	0.749026
N	2.722459	-0.882887	0.365007
N	3.473071	-1.893731	-0.028791
C	0.811703	-5.174776	-0.751340
H	-0.262412	-5.382509	-0.801743
H	1.162076	-4.921938	-1.762093
H	1.333470	-6.100302	-0.468887

TS9-11

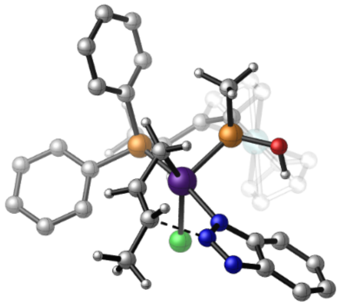

SMD(DCE)-B3LYP-D3/6-31G(d)/LanL2DZ
Thermal correction to Gibbs Free Energy = 0.569568
Sum of electronic and thermal Free Energies = -2970.977093
SMD(DCE)-B3LYP-D3/6-311++G(d,p)/SDD
Single point energy = -2973.6485978
Corrected quasi-harmonic Free Energy = -2973.088023
N_{imag} = 1, ν = -292.3342i cm^{-1}

C	0.872032	-3.381078	-0.821511
C	0.825265	-2.345906	-1.805120
H	0.126011	-2.500605	-2.629043
H	1.735696	-1.843637	-2.119238
C	-0.060669	-4.443936	-0.841166
H	-0.400910	-4.791716	-1.813105
H	1.624488	-3.377286	-0.034745
Cl	-0.841945	-0.854185	2.296220
P	1.877592	-0.020643	0.287911
C	-0.220205	2.188143	-0.980316
C	0.679249	2.533106	0.101293
C	1.386041	1.611311	1.083262
H	0.652305	1.249960	1.812043
C	-0.618072	3.406100	-1.633277
C	0.820666	3.958116	0.077516
C	0.023435	4.489604	-0.978633
H	-0.111559	5.540299	-1.201709
H	1.398119	4.545498	0.777003
H	-1.316999	3.472360	-2.456497
C	2.516852	2.317668	1.842611
H	2.109143	3.170868	2.395319
H	3.298500	2.686330	1.170195
H	2.973044	1.647225	2.574238
Fe	-1.170560	3.436129	0.362469
C	-3.164655	2.958566	0.695536
C	-2.352961	2.460596	1.762860
C	-1.691468	3.571869	2.364615

C	-3.002954	4.375625	0.639886
C	-2.090387	4.756346	1.670937
H	-3.452435	5.039001	-0.088086
H	-1.729688	5.759068	1.861958
H	-0.973254	3.518393	3.172984
H	-2.203739	1.422184	2.030742
H	-3.764395	2.363045	0.019728
C	2.934093	-0.902494	1.517595
C	4.279313	-0.529401	1.697749
C	2.417589	-1.966142	2.272760
C	5.084321	-1.205977	2.614016
H	4.703769	0.285555	1.120488
C	3.229422	-2.645124	3.185473
H	1.378901	-2.250813	2.160757
C	4.562323	-2.268570	3.357930
H	6.120527	-0.905112	2.742615
H	2.813837	-3.468048	3.760780
H	5.192792	-2.798490	4.066943
C	3.089300	0.341032	-1.050376
C	3.879385	-0.719819	-1.533031
C	3.229080	1.606761	-1.641181
C	4.773150	-0.521419	-2.585129
H	3.810654	-1.702268	-1.074858
C	4.128835	1.802968	-2.692627
H	2.643377	2.446959	-1.290710
C	4.898630	0.741485	-3.171028
H	5.374090	-1.353109	-2.942406
H	4.225900	2.791387	-3.133459
H	5.595809	0.896768	-3.989754
P	-0.656893	0.537487	-1.567444
C	0.089321	0.508144	-3.238523
H	-0.240718	1.383048	-3.808210
H	1.178714	0.522567	-3.153954
H	-0.218674	-0.403583	-3.757680
O	-2.231326	0.657371	-2.060484
H	-2.854571	0.470122	-1.334361
Rh	-0.074396	-1.193782	-0.239261
C	-3.950276	-3.103474	-0.383254
C	-3.187844	-1.929074	-0.163281
C	-3.794760	-0.727905	0.254118
C	-5.175486	-0.725144	0.364201
C	-5.948897	-1.887941	0.094360
C	-5.355300	-3.086978	-0.262862
H	-3.193503	0.121516	0.557140
H	-5.681377	0.178498	0.693072
H	-7.029006	-1.834173	0.198022
H	-5.940245	-3.985217	-0.437231
N	-1.886698	-2.270791	-0.377937
N	-1.889489	-3.577993	-0.644127
N	-3.079676	-4.121591	-0.672419
C	-0.094403	-5.469151	0.253956
H	0.090756	-5.011350	1.231064
H	-1.055519	-5.990342	0.279203
H	0.686983	-6.219357	0.069635

TS10-12

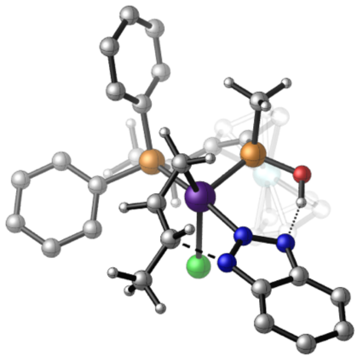

SMD(DCE)-B3LYP-D3/6-31G(d)/LanL2DZ
Thermal correction to Gibbs Free Energy = 0.570680
Sum of electronic and thermal Free Energies = -2970.990495
SMD(DCE)-B3LYP-D3/6-311++G(d,p)/SDD
Single point energy = -2973.6613532
Corrected quasi-harmonic Free Energy = -2973.099676
N_{imag} = 1, ν = -359.6732i cm^{-1}

C	-1.701225	2.818221	0.619984
C	-1.089671	2.167221	1.742587
H	-1.761705	1.797461	2.519702
H	-0.149639	2.537923	2.142669
C	-3.111269	2.834622	0.425228
H	-3.729192	2.840335	1.320555
H	-1.099160	3.378668	-0.092848
Cl	-1.057336	-0.248509	-2.215768
P	1.459184	1.277221	-0.199599
C	1.575368	-1.837607	0.902732
C	2.461004	-1.380026	-0.148777
C	2.326945	-0.159474	-1.047650
H	1.593319	-0.383025	-1.829785
C	2.149903	-3.019219	1.486343
C	3.560518	-2.297642	-0.185264
C	3.366059	-3.301094	0.809348
H	4.011260	-4.152422	0.984737
H	4.386762	-2.262287	-0.880785
H	1.703312	-3.608335	2.276513
C	3.656032	0.223523	-1.714373
H	4.016668	-0.611048	-2.325074
H	4.427401	0.467875	-0.976404
H	3.528209	1.079843	-2.380605
Fe	1.763498	-3.295585	-0.521251
C	0.337897	-4.786019	-0.676775
C	-0.106360	-3.607978	-1.350915
C	0.839160	-3.301056	-2.374822
C	1.559895	-5.210013	-1.287346
C	1.869992	-4.290241	-2.337009
H	2.167484	-6.051685	-0.979266
H	2.752682	-4.315618	-2.963614
H	0.795428	-2.432696	-3.017588
H	-0.961248	-2.999851	-1.092265
H	-0.139775	-5.246447	0.178317
C	1.583397	2.678214	-1.391335
C	2.710694	3.518665	-1.419676
C	0.546167	2.896790	-2.312352
C	2.790130	4.563167	-2.341595
H	3.528092	3.362636	-0.723600
C	0.632430	3.941711	-3.235582
H	-0.315503	2.238218	-2.316902
C	1.749946	4.779079	-3.250051

H	3.666053	5.206263	-2.349397
H	-0.177418	4.098369	-3.943088
H	1.812802	5.594109	-3.966198
C	2.503657	1.822842	1.212659
C	2.276699	3.097734	1.764867
C	3.462855	0.990515	1.811762
C	2.978937	3.519842	2.893951
H	1.563303	3.772875	1.301556
C	4.166147	1.417616	2.941331
H	3.667375	0.007138	1.407251
C	3.922701	2.678175	3.489514
H	2.790030	4.507733	3.304936
H	4.905636	0.759426	3.389236
H	4.468584	3.006123	4.369833
P	0.052279	-1.060575	1.475179
C	0.443470	-0.640364	3.215151
H	0.779045	-1.531439	3.757334
H	1.232984	0.116512	3.240805
H	-0.454292	-0.238691	3.695458
O	-1.007486	-2.293844	1.647228
H	-1.896228	-2.038248	1.247305
Rh	-0.738234	0.679402	0.261010
C	-4.892185	0.296794	0.179466
C	-4.571716	-1.046491	0.500499
C	-5.575350	-2.028155	0.614991
C	-6.880209	-1.617503	0.402342
C	-7.198833	-0.268491	0.083214
C	-6.222142	0.706208	-0.034935
H	-5.327635	-3.057242	0.856691
H	-7.687070	-2.341081	0.477995
H	-8.240003	-0.000798	-0.074085
H	-6.466185	1.734691	-0.280605
N	-3.216341	-1.117290	0.657106
N	-2.752463	0.098840	0.444172
N	-3.703612	0.967721	0.135301
C	-3.693753	3.555362	-0.756710
H	-3.114191	3.358360	-1.664100
H	-4.735602	3.274061	-0.932110
H	-3.666198	4.635643	-0.559840

INT11

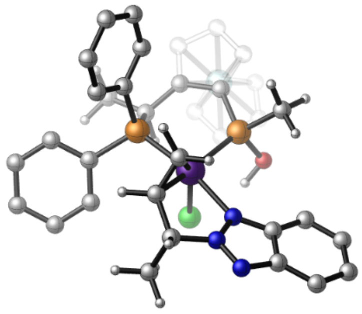

SMD(DCE)-B3LYP-D3/6-31G(d)/LanL2DZ
Thermal correction to Gibbs Free Energy = 0.574715
Sum of electronic and thermal Free Energies = -2971.015920
SMD(DCE)-B3LYP-D3/6-311++G(d,p)/SDD
Single point energy = -2973.6883591
Corrected quasi-harmonic Free Energy = -2973.122161
N_{imag} = 0

C	-2.553836	-1.988818	-0.423292
C	-2.102762	-1.513888	-1.691647
H	-2.753599	-0.886874	-2.302867

H	-1.403241	-2.107814	-2.271638
C	-3.923445	-1.631641	0.110149
H	-4.721782	-2.137114	-0.446016
H	-2.189490	-2.943452	-0.052211
Cl	-0.797429	0.738668	2.453680
P	0.656947	-1.666538	0.002774
C	1.901202	1.347413	-0.961572
C	2.672204	0.300421	-0.328126
C	2.168423	-0.738343	0.647805
H	1.717531	-0.219058	1.501948
C	2.802125	2.118568	-1.773959
C	4.025377	0.438162	-0.778153
C	4.102686	1.555252	-1.660933
H	5.004706	1.938542	-2.120449
H	4.860942	-0.173315	-0.468307
H	2.537364	2.995186	-2.349975
C	3.296447	-1.644588	1.155670
H	4.100367	-1.024068	1.566320
H	3.716095	-2.260469	0.352863
H	2.948637	-2.303013	1.953396
Fe	3.399959	2.148984	0.211488
C	2.798895	3.869239	1.195494
C	2.666150	2.732260	2.052172
C	3.957905	2.145100	2.207075
C	4.171841	3.982846	0.821145
C	4.889143	2.913373	1.440936
H	4.588518	4.718008	0.144231
H	5.943732	2.699804	1.320979
H	4.182507	1.244617	2.764634
H	1.738473	2.340082	2.449129
H	1.983645	4.485570	0.842871
C	0.425403	-3.005166	1.256641
C	1.199489	-4.179396	1.235817
C	-0.516296	-2.831571	2.281669
C	1.022750	-5.161310	2.211470
H	1.939067	-4.332276	0.456856
C	-0.689008	-3.814889	3.258764
H	-1.106205	-1.923692	2.315733
C	0.075581	-4.982800	3.224190
H	1.624996	-6.065260	2.179088
H	-1.424653	-3.665127	4.044559
H	-0.062968	-5.749748	3.981642
C	1.127174	-2.578730	-1.528484
C	0.476174	-3.788563	-1.834407
C	2.018089	-2.039218	-2.470926
C	0.727150	-4.449774	-3.037451
H	-0.229838	-4.220088	-1.131974
C	2.270876	-2.706640	-3.671816
H	2.518771	-1.099026	-2.279692
C	1.629075	-3.912973	-3.959431
H	0.214486	-5.383579	-3.252096
H	2.971817	-2.277407	-4.382754
H	1.826363	-4.429083	-4.895097
P	0.106220	1.569840	-0.794879
C	-0.325139	2.401191	-2.370207
H	0.174505	3.371885	-2.460146
H	-0.028286	1.759548	-3.206213
H	-1.408009	2.548063	-2.410365
O	-0.100671	2.832336	0.266130
H	-0.351753	2.406693	1.125778
Rh	-1.197250	-0.319688	-0.186126
C	-5.150204	1.655362	-0.281254

C	-3.745310	1.892518	-0.250103
C	-3.232374	3.208447	-0.294818
C	-4.154833	4.231367	-0.391127
C	-5.560011	3.992671	-0.442072
C	-6.077490	2.716113	-0.385437
H	-2.169799	3.402461	-0.226967
H	-3.801473	5.258287	-0.420790
H	-6.232427	4.842308	-0.517835
H	-7.144436	2.519583	-0.410679
N	-3.139136	0.676858	-0.161257
N	-4.160217	-0.191718	-0.131368
N	-5.368290	0.320172	-0.198107
C	-4.083417	-1.909911	1.608494
H	-3.364839	-1.316332	2.181910
H	-5.096903	-1.665175	1.941008
H	-3.898039	-2.971545	1.801541

INT12

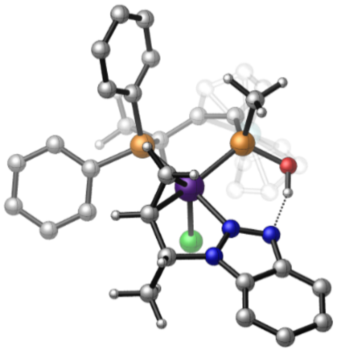

SMD(DCE)-B3LYP-D3/6-31G(d)/LanL2DZ
Thermal correction to Gibbs Free Energy = 0.574730
Sum of electronic and thermal Free Energies = -2971.017549
SMD(DCE)-B3LYP-D3/6-311++G(d,p)/SDD
Single point energy = -2973.6912441
Corrected quasi-harmonic Free Energy = -2973.125472
N_{imag} = 0

C	2.235763	-2.245973	0.164327
C	1.775326	-2.020957	1.497043
H	2.437913	-1.558870	2.231060
H	1.050970	-2.699271	1.937924
C	3.627571	-1.819535	-0.278893
H	4.405288	-2.399561	0.235677
H	1.856168	-3.096210	-0.396826
Cl	0.884451	0.543490	-2.252086
P	-1.123900	-1.530436	-0.078195
C	-1.858727	1.611803	0.949525
C	-2.692926	0.868836	0.025580
C	-2.312064	-0.296777	-0.874608
H	-1.658330	0.086962	-1.665019
C	-2.673100	2.621009	1.568211
C	-4.003718	1.444038	0.101809
C	-3.987516	2.519944	1.038532
H	-4.822639	3.168012	1.272156
H	-4.859818	1.145638	-0.485953
H	-2.325359	3.352321	2.286085
C	-3.539779	-0.950630	-1.522293
H	-4.092215	-0.203259	-2.102280
H	-4.218365	-1.376994	-0.775327
H	-3.244291	-1.742756	-2.213532
Fe	-2.611067	2.873954	-0.483024
C	-1.737628	4.709364	-0.872049

C	-1.005711	3.666686	-1.519048
C	-1.889680	2.999034	-2.418882
C	-3.076427	4.686849	-1.374908
C	-3.169720	3.628505	-2.331350
H	-3.888065	5.328566	-1.055600
H	-4.063496	3.333297	-2.866568
H	-1.632974	2.131486	-3.011551
H	0.012157	3.370007	-1.310359
H	-1.358081	5.368716	-0.102057
C	-1.083624	-2.931904	-1.278292
C	-1.986021	-4.007479	-1.208069
C	-0.144246	-2.898764	-2.322193
C	-1.935379	-5.035936	-2.150924
H	-2.729274	-4.050374	-0.418848
C	-0.101764	-3.926556	-3.266890
H	0.544722	-2.063928	-2.396487
C	-0.992395	-4.999346	-3.181692
H	-2.636449	-5.863356	-2.080092
H	0.631568	-3.886709	-4.068189
H	-0.953979	-5.801439	-3.914133
C	-1.938689	-2.248200	1.407888
C	-1.448112	-3.459884	1.930246
C	-2.976694	-1.593948	2.090411
C	-1.972826	-3.993544	3.107318
H	-0.668102	-4.002113	1.404756
C	-3.501156	-2.131882	3.269261
H	-3.384043	-0.664877	1.712517
C	-2.998889	-3.327964	3.784302
H	-1.581775	-4.931497	3.492194
H	-4.306442	-1.611314	3.780614
H	-3.407236	-3.743187	4.701662
P	-0.146615	1.223552	1.368371
C	-0.277676	0.960403	3.184354
H	-0.679680	1.847773	3.686561
H	-0.933982	0.105703	3.381169
H	0.718622	0.738118	3.581277
O	0.638479	2.680738	1.302285
H	1.564853	2.519603	0.995722
Rh	0.920545	-0.516393	0.241371
C	4.999317	0.321514	0.238080
C	4.549295	1.623988	0.553816
C	5.463713	2.677929	0.732803
C	6.806415	2.375045	0.581770
C	7.245985	1.062447	0.262000
C	6.359452	0.012023	0.083019
H	5.120590	3.678002	0.976812
H	7.548363	3.157677	0.708954
H	8.311026	0.879405	0.153843
H	6.691508	-0.990342	-0.163581
N	3.179603	1.597816	0.634006
N	2.800623	0.379098	0.382051
N	3.856178	-0.419001	0.147313
C	3.821406	-1.922385	-1.794580
H	3.097129	-1.279623	-2.304878
H	4.834968	-1.625227	-2.083948
H	3.662738	-2.959390	-2.109636

3’

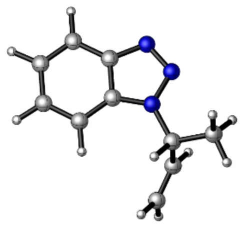

SMD(DCE)-B3LYP-D3/6-31G(d)/LanL2DZ
Thermal correction to Gibbs Free Energy = 0.158735
Sum of electronic and thermal Free Energies = -551.766747
SMD(DCE)-B3LYP-D3/6-311++G(d,p)/SDD
Single point energy = -552.0701745
Corrected quasi-harmonic Free Energy = -551.91535
N_{imag} = 0

C	2.431744	-0.705739	-0.765173
C	2.712165	-2.005557	-0.849272
H	2.584078	-2.674451	0.000315
H	3.088185	-2.449052	-1.768088
C	1.927490	-0.031862	0.489709
H	1.766174	-0.784849	1.267686
H	2.562583	-0.045958	-1.623168
C	-0.609002	-0.090012	0.209554
C	-1.515839	0.904173	-0.210218
C	-2.886840	0.615936	-0.338750
C	-3.292775	-0.675397	-0.043545
C	-2.367151	-1.668639	0.371370
C	-1.013139	-1.401487	0.505809
H	-3.588153	1.380403	-0.658544
H	-4.342291	-0.941485	-0.130978
H	-2.733094	-2.668544	0.587346
H	-0.304869	-2.162512	0.815305
N	-0.810975	2.067304	-0.432381
N	0.444370	1.838053	-0.175797
N	0.597294	0.552167	0.227178
C	2.895217	1.034298	1.008213
H	3.106067	1.786936	0.243476
H	2.483985	1.537330	1.889317
H	3.835446	0.548374	1.286083

4’

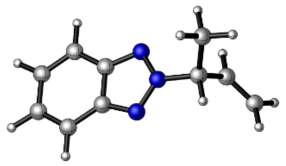

SMD(DCE)-B3LYP-D3/6-31G(d)/LanL2DZ
Thermal correction to Gibbs Free Energy = 0.158075
Sum of electronic and thermal Free Energies = -551.766533
SMD(DCE)-B3LYP-D3/6-311++G(d,p)/SDD
Single point energy = -552.0693577
Corrected quasi-harmonic Free Energy = -551.914855
N_{imag} = 0

C	-2.925389	-0.055193	0.871309
C	-3.742098	-1.026210	1.277209
H	-3.960965	-1.889647	0.651244
H	-4.227577	-0.990044	2.249505
C	-2.259772	-0.035192	-0.483845
H	-2.463248	-0.965826	-1.017420
H	-2.713971	0.803490	1.507434
C	1.133845	0.630586	0.216694

C	1.203046	-0.689130	-0.317736
C	2.438874	-1.364465	-0.441250
C	3.564799	-0.686193	-0.022281
C	3.495302	0.635779	0.512716
C	2.298582	1.310209	0.640849
H	2.489568	-2.369128	-0.849228
H	4.536953	-1.165371	-0.098197
H	4.417122	1.118570	0.825150
H	2.244299	2.315805	1.046010
N	-0.053697	-1.080219	-0.643653
N	-0.789424	-0.025904	-0.311992
N	-0.163317	1.023340	0.206011
C	-2.687102	1.168638	-1.329030
H	-2.478579	2.106624	-0.805923
H	-2.156442	1.174245	-2.287046
H	-3.762622	1.107777	-1.5218962

INT1*

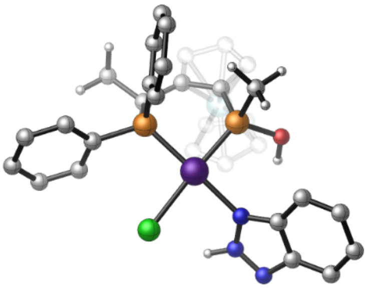

SMD(DCE)-B3LYP-D3/6-31G(d)/LanL2DZ
Thermal correction to Gibbs Free Energy = 0.483696
Sum of electronic and thermal Free Energies = -2815.056909
SMD(DCE)-B3LYP-D3/6-311++G(d,p)/SDD
Single point energy = -2817.5964634
Corrected quasi-harmonic Free Energy = -2817.119278
N_{imag} = 0

Cl	0.124393	-2.692503	-2.188483
P	-1.795963	-0.628348	-0.034424
C	-0.168245	1.938619	1.143536
C	-1.381795	2.153853	0.382311
C	-2.180423	1.166293	-0.453663
H	-1.786275	1.200404	-1.476190
C	0.212539	3.191430	1.734011
C	-1.718223	3.538166	0.527562
C	-0.739363	4.172687	1.348138
H	-0.706134	5.226314	1.594480
H	-2.553953	4.034989	0.055123
H	1.103383	3.356980	2.324805
C	-3.674429	1.531941	-0.540743
H	-3.786882	2.505051	-1.031101
H	-4.152329	1.592770	0.440454
H	-4.221675	0.801871	-1.141749
Fe	0.165938	3.329285	-0.331081
C	2.102158	3.531723	-1.048441
C	1.417042	2.499524	-1.763028
C	0.272429	3.080739	-2.387098
C	1.380981	4.750745	-1.236285
C	0.249755	4.472229	-2.063064
H	1.623161	5.707594	-0.791674
H	-0.512429	5.181876	-2.358698
H	-0.471882	2.548773	-2.965972
H	1.677649	1.448219	-1.784529
H	2.986472	3.405177	-0.437224

C	-3.008223	-1.497851	-1.111138
C	-4.080383	-2.242794	-0.601451
C	-2.879010	-1.348701	-2.502511
C	-5.005993	-2.831167	-1.468917
H	-4.204950	-2.365095	0.469343
C	-3.812613	-1.922984	-3.364120
H	-2.041376	-0.792932	-2.910673
C	-4.877491	-2.669632	-2.849279
H	-5.830590	-3.409401	-1.060392
H	-3.702799	-1.796714	-4.437932
H	-5.600728	-3.123135	-3.521867
C	-2.365239	-0.960171	1.682232
C	-2.072900	-2.232357	2.208173
C	-2.963840	-0.003528	2.514207
C	-2.390469	-2.545074	3.529029
H	-1.584548	-2.974112	1.581156
C	-3.267506	-0.314665	3.843647
H	-3.171392	0.995457	2.150619
C	-2.985635	-1.583497	4.352653
H	-2.161204	-3.533187	3.918688
H	-3.721894	0.440252	4.479697
H	-3.221524	-1.821711	5.386269
P	0.816027	0.422148	1.280445
C	0.681784	0.018838	3.066640
H	1.141996	0.820051	3.655503
H	-0.369528	-0.071512	3.347073
H	1.185677	-0.928069	3.278692
O	2.376319	1.004006	1.328340
H	2.727976	1.051079	0.421346
Rh	0.424569	-1.084682	-0.301657
H	2.327424	-1.413345	-2.660730
C	4.741498	-1.817731	-0.803803
C	3.650099	-1.685422	0.104401
C	3.834210	-1.818214	1.498010
C	5.119560	-2.067469	1.930073
C	6.218114	-2.186581	1.024146
C	6.053738	-2.067661	-0.338897
H	3.001764	-1.734880	2.185096
H	5.310115	-2.180627	2.993438
H	7.206652	-2.382004	1.429503
H	6.881139	-2.164642	-1.034230
N	2.536321	-1.450377	-0.637106
N	2.990641	-1.455461	-1.889320
N	4.274431	-1.672801	-2.068791

INT2*

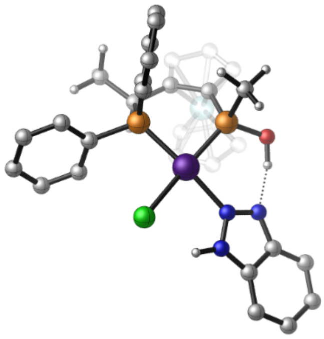

SMD(DCE)-B3LYP-D3/6-31G(d)/LanL2DZ
Thermal correction to Gibbs Free Energy = 0.485940
Sum of electronic and thermal Free Energies = -2815.068357
SMD(DCE)-B3LYP-D3/6-311++G(d,p)/SDD
Single point energy = -2817.6089134

Corrected quasi-harmonic Free Energy = -2817.130037
N_{imag} = 0

Cl	-0.468428	-3.402639	0.670731
P	1.800223	-0.728013	0.137386
C	0.621711	2.165871	-0.888707
C	1.613897	2.124400	0.163922
C	2.120484	0.933048	0.961054
H	1.476818	0.826222	1.840990
C	0.461237	3.535071	-1.292376
C	2.043396	3.473999	0.380605
C	1.333595	4.335933	-0.506776
H	1.415351	5.414813	-0.541978
H	2.758599	3.796427	1.124185
H	-0.245912	3.888265	-2.030689
C	3.557599	1.131748	1.479302
H	3.588153	1.985627	2.164500
H	4.271182	1.320076	0.673046
H	3.896327	0.255914	2.038036
Fe	-0.002773	3.294511	0.708967
C	-2.009488	3.748885	0.959606
C	-1.740465	2.429725	1.437365
C	-0.767548	2.526969	2.477521
C	-1.203485	4.662414	1.707592
C	-0.433766	3.906350	2.645466
H	-1.151913	5.733604	1.559385
H	0.300398	4.304529	3.334254
H	-0.325904	1.693579	3.009016
H	-2.149368	1.510795	1.039374
H	-2.670613	4.003828	0.141769
C	2.527747	-1.847671	1.405771
C	3.741269	-2.524907	1.228385
C	1.854869	-1.958037	2.633916
C	4.269414	-3.306344	2.260778
H	4.283072	-2.444049	0.291993
C	2.391682	-2.724228	3.667439
H	0.904958	-1.451012	2.774915
C	3.599493	-3.405023	3.481364
H	5.208621	-3.831714	2.108675
H	1.862048	-2.799810	4.613407
H	4.013657	-4.009627	4.283910
C	2.906540	-0.859524	-1.323840
C	2.908794	-2.083348	-2.017690
C	3.641179	0.216922	-1.842698
C	3.651744	-2.234132	-3.187685
H	2.324613	-2.917961	-1.639238
C	4.375385	0.066317	-3.023273
H	3.628092	1.183943	-1.354090
C	4.386937	-1.157301	-3.694881
H	3.648790	-3.187942	-3.708195
H	4.935018	0.910763	-3.416517
H	4.958309	-1.271330	-4.612086
P	-0.358941	0.795179	-1.549929
C	0.290553	0.591977	-3.256026
H	0.219012	1.542983	-3.796274
H	1.331821	0.263004	-3.237227
H	-0.312317	-0.163529	-3.770182
O	-1.795455	1.522191	-1.889780
H	-2.532160	1.020624	-1.446692
Rh	-0.411813	-1.068004	-0.326952
H	-2.623236	-3.120090	0.289038
C	-4.552894	-2.126332	-0.039571

C	-4.681369	-0.799974	-0.510733
C	-5.947756	-0.207420	-0.670745
C	-7.045001	-0.984413	-0.342082
C	-6.903636	-2.316939	0.132195
C	-5.664608	-2.916029	0.294025
H	-6.045619	0.810341	-1.033896
H	-8.043758	-0.571625	-0.447677
H	-7.798861	-2.881933	0.374812
H	-5.555029	-3.932914	0.654731
N	-3.424377	-0.308044	-0.738781
N	-2.557872	-1.241599	-0.437942
N	-3.209730	-2.337084	-0.025133

TS1-3*

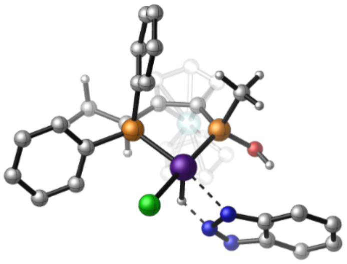

SMD(DCE)-B3LYP-D3/6-31G(d)/LanL2DZ
Thermal correction to Gibbs Free Energy = 0.477006
Sum of electronic and thermal Free Energies = -2815.018750
SMD(DCE)-B3LYP-D3/6-311++G(d,p)/SDD
Single point energy = -2817.5571767
Corrected quasi-harmonic Free Energy = -2817.086898
N_{imag} = 1, ν = -1202.5993i cm^{-1}

Cl	-1.217971	-3.489600	-0.953935
P	-1.930477	-0.196434	-0.119503
C	0.462900	1.771913	1.020083
C	-0.598447	2.284927	0.178901
C	-1.504446	1.516481	-0.763944
H	-0.923261	1.242305	-1.652079
C	1.056421	2.881129	1.715668
C	-0.639912	3.699517	0.387174
C	0.377563	4.062995	1.318602
H	0.618596	5.070753	1.631017
H	-1.301715	4.390948	-0.114454
H	1.897388	2.817925	2.393160
C	-2.711156	2.342385	-1.237993
H	-2.356587	3.246430	-1.744413
H	-3.360411	2.643005	-0.410090
H	-3.310319	1.779434	-1.956732
Fe	1.222658	3.111444	-0.334727
C	3.236680	3.177051	-0.822848
C	2.592691	2.126179	-1.544642
C	1.559729	2.707943	-2.339980
C	2.603584	4.409316	-1.175200
C	1.565344	4.119271	-2.112940
H	2.839675	5.385354	-0.770654
H	0.876623	4.836066	-2.541562
H	0.864833	2.167823	-2.970540
H	2.814844	1.070834	-1.462518
H	4.034852	3.053958	-0.102483
C	-3.230331	-0.737107	-1.287811
C	-4.582866	-0.785888	-0.926061
C	-2.853844	-1.028612	-2.608836
C	-5.548920	-1.129788	-1.876075
H	-4.888001	-0.556601	0.089810

C	-3.822425	-1.355423	-3.556311
H	-1.805445	-1.010414	-2.890105
C	-5.171824	-1.410839	-3.190464
H	-6.595379	-1.172199	-1.586269
H	-3.523691	-1.579434	-4.576688
H	-5.924625	-1.675219	-3.928216
C	-2.728618	-0.062916	1.523365
C	-3.026182	-1.273497	2.177174
C	-2.956714	1.150551	2.189023
C	-3.550733	-1.266525	3.468898
H	-2.843501	-2.219249	1.672954
C	-3.473910	1.151251	3.487586
H	-2.720632	2.096129	1.715308
C	-3.770529	-0.052929	4.129102
H	-3.779085	-2.206905	3.962845
H	-3.641192	2.096551	3.996581
H	-4.168677	-0.047781	5.140003
P	0.925786	0.056080	1.253364
C	0.625170	-0.247968	3.037794
H	1.137599	0.508139	3.642308
H	-0.450107	-0.210293	3.237553
H	0.999007	-1.243240	3.301944
O	2.571658	0.168323	1.159116
H	2.984653	-0.713324	1.226162
Rh	-0.096309	-1.533343	0.026526
H	0.813415	-1.132592	-1.348857
C	4.029273	-2.026305	-0.862591
C	3.182020	-2.702829	0.062765
C	3.707359	-3.348349	1.204171
C	5.079523	-3.299759	1.372419
C	5.930759	-2.641020	0.438684
C	5.428578	-2.004519	-0.681524
H	3.056948	-3.855609	1.910464
H	5.528013	-3.779429	2.237963
H	7.001818	-2.641022	0.620160
H	6.074030	-1.499061	-1.393337
N	1.909254	-2.550171	-0.386025
N	2.014908	-1.821262	-1.530897
N	3.246883	-1.462604	-1.830090

TS2-4*

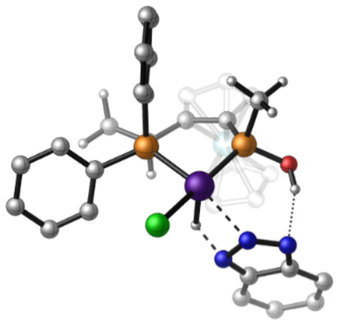

SMD(DCE)-B3LYP-D3/6-31G(d)/LanL2DZ
Thermal correction to Gibbs Free Energy = 0.479128
Sum of electronic and thermal Free Energies = -2815.021245
SMD(DCE)-B3LYP-D3/6-311++G(d,p)/SDD
Single point energy = -2817.5598341
Corrected quasi-harmonic Free Energy = -2817.088055
N_{imag} = 1, ν = -1213.2829i cm^{-1}

Cl	1.765425	-3.458960	-0.579884
P	1.920442	-0.076623	0.290383
C	-0.506821	1.969638	-0.718863

C	0.178173	2.165414	0.542373
C	0.982564	1.161710	1.350035
H	0.280100	0.482400	1.846509
C	-1.162528	3.199865	-1.067228
C	-0.068429	3.518583	0.935980
C	-0.893513	4.146043	-0.043191
H	-1.282946	5.154406	0.010464
H	0.274651	3.980949	1.850329
H	-1.779787	3.355784	-1.941871
C	1.832667	1.823701	2.447478
H	1.180617	2.383433	3.126274
H	2.576403	2.514061	2.039166
H	2.354513	1.071256	3.042623
Fe	-1.856183	2.467608	0.740405
C	-3.931918	2.423366	0.700172
C	-3.413638	1.094726	0.728513
C	-2.629916	0.951518	1.914098
C	-3.472596	3.103546	1.870810
C	-2.668875	2.191404	2.623113
H	-3.666015	4.138422	2.122845
H	-2.143806	2.415307	3.543030
H	-2.071566	0.069051	2.198829
H	-3.535681	0.348240	-0.042933
H	-4.529419	2.854563	-0.092817
C	2.889260	-0.982113	1.553328
C	4.263864	-0.777506	1.731568
C	2.198350	-1.842979	2.421183
C	4.939719	-1.431241	2.765922
H	4.810648	-0.111116	1.072277
C	2.873656	-2.481090	3.460180
H	1.137415	-2.021638	2.275485
C	4.247309	-2.279282	3.632203
H	6.006966	-1.271899	2.893840
H	2.331029	-3.145259	4.127313
H	4.774391	-2.784038	4.437295
C	3.125415	0.804937	-0.765069
C	3.913059	0.016984	-1.624649
C	3.229430	2.202282	-0.823025
C	4.801312	0.619814	-2.514029
H	3.826528	-1.066352	-1.595700
C	4.113648	2.801273	-1.724642
H	2.618310	2.830771	-0.186009
C	4.900445	2.014271	-2.568050
H	5.409077	0.002348	-3.169545
H	4.183873	3.884746	-1.765604
H	5.586181	2.483998	-3.267808
P	-0.472234	0.489547	-1.727654
C	0.423043	1.011684	-3.238879
H	-0.087929	1.865043	-3.698386
H	1.448090	1.294220	-2.982682
H	0.447732	0.178056	-3.948884
O	-2.024232	0.357356	-2.256445
H	-2.253121	-0.585595	-2.425612
Rh	0.517543	-1.374663	-0.920188
H	-0.570107	-1.759078	0.304464
C	-3.016750	-2.465535	0.208126
C	-3.576828	-2.391555	-1.091118
C	-4.965017	-2.243579	-1.278297
C	-5.747376	-2.163279	-0.138537
C	-5.181172	-2.240396	1.164407
C	-3.820089	-2.398917	1.362140
H	-5.392938	-2.189667	-2.274603

H	-6.822466	-2.040692	-0.234477
H	-5.840757	-2.173581	2.024992
H	-3.387612	-2.459142	2.355723
N	-2.548845	-2.444104	-2.002163
N	-1.438666	-2.577079	-1.306863
N	-1.668379	-2.605545	0.035565

INT3*

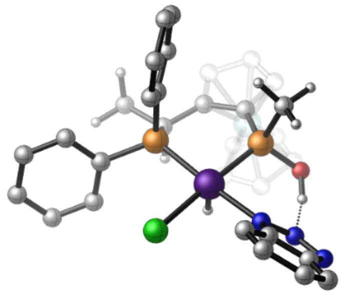

SMD(DCE)-B3LYP-D3/6-31G(d)/LanL2DZ
Thermal correction to Gibbs Free Energy = 0.480560
Sum of electronic and thermal Free Energies = -2815.061239
SMD(DCE)-B3LYP-D3/6-311++G(d,p)/SDD
Single point energy = -2817.6062191
Corrected quasi-harmonic Free Energy = -2817.132253
N_{imag} = 0

Cl	2.700654	-1.523824	-1.544330
P	-0.405716	-1.672378	-0.047168
C	-1.862869	1.122270	1.019248
C	-2.629148	-0.008662	0.536883
C	-2.176342	-1.150031	-0.353467
H	-2.087484	-0.774624	-1.379767
C	-2.740894	1.953001	1.799234
C	-3.958029	0.148754	1.045207
C	-4.026007	1.351125	1.807613
H	-4.915038	1.756100	2.273098
H	-4.790737	-0.509936	0.846144
H	-2.466869	2.889017	2.267705
C	-3.173715	-2.320426	-0.368286
H	-4.144698	-1.967582	-0.731028
H	-3.312882	-2.751126	0.628068
H	-2.839755	-3.110868	-1.043427
Fe	-3.419492	1.767861	-0.147133
C	-3.343306	3.631349	-1.044838
C	-2.631931	2.675900	-1.833746
C	-3.546312	1.647353	-2.212458
C	-4.700037	3.194385	-0.940685
C	-4.825663	1.966882	-1.660738
H	-5.484418	3.682335	-0.376274
H	-5.721226	1.363742	-1.738852
H	-3.302149	0.757049	-2.778422
H	-1.571593	2.697904	-2.044338
H	-2.916249	4.506631	-0.573562
C	-0.243306	-3.152601	-1.102336
C	-0.225137	-4.449174	-0.571596
C	-0.214933	-2.968758	-2.493832
C	-0.175254	-5.552455	-1.427935
H	-0.251050	-4.604092	0.502265
C	-0.180408	-4.073288	-3.343414
H	-0.207770	-1.965930	-2.909834
C	-0.156510	-5.367028	-2.811633
H	-0.155456	-6.555343	-1.010261
H	-0.158739	-3.924215	-4.419354

H	-0.120365	-6.226646	-3.475360
C	-0.172683	-2.198457	1.686724
C	1.119964	-2.625259	2.050571
C	-1.162833	-2.079947	2.674053
C	1.409859	-2.933798	3.379399
H	1.893685	-2.717319	1.291811
C	-0.862768	-2.385217	4.004154
H	-2.161384	-1.743574	2.420424
C	0.419893	-2.809555	4.359271
H	2.408359	-3.266367	3.648910
H	-1.635283	-2.289330	4.762064
H	0.648124	-3.042470	5.395609
P	-0.114878	1.470197	0.813185
C	0.524345	1.579010	2.522103
H	-0.024063	2.346227	3.079601
H	0.401564	0.610046	3.017410
H	1.587283	1.839173	2.490092
O	-0.066880	2.981879	0.224515
H	0.786052	3.093887	-0.315356
Rh	1.168936	-0.005263	-0.344061
H	0.416762	0.436573	-1.593580
C	4.460475	2.825554	-0.484428
C	3.993199	1.512574	-0.225189
C	4.866139	0.499396	0.222191
C	6.195451	0.846897	0.389781
C	6.670670	2.161595	0.121715
C	5.820410	3.161047	-0.316059
H	4.507193	-0.506870	0.404219
H	6.902300	0.095611	0.732283
H	7.725686	2.377696	0.267421
H	6.174096	4.167678	-0.519610
N	2.651827	1.539613	-0.476240
N	2.343462	2.796422	-0.854886
N	3.393241	3.589273	-0.869977

INT4*

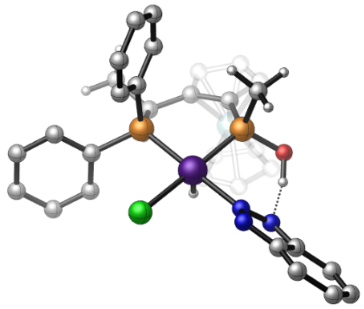

SMD(DCE)-B3LYP-D3/6-31G(d)/LanL2DZ
Thermal correction to Gibbs Free Energy = 0.481025
Sum of electronic and thermal Free Energies = -2815.059813
SMD(DCE)-B3LYP-D3/6-311++G(d,p)/SDD
Single point energy = -2817.6034351
Corrected quasi-harmonic Free Energy = -2817.129541
N_{imag} = 0

Cl	0.757806	-3.064140	-1.356764
P	-1.644954	-1.005947	-0.078815
C	-0.742902	1.940995	1.268114
C	-2.035482	1.764109	0.638956
C	-2.578902	0.611673	-0.189617
H	-2.408211	0.852600	-1.245137
C	-0.721436	3.239202	1.888884
C	-2.777461	2.962810	0.890351

C	-1.973881	3.861855	1.649747
H	-2.254899	4.862565	1.951031
H	-3.773496	3.173453	0.527619
H	0.125055	3.672425	2.404209
C	-4.098953	0.421453	-0.006591
H	-4.626460	1.316876	-0.348786
H	-4.366971	0.244702	1.038085
H	-4.466097	-0.416831	-0.603991
Fe	-1.023890	3.388815	-0.149378
C	0.596086	4.398338	-0.954445
C	0.426127	3.142850	-1.613597
C	-0.874562	3.125152	-2.202260
C	-0.598924	5.159626	-1.140992
C	-1.509449	4.372096	-1.911261
H	-0.798626	6.141130	-0.730086
H	-2.517041	4.654033	-2.188950
H	-1.317365	2.294324	-2.737157
H	1.134394	2.325585	-1.611822
H	1.458184	4.694157	-0.371672
C	-2.420850	-1.919326	-1.460824
C	-3.432552	-2.870559	-1.274038
C	-2.026863	-1.571287	-2.762667
C	-4.040687	-3.468819	-2.381472
H	-3.751527	-3.146247	-0.274143
C	-2.645810	-2.160608	-3.863688
H	-1.226353	-0.852485	-2.912062
C	-3.651960	-3.113637	-3.674554
H	-4.821129	-4.209307	-2.229553
H	-2.334699	-1.885343	-4.867609
H	-4.128382	-3.578669	-4.533303
C	-2.056302	-1.854747	1.481649
C	-1.738248	-3.220847	1.594747
C	-2.525916	-1.158134	2.606534
C	-1.918410	-3.883443	2.808845
H	-1.344153	-3.757143	0.736025
C	-2.699691	-1.828074	3.820049
H	-2.742630	-0.096629	2.551225
C	-2.400641	-3.189114	3.922450
H	-1.676137	-4.939774	2.884667
H	-3.066807	-1.282942	4.685073
H	-2.536988	-3.706344	4.868105
P	0.684983	0.858610	1.274030
C	0.857093	0.268705	2.994037
H	0.951885	1.123395	3.672984
H	-0.019838	-0.323608	3.272406
H	1.753451	-0.356526	3.065351
O	1.939298	1.863722	1.059877
H	2.597901	1.440854	0.410827
Rh	0.670679	-0.940432	-0.104660
H	0.581188	0.047835	-1.261826
C	4.857788	-1.435185	-0.036972
C	4.773972	-0.058445	-0.373712
C	5.933017	0.711369	-0.607690
C	7.149573	0.061793	-0.497720
C	7.236183	-1.320398	-0.163448
C	6.106606	-2.083694	0.071473
H	5.866456	1.764398	-0.865773
H	8.068600	0.615159	-0.672271
H	8.218317	-1.780471	-0.095260
H	6.169671	-3.138265	0.323840
N	3.453782	0.264376	-0.408677
N	2.801042	-0.871812	-0.106700

N 3.587520 -1.904885 0.127878

INT1-p

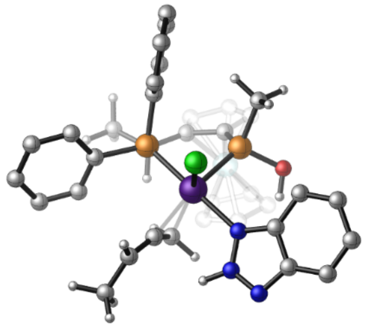

SMD(DCE)-B3LYP-D3/6-31G(d)/LanL2DZ
Thermal correction to Gibbs Free Energy = 0.566957
Sum of electronic and thermal Free Energies = -2970.961239
SMD(DCE)-B3LYP-D3/6-311++G(d,p)/SDD
Single point energy = -2973.6349532
Corrected quasi-harmonic Free Energy = -2973.076256
N_{imag} = 0

Cl	-1.242692	2.788057	1.585289
P	1.660746	0.830824	0.145966
C	0.681304	-2.212273	0.868678
C	1.889212	-1.997124	0.095898
C	2.381322	-0.737535	-0.606578
H	1.934183	-0.720270	-1.602495
C	0.691153	-3.572900	1.328472
C	2.613034	-3.233752	0.108821
C	1.875317	-4.198193	0.856022
H	2.149515	-5.236219	0.994632
H	3.547288	-3.422764	-0.400485
H	-0.100402	-4.043434	1.896360
C	3.908568	-0.724416	-0.809133
H	4.199724	-1.566872	-1.445257
H	4.461414	-0.801215	0.130432
H	4.227538	0.188553	-1.316398
Fe	0.734767	-3.496770	-0.739122
C	0.120543	-5.145820	-1.840943
C	-1.009122	-4.357961	-1.460219
C	-0.838538	-3.050667	-2.010612
C	0.988431	-4.325036	-2.624982
C	0.395560	-3.029263	-2.728822
H	1.946791	-4.620885	-3.032307
H	0.823694	-2.169288	-3.226814
H	-1.499696	-2.206445	-1.875130
H	-1.826233	-4.679560	-0.827697
H	0.308438	-6.170916	-1.548124
C	2.591648	2.180554	-0.694918
C	3.231735	3.208923	0.010910
C	2.635257	2.194863	-2.101428
C	3.891258	4.233882	-0.673991
H	3.227527	3.219232	1.094685
C	3.305796	3.210801	-2.780876
H	2.156442	1.408665	-2.673119
C	3.930710	4.239026	-2.068593
H	4.379274	5.024105	-0.109953
H	3.335377	3.199909	-3.867049
H	4.447124	5.034827	-2.598437
C	2.211842	0.864254	1.902148
C	1.790792	1.925341	2.722676
C	2.998150	-0.154603	2.467647

C	2.156628	1.970552	4.068548
H	1.147492	2.696374	2.316619
C	3.353414	-0.110006	3.817544
H	3.321017	-1.003866	1.881132
C	2.936078	0.952178	4.622157
H	1.819281	2.799018	4.685470
H	3.955238	-0.911781	4.236690
H	3.212222	0.983422	5.672723
P	-0.655424	-1.035915	1.237315
C	-0.582098	-0.991198	3.072691
H	-0.780645	-1.988050	3.481619
H	0.414722	-0.669342	3.384556
H	-1.313848	-0.280008	3.465887
O	-2.027498	-1.977176	1.097237
H	-2.416489	-1.842140	0.214970
Rh	-0.640267	0.882526	-0.161098
H	-2.779002	1.680444	-2.328966
C	-5.068496	0.981151	-0.409412
C	-3.903040	0.701444	0.364072
C	-4.006450	0.319182	1.719768
C	-5.278770	0.199401	2.236118
C	-6.447347	0.450754	1.453995
C	-6.366257	0.845336	0.136822
H	-3.128264	0.152154	2.324174
H	-5.402441	-0.085460	3.277027
H	-7.421024	0.337154	1.921831
H	-7.246089	1.055697	-0.462445
N	-2.827520	0.917937	-0.438571
N	-3.373245	1.312978	-1.586679
N	-4.684006	1.382258	-1.645523
C	-0.540278	1.888290	-1.973332
C	-0.449777	0.505599	-2.266070
H	0.485176	0.074037	-2.620103
H	-1.318339	-0.039084	-2.643285
C	-0.594111	3.112583	-2.488423
H	-0.670808	3.967290	-1.814414
C	-0.497968	3.433643	-3.959701
H	-1.347198	4.045124	-4.295161
H	0.412116	4.014666	-4.169168
H	-0.466705	2.521801	-4.566713

INT2-p

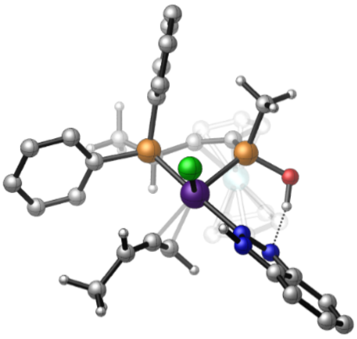

SMD(DCE)-B3LYP-D3/6-31G(d)/LanL2DZ
Thermal correction to Gibbs Free Energy = 0.564796
Sum of electronic and thermal Free Energies = -2970.982046
SMD(DCE)-B3LYP-D3/6-311++G(d,p)/SDD
Single point energy = -2973.6512421
Corrected quasi-harmonic Free Energy = -2973.093875
N_{imag} = 0

Cl	1.192064	-3.075402	1.784527

P	-1.742965	-0.876021	-0.090192
C	-1.081883	2.184152	0.891939
C	-2.107502	1.930437	-0.103572
C	-2.383627	0.677284	-0.926920
H	-1.735092	0.716174	-1.806696
C	-1.244077	3.534650	1.352902
C	-2.876172	3.133738	-0.219153
C	-2.341424	4.116478	0.664507
H	-2.686118	5.138389	0.758156
H	-3.700918	3.290615	-0.899715
H	-0.600961	4.029295	2.068719
C	-3.833650	0.609911	-1.442160
H	-4.018274	1.446607	-2.124258
H	-4.571259	0.661278	-0.637326
H	-4.009155	-0.310361	-2.003373
Fe	-0.881951	3.488105	-0.684907
C	-0.094709	5.165045	-1.621762
C	0.953222	4.381199	-1.047389
C	0.911486	3.083194	-1.643538
C	-0.786477	4.350512	-2.570367
C	-0.163758	3.064203	-2.582355
H	-1.654423	4.643150	-3.147499
H	-0.477487	2.209991	-3.168537
H	1.545423	2.243977	-1.392372
H	1.629341	4.697104	-0.263857
H	-0.349560	6.182104	-1.352198
C	-2.398670	-2.265165	-1.104518
C	-2.958805	-3.406973	-0.513607
C	-2.251034	-2.228985	-2.503377
C	-3.356785	-4.489861	-1.302460
H	-3.088767	-3.462301	0.561527
C	-2.658156	-3.307266	-3.288199
H	-1.826069	-1.357642	-2.990375
C	-3.207302	-4.444998	-2.689292
H	-3.787696	-5.366781	-0.826932
H	-2.540507	-3.259048	-4.367336
H	-3.517979	-5.287855	-3.300680
C	-2.565932	-1.009213	1.547039
C	-2.097548	-1.990717	2.438241
C	-3.575813	-0.130860	1.971855
C	-2.644663	-2.104314	3.716586
H	-1.281231	-2.642233	2.143312
C	-4.110239	-0.239523	3.257957
H	-3.937400	0.658350	1.325675
C	-3.650398	-1.227476	4.131694
H	-2.270049	-2.868070	4.392644
H	-4.884851	0.453552	3.574708
H	-4.067170	-1.307914	5.132069
P	0.229791	1.073155	1.479395
C	-0.149303	0.943833	3.270206
H	-0.090099	1.932493	3.738967
H	-1.154098	0.537770	3.412097
H	0.575498	0.271002	3.739724
O	1.562179	2.052698	1.580981
H	2.206655	1.804777	0.879343
Rh	0.556133	-0.782087	0.063788
H	3.040610	-2.393875	0.867402
C	4.788704	-1.103622	0.489376
C	4.683755	0.257794	0.119728
C	5.831855	1.059634	-0.017302
C	7.051036	0.451655	0.227733
C	7.143423	-0.916928	0.600574

C	6.024073	-1.721655	0.739260
H	5.752471	2.103829	-0.301744
H	7.965488	1.029875	0.134976
H	8.125853	-1.343118	0.781615
H	6.092429	-2.766125	1.023262
N	3.353551	0.558630	-0.040647
N	2.676833	-0.529030	0.208882
N	3.498643	-1.536335	0.514338
C	0.916091	-1.946623	-1.655850
C	0.829753	-0.622346	-2.087584
H	-0.043479	-0.264779	-2.631410
H	1.730929	-0.036626	-2.271057
C	1.071165	-3.235497	-1.916436
H	1.007916	-3.945247	-1.091153
C	1.288928	-3.812575	-3.294132
H	2.204303	-4.419542	-3.336365
H	0.455957	-4.475168	-3.570785
H	1.363058	-3.024794	-4.052046

TS1-3-p

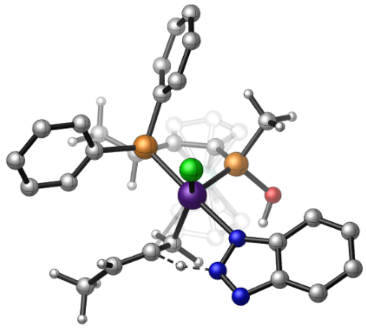

SMD(DCE)-B3LYP-D3/6-31G(d)/LanL2DZ
Thermal correction to Gibbs Free Energy = 0.563985
Sum of electronic and thermal Free Energies = -2970.937829
SMD(DCE)-B3LYP-D3/6-311++G(d,p)/SDD
Single point energy = -2973.6081818
Corrected quasi-harmonic Free Energy = -2973.053183
N_{imag} = 1, ν = -1419.8205i cm^{-1}

Cl	2.320825	-2.455591	1.026926
P	-1.092688	-1.448676	0.195902
C	-1.449494	1.666105	0.939214
C	-2.510474	0.959415	0.248984
C	-2.434376	-0.345984	-0.526754
H	-2.029415	-0.132305	-1.518962
C	-2.028339	2.822906	1.566119
C	-3.716149	1.693953	0.481057
C	-3.419103	2.835479	1.281632
H	-4.123033	3.603382	1.575272
H	-4.689624	1.446304	0.082518
H	-1.482357	3.568504	2.128835
C	-3.813081	-0.992814	-0.750680
H	-4.432853	-0.314877	-1.347970
H	-4.343126	-1.204965	0.180814
H	-3.722855	-1.927736	-1.306720
Fe	-2.318626	2.890690	-0.481981
C	-2.806778	4.688754	-1.391786
C	-1.392303	4.599131	-1.211547
C	-0.931152	3.450701	-1.923550
C	-3.218513	3.593922	-2.211821
C	-2.058509	2.826984	-2.539454
H	-4.236445	3.361571	-2.497563
H	-2.042691	1.916671	-3.124245
H	0.088128	3.095161	-1.962916

H	-0.782944	5.260408	-0.609361
H	-3.459333	5.428036	-0.945207
C	-1.328130	-3.084456	-0.600051
C	-1.255680	-4.280056	0.128420
C	-1.541959	-3.140094	-1.987945
C	-1.391496	-5.508947	-0.522615
H	-1.095296	-4.262904	1.200634
C	-1.689657	-4.367771	-2.631709
H	-1.602607	-2.226033	-2.568018
C	-1.608959	-5.556881	-1.900639
H	-1.330357	-6.428025	0.053608
H	-1.864371	-4.394383	-3.703720
H	-1.718034	-6.514052	-2.403149
C	-1.505063	-1.677704	1.980260
C	-0.562732	-2.295175	2.821608
C	-2.705102	-1.210296	2.543416
C	-0.821532	-2.451704	4.184076
H	0.384688	-2.628890	2.412447
C	-2.953240	-1.356849	3.909827
H	-3.449005	-0.709813	1.940202
C	-2.014739	-1.979444	4.735189
H	-0.079087	-2.932658	4.815237
H	-3.883884	-0.979678	4.324978
H	-2.210331	-2.091241	5.798291
P	0.288933	1.196202	1.134590
C	0.490034	1.153183	2.952790
H	0.356892	2.158076	3.368190
H	-0.257469	0.481387	3.382098
H	1.486906	0.777956	3.200484
O	1.138177	2.585492	0.822960
H	1.442564	2.578238	-0.103314
Rh	1.011320	-0.508994	-0.165143
H	2.650293	-0.871350	-2.044710
C	5.229331	0.649629	-0.509687
C	4.058638	0.886214	0.265704
C	4.118529	1.619615	1.469793
C	5.358798	2.097438	1.849512
C	6.530205	1.867954	1.072072
C	6.486470	1.149500	-0.106943
H	3.232049	1.807090	2.060735
H	5.448182	2.666095	2.771147
H	7.477222	2.268997	1.422345
H	7.374577	0.966221	-0.703941
N	3.036888	0.276300	-0.395240
N	3.594612	-0.282006	-1.469886
N	4.883570	-0.095462	-1.601477
C	1.254112	-0.964930	-2.397491
C	0.628018	0.290181	-2.107376
H	-0.422282	0.457243	-2.335692
H	1.228197	1.194968	-2.223499
C	1.056420	-1.911001	-3.337994
H	1.550790	-2.876774	-3.215963
C	0.274349	-1.743761	-4.609062
H	0.944831	-1.725667	-5.480605
H	-0.403828	-2.595040	-4.758516
H	-0.307904	-0.815917	-4.608807

TS2-4-p

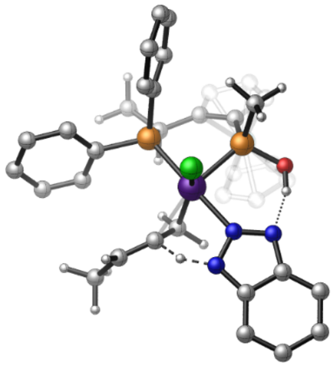

SMD(DCE)-B3LYP-D3/6-31G(d)/LanL2DZ
Thermal correction to Gibbs Free Energy = 0.564918
Sum of electronic and thermal Free Energies = -2970.948437
SMD(DCE)-B3LYP-D3/6-311++G(d,p)/SDD
Single point energy = -2973.6182147
Corrected quasi-harmonic Free Energy = -2973.062440
N_{imag} = 1, ν = -1539.9755i cm^{-1}

Cl	0.908699	-2.355543	2.385529
P	-1.672454	-0.953235	-0.048650
C	-1.086592	2.179216	0.764685
C	-2.062143	1.855198	-0.257052
C	-2.276746	0.549918	-1.013274
H	-1.594064	0.545549	-1.866807
C	-1.309751	3.537265	1.177424
C	-2.861493	3.028705	-0.446062
C	-2.396708	4.056531	0.425925
H	-2.778175	5.068435	0.471683
H	-3.665179	3.135336	-1.160495
H	-0.714877	4.075113	1.903529
C	-3.703053	0.429705	-1.583316
H	-3.875844	1.237832	-2.301838
H	-4.472401	0.492116	-0.809636
H	-3.834951	-0.512269	-2.119206
Fe	-0.858911	3.435723	-0.838294
C	-0.178231	5.096865	-1.879796
C	0.889693	4.504787	-1.136701
C	1.050113	3.159530	-1.588678
C	-0.679124	4.115971	-2.790132
C	0.081200	2.919250	-2.610255
H	-1.512502	4.244680	-3.469060
H	-0.078479	1.980863	-3.124316
H	1.734343	2.432746	-1.174170
H	1.446445	4.974528	-0.336471
H	-0.568993	6.097467	-1.745454
C	-2.272237	-2.366646	-1.073416
C	-3.028739	-3.418768	-0.536205
C	-1.982414	-2.378964	-2.449633
C	-3.475877	-4.461208	-1.352880
H	-3.285724	-3.433219	0.516111
C	-2.449782	-3.408707	-3.265576
H	-1.405643	-1.578164	-2.898581
C	-3.192739	-4.458715	-2.718813
H	-4.056615	-5.268669	-0.915365
H	-2.228907	-3.390347	-4.328966
H	-3.550379	-5.264187	-3.354333
C	-2.663210	-1.024524	1.498492
C	-2.415337	-2.069689	2.405979
C	-3.612109	-0.045376	1.837561
C	-3.111201	-2.140598	3.613163

H	-1.656231	-2.808967	2.183367
C	-4.297888	-0.114301	3.052292
H	-3.812799	0.789534	1.179833
C	-4.051966	-1.161634	3.942350
H	-2.904892	-2.955573	4.301452
H	-5.022771	0.656437	3.299553
H	-4.585707	-1.211953	4.887559
P	0.193563	1.115564	1.453269
C	-0.286935	0.928414	3.210510
H	-0.348408	1.917854	3.676901
H	-1.251066	0.422745	3.295100
H	0.472796	0.322054	3.710938
O	1.484477	2.118701	1.554512
H	2.339051	1.605550	1.458387
Rh	0.641634	-0.811671	0.279015
H	2.534360	-2.033581	-0.805355
C	4.774213	-1.184700	0.057568
C	4.792378	0.011251	0.820212
C	6.006286	0.629882	1.173754
C	7.169924	0.019572	0.737340
C	7.145879	-1.178564	-0.027885
C	5.959878	-1.802616	-0.377944
H	6.019255	1.542467	1.761330
H	8.130537	0.462231	0.984660
H	8.089172	-1.615450	-0.343214
H	5.940244	-2.719518	-0.958046
N	3.492351	0.362826	1.076210
N	2.745653	-0.555046	0.518434
N	3.454996	-1.503880	-0.084753
C	1.327989	-1.885134	-1.554425
C	1.050535	-0.492975	-1.791690
H	0.209783	-0.207478	-2.422366
H	1.890916	0.196392	-1.888004
C	1.205307	-3.015677	-2.283606
H	1.331675	-3.973726	-1.774677
C	1.031593	-3.096269	-3.770731
H	1.956537	-3.460371	-4.242101
H	0.245892	-3.817071	-4.029015
H	0.786143	-2.122591	-4.206689

INT3-p

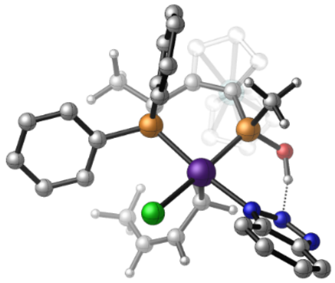

SMD(DCE)-B3LYP-D3/6-31G(d)/LanL2DZ
Thermal correction to Gibbs Free Energy = 0.571113
Sum of electronic and thermal Free Energies = -2971.011945
SMD(DCE)-B3LYP-D3/6-311++G(d,p)/SDD
Single point energy = -2973.6866608
Corrected quasi-harmonic Free Energy = -2973.124384
N_{imag} = 0

Cl	-2.796236	1.771780	-1.029831
P	0.433756	1.644142	0.208652
C	1.782662	-1.210698	1.194385
C	2.593596	-0.085727	0.775624

C	2.199369	1.085631	-0.103109
H	2.118531	0.729139	-1.134312
C	2.598211	-2.062856	2.017984
C	3.886344	-0.269971	1.361039
C	3.888901	-1.480715	2.113218
H	4.741536	-1.904874	2.627318
H	4.738615	0.379233	1.221958
H	2.281701	-2.999082	2.458263
C	3.240995	2.214129	-0.095471
H	4.206315	1.819804	-0.430526
H	3.372374	2.647690	0.900679
H	2.955385	3.011815	-0.784305
Fe	3.404586	-1.870371	0.121544
C	4.811141	-3.210003	-0.604092
C	3.497091	-3.758202	-0.728967
C	2.730925	-2.883424	-1.557788
C	4.855418	-1.995086	-1.354448
C	3.568794	-1.793615	-1.943335
H	5.701331	-1.323591	-1.428760
H	3.270261	-0.943353	-2.542543
H	1.681044	-2.993154	-1.788570
H	3.131038	-4.654039	-0.244510
H	5.617504	-3.618638	-0.008536
C	0.330772	3.284585	-0.594677
C	0.656376	4.453536	0.111858
C	-0.008746	3.367669	-1.951723
C	0.651968	5.688232	-0.539811
H	0.914817	4.404143	1.165042
C	-0.002322	4.603168	-2.600898
H	-0.304252	2.475281	-2.488080
C	0.328052	5.764541	-1.897311
H	0.902277	6.588935	0.014132
H	-0.269584	4.657591	-3.652811
H	0.325973	6.726663	-2.402552
C	0.202093	1.983970	1.995294
C	-1.104514	2.319396	2.399681
C	1.211031	1.864166	2.962329
C	-1.393838	2.521868	3.748787
H	-1.891162	2.421429	1.655441
C	0.913233	2.064683	4.313193
H	2.224482	1.607810	2.678130
C	-0.386279	2.388606	4.709444
H	-2.405605	2.780597	4.048631
H	1.701546	1.966452	5.054500
H	-0.613214	2.539580	5.761222
P	0.034774	-1.504299	0.908944
C	-0.678939	-1.619430	2.588491
H	-0.173087	-2.408037	3.155950
H	-0.555088	-0.660887	3.102388
H	-1.745399	-1.852231	2.507286
O	-0.018316	-3.013804	0.303406
H	-0.882205	-3.116978	-0.212760
Rh	-1.174484	0.019025	-0.256735
H	-1.938952	-0.251107	-3.343824
C	-4.539722	-2.759954	-0.299561
C	-4.050636	-1.449987	-0.059901
C	-4.895623	-0.443428	0.454271
C	-6.214530	-0.789960	0.692708
C	-6.711469	-2.097935	0.433649
C	-5.889487	-3.093902	-0.061020
H	-4.524268	0.557186	0.635856
H	-6.895943	-0.041098	1.088276

H	-7.757562	-2.312333	0.635540
H	-6.254985	-4.098507	-0.253590
N	-2.723282	-1.482022	-0.387365
N	-2.444602	-2.743714	-0.775023
N	-3.499426	-3.529617	-0.739289
C	-0.871644	-0.090720	-3.203731
C	-0.288162	-0.739507	-2.018007
H	0.795357	-0.656140	-1.948145
H	-0.581997	-1.788437	-1.965124
C	-0.213317	0.616715	-4.144492
H	-0.793945	0.979041	-4.993471
C	1.246236	0.963040	-4.185051
H	1.735406	0.483831	-5.045096
H	1.382297	2.045484	-4.311259
H	1.783864	0.661386	-3.282585

INT4-p

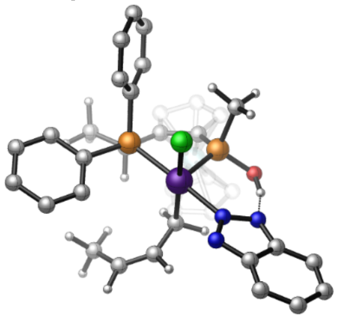

SMD(DCE)-B3LYP-D3/6-31G(d)/LanL2DZ
Thermal correction to Gibbs Free Energy = 0.569656
Sum of electronic and thermal Free Energies = -2971.015355
SMD(DCE)-B3LYP-D3/6-311++G(d,p)/SDD
Single point energy = -2973.6877362
Corrected quasi-harmonic Free Energy = -2973.126559
N_{imag} = 0

Cl	1.183238	-2.712889	1.790746
P	-1.404795	-1.261895	-0.093150
C	-1.195108	1.783961	1.029122
C	-2.269269	1.388847	0.137971
C	-2.334827	0.201487	-0.809368
H	-1.736192	0.423463	-1.695217
C	-1.640144	2.929269	1.778676
C	-3.349063	2.299716	0.370626
C	-2.963282	3.238707	1.370386
H	-3.556107	4.075746	1.715452
H	-4.294378	2.300139	-0.152377
H	-1.049198	3.469209	2.506512
C	-3.760136	-0.100729	-1.289455
H	-4.169300	0.778016	-1.799494
H	-4.426622	-0.362314	-0.461828
H	-3.758038	-0.920876	-2.010909
Fe	-1.661390	3.338707	-0.250199
C	-1.838394	5.278456	-0.956979
C	-0.489063	5.023343	-0.561579
C	0.001240	3.936223	-1.344544
C	-2.180553	4.347788	-1.985150
C	-1.043201	3.517336	-2.224627
H	-3.145760	4.259172	-2.467231
H	-0.992819	2.699141	-2.930853
H	0.974085	3.477109	-1.245895
H	0.051188	5.533073	0.225608
H	-2.501358	6.014243	-0.520032

C	-1.642660	-2.672877	-1.241728
C	-2.882074	-3.329937	-1.347513
C	-0.559562	-3.128293	-2.006839
C	-3.035152	-4.401375	-2.226781
H	-3.721804	-3.015206	-0.736490
C	-0.715492	-4.203436	-2.884755
H	0.404089	-2.639277	-1.926848
C	-1.953760	-4.837659	-2.999497
H	-3.997465	-4.899790	-2.303748
H	0.132563	-4.542862	-3.472899
H	-2.075890	-5.674930	-3.681233
C	-2.310470	-1.790466	1.417384
C	-2.130726	-3.109647	1.870743
C	-3.144045	-0.929983	2.151093
C	-2.770084	-3.554195	3.027356
H	-1.487311	-3.788035	1.324077
C	-3.781023	-1.380282	3.310403
H	-3.310012	0.089960	1.831445
C	-3.594925	-2.690638	3.753292
H	-2.619021	-4.576806	3.361954
H	-4.425168	-0.700976	3.862120
H	-4.090811	-3.038557	4.655454
P	0.349021	0.944657	1.410924
C	0.179395	0.416553	3.147579
H	-0.045281	1.291626	3.767500
H	-0.622617	-0.319927	3.235363
H	1.120399	-0.039934	3.464689
O	1.462808	2.110532	1.412559
H	2.387827	1.690749	1.220426
Rh	0.897069	-0.773517	0.121618
H	2.627536	-0.545692	-2.467417
C	5.078032	-0.754156	0.008015
C	4.884957	0.507929	0.629520
C	5.976423	1.331075	0.975709
C	7.240285	0.852142	0.678249
C	7.437061	-0.412305	0.053536
C	6.373091	-1.228378	-0.288391
H	5.826062	2.294902	1.453181
H	8.111178	1.452973	0.926568
H	8.452175	-0.738904	-0.155635
H	6.520703	-2.194267	-0.762699
N	3.543695	0.671552	0.775028
N	2.985173	-0.433794	0.272357
N	3.848630	-1.311130	-0.199580
C	1.592726	-0.232772	-2.603395
C	1.033947	0.582334	-1.507063
H	0.084139	1.059069	-1.731367
H	1.744379	1.340367	-1.171117
C	0.978467	-0.639822	-3.734473
H	1.557983	-1.257436	-4.421638
C	-0.422555	-0.345944	-4.181312
H	-0.428073	0.015052	-5.218678
H	-1.035709	-1.258006	-4.163175
H	-0.921040	0.405081	-3.562896

Rh-L2

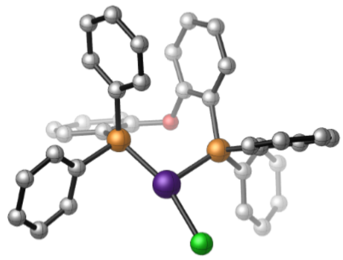

SMD(DCE)-B3LYP-D3/6-31G(d)/LanL2DZ
Thermal correction to Gibbs Free Energy = 0.464953
Sum of electronic and thermal Free Energies = -2716.078485
SMD(DCE)-B3LYP-D3/6-311++G(d,p)/SDD
Single point energy = -2718.0942868
Corrected quasi-harmonic Free Energy = -2717.634186
N_{imag} = 0

Rh	0.045429	-1.286047	-0.920746
P	-1.700836	-0.149196	-0.091797
P	1.689579	-0.071527	-0.078196
C	-2.202548	1.490984	-0.776732
C	-1.985881	0.005318	1.717652
C	-3.010985	-1.297052	-0.685079
C	1.238354	1.201979	1.173039
C	2.939534	-1.083741	0.825977
C	2.684954	0.863922	-1.319601
C	-1.252155	2.515903	-0.935267
O	0.068241	2.315193	-0.564579
C	0.370066	2.233705	0.786507
C	-3.515140	1.747854	-1.206839
C	-3.858860	2.970326	-1.787350
C	-2.889087	3.960865	-1.956066
C	-1.580617	3.733715	-1.526290
C	1.608847	1.124695	2.522507
C	1.103498	2.033275	3.454994
C	0.224689	3.038575	3.046471
C	-0.140876	3.148681	1.703735
C	2.521883	-2.251595	1.482996
C	3.427395	-3.003443	2.233500
C	4.762316	-2.600556	2.328262
C	5.187112	-1.442441	1.671854
C	4.281370	-0.684845	0.925963
C	3.248515	2.114884	-1.021120
C	4.014673	2.788882	-1.974402
C	4.227588	2.221567	-3.233467
C	3.673125	0.974706	-3.534539
C	2.903740	0.298552	-2.585486
C	-2.961222	0.863359	2.249930
C	-3.189761	0.896417	3.626202
C	-2.447916	0.075466	4.481333
C	-1.473642	-0.777721	3.957661
C	-1.241565	-0.811395	2.581301
C	-3.797267	-2.063733	0.189159
C	-4.685135	-3.014805	-0.317667
C	-4.794499	-3.214104	-1.696877
C	-4.014317	-2.455749	-2.574554
C	-3.125398	-1.503287	-2.074634
H	-4.273509	0.979631	-1.096481
H	-4.880834	3.142741	-2.112132
H	2.276544	0.336595	2.853345
H	1.388745	1.947481	4.499304
H	-0.178205	3.741549	3.769918
H	-3.539561	1.506831	1.593588

H	-3.945656	1.564160	4.030728
H	-2.626765	0.104820	5.552840
H	-0.888356	-1.411286	4.618520
H	-0.478037	-1.465892	2.170235
H	-3.719994	-1.919264	1.262129
H	-5.292651	-3.600055	0.367378
H	-5.485387	-3.956833	-2.086299
H	-4.096554	-2.604090	-3.647764
H	-2.526808	-0.910188	-2.761775
H	-3.147605	4.910382	-2.415859
H	4.820977	2.748892	-3.975736
H	4.442711	3.757983	-1.731757
H	3.090871	2.567692	-0.047072
H	2.475604	-0.670291	-2.823930
H	3.834925	0.526340	-4.511251
H	5.469528	-3.188761	2.907023
H	6.225060	-1.127207	1.737812
H	4.624690	0.214541	0.424563
H	1.488225	-2.572971	1.393541
H	3.091515	-3.906182	2.736648
H	-0.805879	4.485556	-1.638895
H	-0.818399	3.926689	1.367005
Cl	1.385218	-3.003672	-2.026703

INT13

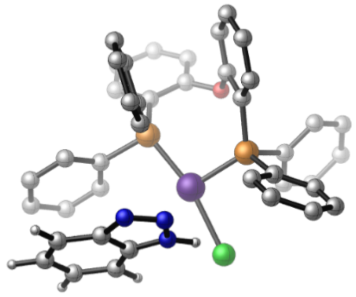

SMD(DCE)-B3LYP-D3/6-31G(d)/LanL2DZ
Thermal correction to Gibbs Free Energy = 0.564675
Sum of electronic and thermal Free Energies = -3111.886909
SMD(DCE)-B3LYP-D3/6-311++G(d,p)/SDD
Single point energy = -3114.1113477
Corrected quasi-harmonic Free Energy = -3113.552174
N_{imag} = 0

Rh	-0.058600	-0.416590	-1.144300
P	-0.679076	1.446045	-0.062974
P	1.882363	-0.757792	-0.129503
C	0.135817	3.091644	-0.296744
C	-0.995348	1.312651	1.742336
C	-2.340511	1.735903	-0.801910
C	2.273798	0.227603	1.378626
C	2.041236	-2.500307	0.447320
C	3.362982	-0.493567	-1.195757
C	1.535901	3.216801	-0.296583
O	2.335902	2.110313	-0.062812
C	2.395342	1.616876	1.232516
C	-0.617402	4.251676	-0.548209
C	0.002561	5.473531	-0.814998
C	1.396041	5.559274	-0.845217
C	2.166398	4.426110	-0.581092
C	2.380457	-0.309311	2.669493
C	2.578193	0.521468	3.774300
C	2.683562	1.903049	3.601180
C	2.600230	2.458228	2.323520

C	1.038890	-3.016432	1.285256
C	1.085120	-4.342807	1.712394
C	2.133607	-5.172922	1.302992
C	3.133779	-4.666409	0.470546
C	3.091287	-3.335921	0.043604
C	4.625267	-0.246020	-0.632654
C	5.738055	-0.061429	-1.454724
C	5.602398	-0.123988	-2.844745
C	4.349737	-0.375403	-3.409847
C	3.233374	-0.558709	-2.590441
C	-0.890291	2.391463	2.630113
C	-1.194577	2.215903	3.982681
C	-1.618496	0.970532	4.454128
C	-1.733458	-0.107551	3.571102
C	-1.414679	0.061921	2.224465
C	-3.520808	1.801955	-0.047251
C	-4.745592	2.018185	-0.682296
C	-4.805487	2.164073	-2.070690
C	-3.634395	2.089306	-2.830282
C	-2.407918	1.876247	-2.200815
H	-1.700847	4.197242	-0.546817
H	-0.604663	6.353160	-1.007800
H	2.296177	-1.379461	2.818766
H	2.644762	0.087451	4.767417
H	2.833332	2.552951	4.458533
H	-0.566475	3.364156	2.273185
H	-1.100356	3.054369	4.667535
H	-1.857318	0.840632	5.506308
H	-2.068147	-1.079483	3.922778
H	-1.489739	-0.777197	1.540822
H	-3.488514	1.687870	1.031266
H	-5.654779	2.067899	-0.089742
H	-5.761530	2.330260	-2.559701
H	-3.674150	2.199860	-3.910506
H	-1.496169	1.836936	-2.792966
H	1.884920	6.504058	-1.064768
H	6.469310	0.023981	-3.483223
H	6.709795	0.133007	-1.008535
H	4.742214	-0.193801	0.445895
H	2.260714	-0.757295	-3.029287
H	4.237671	-0.426410	-4.489611
H	2.167532	-6.209378	1.627885
H	3.950072	-5.306526	0.146324
H	3.872014	-2.960571	-0.609277
H	0.217704	-2.382869	1.602050
H	0.293716	-4.725291	2.350290
H	3.251281	4.461084	-0.585214
H	2.682831	3.529229	2.169803
Cl	0.070597	-2.494045	-2.508036
H	-1.164712	-3.199311	-0.447406
C	-4.029925	-2.374356	0.650779
C	-3.167137	-2.431570	-0.464984
C	-3.532506	-1.944228	-1.730883
C	-4.805218	-1.404298	-1.829140
C	-5.686549	-1.342732	-0.716617
C	-5.317880	-1.818937	0.530577
H	-2.848907	-1.992783	-2.571224
H	-5.139111	-1.007205	-2.782868
H	-6.670146	-0.902988	-0.853506
H	-5.985778	-1.771675	1.385014
N	-2.045906	-3.023519	0.032663
N	-2.192211	-3.304813	1.345775

N	-3.375818	-2.922796	1.733854

INT14

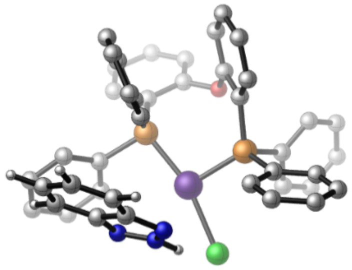

SMD(DCE)-B3LYP-D3/6-31G(d)/LanL2DZ
Thermal correction to Gibbs Free Energy = 0.565174
Sum of electronic and thermal Free Energies = -3111.883209
SMD(DCE)-B3LYP-D3/6-311++G(d,p)/SDD
Single point energy = -3114.1070497
Corrected quasi-harmonic Free Energy = -3113.547156
N_{imag} = 0

Rh	0.264362	-0.187129	-1.380118
P	-0.454266	1.555878	-0.167085
P	1.861941	-0.913992	-0.024815
C	0.530495	3.105936	0.051031
C	-1.249425	1.257812	1.460635
C	-1.827400	2.107130	-1.262274
C	1.900047	-0.216717	1.680222
C	1.763282	-2.729313	0.286322
C	3.580886	-0.664008	-0.645336
C	1.884484	3.060462	0.426388
O	2.502086	1.847333	0.679856
C	2.145236	1.156030	1.827605
C	-0.025622	4.367952	-0.220224
C	0.744235	5.529820	-0.140518
C	2.094853	5.451166	0.205586
C	2.666933	4.211153	0.493349
C	1.583392	-0.955721	2.828564
C	1.495021	-0.333635	4.075784
C	1.732134	1.037697	4.192036
C	2.067609	1.790078	3.065279
C	0.496111	-3.301405	0.474597
C	0.363811	-4.663702	0.740970
C	1.500706	-5.473253	0.819455
C	2.766269	-4.912097	0.631460
C	2.900577	-3.546462	0.366663
C	4.662665	-0.502029	0.234862
C	5.957592	-0.343390	-0.262194
C	6.186001	-0.346274	-1.641013
C	5.113790	-0.511064	-2.521601
C	3.816606	-0.667709	-2.028363
C	-1.386815	2.253025	2.438821
C	-2.071425	1.977999	3.624717
C	-2.629790	0.714458	3.837720
C	-2.496661	-0.280971	2.865463
C	-1.803280	-0.012182	1.685418
C	-3.148663	2.266994	-0.821041
C	-4.148033	2.636322	-1.724294
C	-3.843343	2.834172	-3.073835
C	-2.529311	2.672989	-3.521881
C	-1.524364	2.316180	-2.621984
H	-1.068974	4.441445	-0.508864
H	0.289132	6.491777	-0.357738
H	1.386427	-2.019145	2.747626

H	1.234609	-0.919862	4.952064
H	1.657883	1.526647	5.159140
H	-0.960017	3.238772	2.279907
H	-2.169264	2.752232	4.381054
H	-3.165306	0.505711	4.760102
H	-2.933436	-1.263379	3.020199
H	-1.690811	-0.782971	0.929074
H	-3.400179	2.102782	0.221465
H	-5.167681	2.766750	-1.371605
H	-4.626516	3.114630	-3.772800
H	-2.284506	2.828383	-4.569016
H	-0.500035	2.209265	-2.972943
H	2.702466	6.350028	0.257358
H	7.194328	-0.220086	-2.026343
H	6.786678	-0.216177	0.428956
H	4.497916	-0.497769	1.308159
H	2.986509	-0.798232	-2.715665
H	5.284044	-0.515419	-3.594971
H	1.400790	-6.536670	1.020448
H	3.653761	-5.537051	0.686236
H	3.890623	-3.127376	0.220139
H	-0.389229	-2.680792	0.403912
H	-0.627688	-5.088025	0.871633
H	3.711710	4.116466	0.771986
H	2.257781	2.856238	3.136113
Cl	0.490311	-1.959416	-3.084233
H	-1.501332	-2.519708	-2.038098
C	-3.788770	-2.675464	0.018134
C	-4.071684	-1.480258	-0.709130
C	-5.175659	-0.663838	-0.370371
C	-5.949233	-1.071670	0.696358
C	-5.663296	-2.265557	1.427203
C	-4.597134	-3.080454	1.105908
H	-5.388381	0.242429	-0.926161
H	-6.804351	-0.472349	0.996427
H	-6.309799	-2.532884	2.258438
H	-4.378027	-3.989205	1.657873
N	-3.121612	-1.333578	-1.666076
N	-2.355658	-2.398185	-1.481737
N	-2.675511	-3.240203	-0.511636

TS13-15

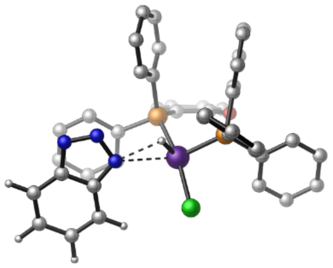

SMD(DCE)-B3LYP-D3/6-31G(d)/LanL2DZ
Thermal correction to Gibbs Free Energy = 0.562285
Sum of electronic and thermal Free Energies = -3111.846062
SMD(DCE)-B3LYP-D3/6-311++G(d,p)/SDD
Single point energy = -3114.0783499
Corrected quasi-harmonic Free Energy = -3113.522527
N_{imag} = 1, ν = -145.3160i cm^{-1}

Rh	0.220052	-0.764981	0.698196
P	0.621838	1.403390	-0.004843
P	-1.941800	-0.974786	0.072110
C	-0.107688	2.768893	0.996985

C	0.348038	1.838898	-1.757796
C	2.414180	1.566652	0.332374
C	-2.671457	0.407357	-0.885993
C	-2.120006	-2.439413	-1.011667
C	-3.122169	-1.163623	1.463109
C	-1.448485	2.746257	1.418145
O	-2.275928	1.694265	1.059156
C	-2.745454	1.652319	-0.245844
C	0.680451	3.864998	1.391327
C	0.156681	4.874593	2.198910
C	-1.167052	4.804110	2.638276
C	-1.974746	3.737292	2.243352
C	-3.165295	0.299584	-2.193370
C	-3.704380	1.414726	-2.837728
C	-3.768581	2.643536	-2.177155
C	-3.292918	2.768562	-0.870748
C	-1.360641	-2.498891	-2.193325
C	-1.468817	-3.599901	-3.040998
C	-2.324918	-4.655907	-2.712467
C	-3.073417	-4.604312	-1.535276
C	-2.974702	-3.500416	-0.684425
C	-4.501012	-1.086066	1.202030
C	-5.417344	-1.194783	2.247861
C	-4.966136	-1.366977	3.559897
C	-3.596250	-1.435848	3.822998
C	-2.673828	-1.336804	2.779118
C	-0.177786	3.083942	-2.131070
C	-0.365292	3.386815	-3.481025
C	-0.021676	2.456716	-4.464287
C	0.515866	1.220101	-4.095670
C	0.697369	0.905070	-2.748889
C	3.360692	1.795749	-0.674468
C	4.718190	1.842603	-0.352014
C	5.139484	1.662232	0.967254
C	4.197922	1.437665	1.975720
C	2.840948	1.388988	1.661763
H	1.714484	3.924487	1.070799
H	0.787141	5.709553	2.489377
H	-3.127086	-0.650997	-2.712832
H	-4.072970	1.320561	-3.854558
H	-4.188444	3.511344	-2.677363
H	-0.450205	3.813691	-1.376310
H	-0.782324	4.350221	-3.760808
H	-0.169172	2.695303	-5.514080
H	0.797397	0.496292	-4.855365
H	1.153727	-0.042785	-2.479737
H	3.048909	1.921858	-1.705198
H	5.447986	2.006646	-1.139349
H	6.198231	1.686105	1.208028
H	4.519324	1.291626	3.002862
H	2.111636	1.219679	2.450777
H	-1.575337	5.580285	3.278828
H	-5.681435	-1.444758	4.374170
H	-6.481844	-1.139295	2.037646
H	-4.859212	-0.939749	0.187171
H	-1.612016	-1.405502	2.987308
H	-3.241239	-1.567050	4.841380
H	-2.403003	-5.517047	-3.370354
H	-3.735098	-5.424748	-1.271909
H	-3.557932	-3.476447	0.229618
H	-0.692199	-1.684319	-2.456239
H	-0.879847	-3.634499	-3.953072

H	-3.011063	3.660798	2.556206
H	-3.333051	3.717190	-0.346014
Cl	0.197360	-3.006113	1.655413
H	0.609847	-1.240186	-0.680217
C	4.858894	-1.598917	-0.716624
C	3.754236	-1.818775	0.146758
C	3.936848	-2.163362	1.500668
C	5.241463	-2.263440	1.959872
C	6.351876	-2.031809	1.102280
C	6.179793	-1.703496	-0.234463
H	3.083713	-2.348844	2.147680
H	5.426194	-2.526770	2.998709
H	7.356450	-2.120267	1.509082
H	7.029780	-1.529294	-0.889490
N	2.632158	-1.617400	-0.603407
N	3.036226	-1.292976	-1.838943
N	4.363155	-1.275008	-1.950536

TS14-16

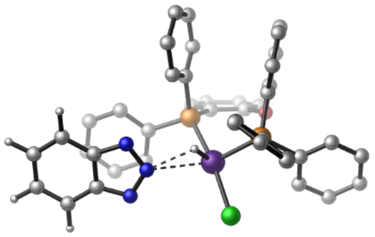

SMD(DCE)-B3LYP-D3/6-31G(d)/LanL2DZ
Thermal correction to Gibbs Free Energy = 0.562550
Sum of electronic and thermal Free Energies = -3111.844413
SMD(DCE)-B3LYP-D3/6-311++G(d,p)/SDD
Single point energy = -3114.0754811
Corrected quasi-harmonic Free Energy = -3113.519449
N_{imag} = 1, ν = -130.9869i cm^{-1}

Rh	0.086190	-0.707129	-1.050414
P	-0.722823	1.218445	-0.009958
P	2.117006	-0.838976	-0.077499
C	-0.022109	2.799149	-0.663251
C	-0.676251	1.335338	1.813588
C	-2.487338	1.350147	-0.486404
C	2.539622	0.416562	1.187840
C	2.263186	-2.449471	0.777463
C	3.531659	-0.691833	-1.236121
C	1.355945	2.966010	-0.892347
O	2.247875	1.950371	-0.583327
C	2.561481	1.748125	0.752415
C	-0.867858	3.872292	-0.998159
C	-0.361057	5.043104	-1.562659
C	1.006444	5.163640	-1.816986
C	1.869073	4.121386	-1.476960
C	2.884793	0.142195	2.517900
C	3.229737	1.182683	3.382879
C	3.247099	2.501681	2.923120
C	2.917120	2.793486	1.598593
C	1.385761	-2.722802	1.841210
C	1.444419	-3.949266	2.500760
C	2.367820	-4.918386	2.096054
C	3.231129	-4.655407	1.030813
C	3.181643	-3.425761	0.369513
C	4.837098	-0.715483	-0.716377
C	5.930625	-0.559098	-1.568043
C	5.729240	-0.360686	-2.937276

C	4.431968	-0.320516	-3.453430
C	3.334098	-0.487030	-2.607029
C	-0.321503	2.522880	2.468162
C	-0.316328	2.576782	3.863592
C	-0.674716	1.453254	4.611161
C	-1.047601	0.273317	3.960279
C	-1.047012	0.207981	2.566789
C	-3.507148	1.552262	0.452433
C	-4.830006	1.690965	0.026289
C	-5.142209	1.629318	-1.332992
C	-4.128305	1.420130	-2.272996
C	-2.806850	1.278765	-1.853886
H	-1.934743	3.789598	-0.824513
H	-1.038705	5.854321	-1.811157
H	2.882739	-0.878964	2.881245
H	3.484864	0.960348	4.414451
H	3.517477	3.310438	3.595707
H	-0.041493	3.401808	1.897415
H	-0.029092	3.497812	4.363174
H	-0.667868	1.497637	5.696879
H	-1.341152	-0.600235	4.535896
H	-1.382759	-0.697782	2.070123
H	-3.276527	1.598487	1.511133
H	-5.616121	1.838626	0.760639
H	-6.173227	1.730334	-1.659146
H	-4.366718	1.359659	-3.331068
H	-2.021506	1.127532	-2.591056
H	1.404532	6.066424	-2.270729
H	6.581908	-0.233850	-3.598760
H	6.937585	-0.586878	-1.161130
H	4.999981	-0.855237	0.348729
H	2.329519	-0.462521	-3.013032
H	4.270664	-0.160249	-4.515815
H	2.408992	-5.876663	2.606398
H	3.944000	-5.408283	0.706439
H	3.851211	-3.239389	-0.462764
H	0.662103	-1.977361	2.156530
H	0.765216	-4.148807	3.324678
H	2.938502	4.187329	-1.649489
H	2.923375	3.812987	1.227788
Cl	0.642571	-2.656179	-2.406799
H	-0.465487	-1.510501	0.104912
C	-4.372219	-1.775338	0.517439
C	-4.473518	-1.932788	-0.892215
C	-5.727136	-1.902019	-1.537958
C	-6.848628	-1.704181	-0.748333
C	-6.747124	-1.542567	0.662129
C	-5.522431	-1.579284	1.310179
H	-5.804992	-2.019857	-2.615817
H	-7.832321	-1.669217	-1.210412
H	-7.656000	-1.387411	1.238617
H	-5.445372	-1.452364	2.387178
N	-3.207524	-2.070650	-1.381518
N	-2.403995	-2.003035	-0.316696
N	-3.048803	-1.824051	0.839155

INT15

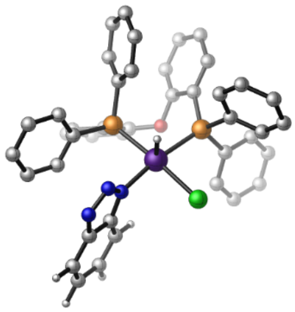

SMD(DCE)-B3LYP-D3/6-31G(d)/LanL2DZ
Thermal correction to Gibbs Free Energy = 0.564829
Sum of electronic and thermal Free Energies = -3111.877945
SMD(DCE)-B3LYP-D3/6-311++G(d,p)/SDD
Single point energy = -3114.1133245
Corrected quasi-harmonic Free Energy = -3113.555277
N_{imag} = 0

Rh	-0.328737	-0.937924	0.450290
P	-0.991768	1.287753	0.052637
P	2.009045	-0.720149	0.101387
C	-1.036834	1.776180	-1.732403
C	0.015010	2.567169	0.896899
C	-2.708739	1.609142	0.631944
C	2.693079	0.910040	-0.392111
C	2.955937	-1.199071	1.595851
C	2.641026	-1.769871	-1.272563
C	-0.001359	1.409708	-2.611684
O	1.111035	0.737770	-2.131922
C	2.116666	1.486945	-1.531635
C	-2.110232	2.506252	-2.274580
C	-2.170196	2.802633	-3.636807
C	-1.158266	2.369414	-4.494942
C	-0.064489	1.675382	-3.977509
C	3.741997	1.588998	0.239492
C	4.186420	2.818090	-0.253835
C	3.594413	3.371194	-1.391295
C	2.554058	2.705156	-2.042698
C	2.844420	-0.402263	2.747744
C	3.503557	-0.764020	3.922425
C	4.268668	-1.933303	3.965257
C	4.373343	-2.734923	2.826968
C	3.720516	-2.372609	1.646041
C	3.997366	-1.692204	-1.630448
C	4.485727	-2.438275	-2.703191
C	3.622001	-3.256242	-3.438000
C	2.270472	-3.328327	-3.093558
C	1.781077	-2.590694	-2.013322
C	0.329441	3.783675	0.275709
C	1.057439	4.755034	0.965693
C	1.462405	4.528043	2.282694
C	1.135181	3.324509	2.913685
C	0.418542	2.346431	2.223722
C	-2.961865	2.362786	1.785924
C	-4.271537	2.525953	2.246149
C	-5.336892	1.941042	1.561716
C	-5.091031	1.192867	0.406300
C	-3.787743	1.026587	-0.055312
H	-2.913660	2.842579	-1.630767
H	-3.014293	3.365441	-4.023817
H	4.209869	1.163703	1.120500
H	4.994582	3.339335	0.250384

H	3.939791	4.325816	-1.777641
H	0.019030	3.972390	-0.746532
H	1.309433	5.687930	0.469240
H	2.030000	5.285636	2.815946
H	1.440466	3.142326	3.940214
H	0.162040	1.417250	2.723259
H	-2.149333	2.824511	2.335233
H	-4.452607	3.110996	3.143613
H	-6.353355	2.063602	1.925398
H	-5.911777	0.726285	-0.130831
H	-3.613817	0.438096	-0.949262
H	-1.209009	2.581814	-5.558747
H	4.001878	-3.833463	-4.276710
H	5.537972	-2.376782	-2.967151
H	4.671318	-1.048481	-1.072171
H	0.735487	-2.667589	-1.739093
H	1.594118	-3.961044	-3.661802
H	4.777572	-2.218297	4.882059
H	4.961090	-3.648389	2.854208
H	3.805585	-3.009922	0.772912
H	2.253077	0.506865	2.726272
H	3.415027	-0.134519	4.803681
H	0.753880	1.346810	-4.610076
H	2.084661	3.127063	-2.924688
Cl	0.178135	-3.289857	0.928648
H	-0.119105	-0.540602	1.894919
C	-4.326509	-2.319905	0.814779
C	-3.166961	-2.083660	0.040176
C	-3.093118	-2.449340	-1.317379
C	-4.211581	-3.060291	-1.863776
C	-5.379265	-3.306399	-1.090810
C	-5.454167	-2.943870	0.245321
H	-2.203877	-2.255079	-1.911565
H	-4.200627	-3.359973	-2.908519
H	-6.229681	-3.788798	-1.565471
H	-6.348135	-3.125521	0.835637
N	-2.299881	-1.444133	0.873861
N	-2.883755	-1.307829	2.075520
N	-4.100367	-1.820878	2.072983

INT16

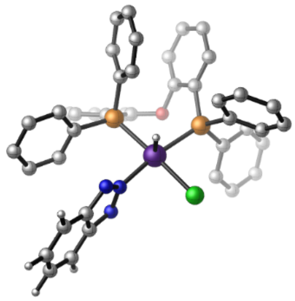

SMD(DCE)-B3LYP-D3/6-31G(d)/LanL2DZ
Thermal correction to Gibbs Free Energy = 0.565800
Sum of electronic and thermal Free Energies = -3111.879176
SMD(DCE)-B3LYP-D3/6-311++G(d,p)/SDD
Single point energy = -3114.1145607
Corrected quasi-harmonic Free Energy = -3113.555806
N_{imag} = 0

Rh	-0.340999	-0.985545	-0.033148
P	-0.804224	1.329234	0.096912
P	2.035983	-0.889092	-0.002596
C	-0.504214	2.319689	-1.438414

C	0.080832	2.206145	1.443937
C	-2.584790	1.631735	0.450669
C	2.864388	0.742764	0.127900
C	2.730741	-1.853884	1.394877
C	2.809153	-1.556604	-1.533991
C	0.646471	2.127823	-2.225481
O	1.630219	1.239877	-1.819558
C	2.536964	1.678040	-0.861984
C	-1.431596	3.279848	-1.883167
C	-1.243533	3.970553	-3.080763
C	-0.122686	3.712126	-3.871164
C	0.829938	2.790834	-3.436710
C	3.795276	1.107065	1.108476
C	4.372012	2.379230	1.096418
C	4.033475	3.291173	0.094437
C	3.113643	2.944353	-0.897295
C	2.354797	-1.499209	2.701050
C	2.842823	-2.213825	3.794508
C	3.700526	-3.299905	3.595320
C	4.069606	-3.662885	2.298767
C	3.589654	-2.943778	1.200972
C	4.183855	-1.367402	-1.757200
C	4.778058	-1.835987	-2.929035
C	4.004424	-2.486018	-3.895278
C	2.636578	-2.669489	-3.682531
C	2.039501	-2.209864	-2.506216
C	0.579946	3.504530	1.272791
C	1.212435	4.158098	2.332568
C	1.338412	3.530022	3.573194
C	0.825082	2.242895	3.755841
C	0.202736	1.582264	2.696634
C	-3.023089	2.081627	1.703026
C	-4.389629	2.227444	1.958431
C	-5.327146	1.927165	0.970007
C	-4.894875	1.483582	-0.283617
C	-3.534470	1.336412	-0.542683
H	-2.315919	3.487798	-1.293498
H	-1.980514	4.702983	-3.396053
H	4.065665	0.401100	1.885969
H	5.084864	2.654228	1.867934
H	4.483439	4.279737	0.080975
H	0.489610	4.003723	0.314051
H	1.609623	5.158216	2.183459
H	1.833431	4.040588	4.394585
H	0.912355	1.748481	4.719218
H	-0.201590	0.586453	2.850350
H	-2.311982	2.317959	2.486462
H	-4.715955	2.574702	2.934933
H	-6.389173	2.032645	1.173841
H	-5.616682	1.237020	-1.056858
H	-3.213185	0.975557	-1.513208
H	0.020104	4.233293	-4.813180
H	4.466852	-2.845631	-4.810572
H	5.842752	-1.688577	-3.088713
H	4.790430	-0.852986	-1.017437
H	0.980183	-2.373153	-2.343924
H	2.029302	-3.171898	-4.430474
H	4.075330	-3.861522	4.446714
H	4.730607	-4.509794	2.135871
H	3.881467	-3.241336	0.199740
H	1.688297	-0.659403	2.865480
H	2.548414	-1.925471	4.799964

H	1.726433	2.583707	-4.012107
H	2.840770	3.647039	-1.677028
Cl	-0.032252	-3.422376	0.018280
H	-0.319979	-0.972572	1.479274
C	-4.348354	-1.891616	0.571333
C	-4.235827	-1.981786	-0.843447
C	-5.346936	-2.325684	-1.642643
C	-6.542949	-2.572682	-0.990659
C	-6.654889	-2.485666	0.426574
C	-5.573479	-2.148664	1.222055
H	-5.261833	-2.390750	-2.724013
H	-7.423419	-2.840641	-1.569225
H	-7.617744	-2.689052	0.888319
H	-5.659500	-2.076042	2.302687
N	-2.954086	-1.661355	-1.174623
N	-2.366102	-1.399473	-0.011890
N	-3.133317	-1.519239	1.062806

INT17

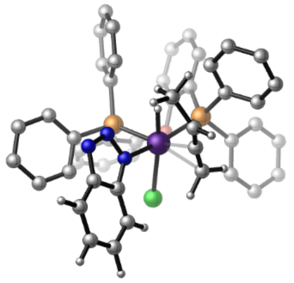

SMD(DCE)-B3LYP-D3/6-31G(d)/LanL2DZ
Thermal correction to Gibbs Free Energy = 0.648112
Sum of electronic and thermal Free Energies = -3267.790686
SMD(DCE)-B3LYP-D3/6-311++G(d,p)/SDD
Single point energy = -3270.1553139
Corrected quasi-harmonic Free Energy = -3269.515941
N_{imag} = 0

Rh	-0.367020	-0.793613	-0.254973
P	-0.791390	1.485269	0.230187
P	2.019092	-0.725457	-0.172184
C	-0.308601	2.764413	-1.018556
C	-0.078571	2.028841	1.832717
C	-2.594661	1.804704	0.390710
C	2.862011	0.781165	0.457146
C	2.519069	-2.044203	1.014086
C	3.023041	-1.085033	-1.675180
C	0.914142	2.729825	-1.709068
O	1.848168	1.751422	-1.419629
C	2.655156	1.933328	-0.310217
C	-1.197795	3.804064	-1.344986
C	-0.892862	4.736794	-2.337598
C	0.308970	4.645794	-3.040041
C	1.218408	3.638052	-2.719387
C	3.716080	0.861742	1.564338
C	4.321114	2.075593	1.898860
C	4.091200	3.212994	1.120867
C	3.257936	3.148628	0.002808
C	2.199533	-1.895249	2.375234
C	2.508014	-2.902363	3.288019
C	3.110389	-4.086506	2.850881
C	3.411624	-4.250426	1.498078
C	3.125654	-3.232787	0.583499
C	4.425972	-1.061896	-1.576333

C	5.212933	-1.346006	-2.691492
C	4.609519	-1.649892	-3.916677
C	3.217929	-1.665273	-4.020177
C	2.423744	-1.383690	-2.904257
C	0.549482	3.271259	1.987607
C	1.070578	3.643870	3.229606
C	0.954206	2.788572	4.326230
C	0.300935	1.559763	4.184322
C	-0.211982	1.179397	2.945054
C	-3.144370	2.402847	1.530858
C	-4.520852	2.636857	1.602138
C	-5.350844	2.275310	0.540399
C	-4.801093	1.687216	-0.604024
C	-3.430135	1.455562	-0.683276
H	-2.145934	3.885787	-0.828151
H	-1.603901	5.525477	-2.564796
H	3.912256	-0.017324	2.167240
H	4.974274	2.128853	2.764710
H	4.564210	4.155951	1.379870
H	0.645732	3.945180	1.143451
H	1.572198	4.601865	3.333741
H	1.364344	3.079225	5.289539
H	0.190726	0.895909	5.037380
H	-0.736578	0.234369	2.843484
H	-2.512498	2.681777	2.367027
H	-4.940492	3.096170	2.493049
H	-6.421836	2.448942	0.602055
H	-5.441563	1.396382	-1.432125
H	-3.004713	0.989985	-1.566350
H	0.544587	5.356709	-3.826584
H	5.224123	-1.868565	-4.785913
H	6.295952	-1.327913	-2.604647
H	4.903789	-0.826387	-0.629527
H	1.343890	-1.363165	-2.996916
H	2.742246	-1.889500	-4.971209
H	3.339218	-4.876304	3.561086
H	3.873856	-5.169365	1.147967
H	3.371624	-3.376303	-0.463113
H	1.706956	-0.991941	2.723428
H	2.264445	-2.767629	4.338241
H	2.168849	3.542740	-3.234652
H	3.071246	4.023319	-0.610733
Cl	-0.833776	-0.198139	-2.746159
H	-0.166054	-0.983060	1.260733
C	-4.499058	-1.895762	0.322846
C	-3.425219	-1.756275	-0.591412
C	-3.571109	-2.132303	-1.942121
C	-4.799705	-2.647127	-2.327606
C	-5.875673	-2.792704	-1.411492
C	-5.741681	-2.419535	-0.084109
H	-2.764717	-1.989192	-2.650551
H	-4.948472	-2.942829	-3.363245
H	-6.819598	-3.198820	-1.765907
H	-6.559853	-2.518117	0.624201
N	-2.402454	-1.210154	0.135479
N	-2.833970	-1.037197	1.400300
N	-4.080620	-1.431810	1.543382
C	-0.399474	-3.311915	0.211351
C	-0.123991	-3.019149	-1.067068
H	-0.911729	-2.979346	-1.814093
H	0.894659	-3.092481	-1.441674
C	-0.672808	-3.876914	1.359128

C	-0.948674	-3.277934	2.707026
H	-1.991242	-3.465392	2.991246
H	-0.308125	-3.750379	3.460040
H	-0.780924	-2.201465	2.714313
H	-0.713277	-4.968541	1.306336

INT18

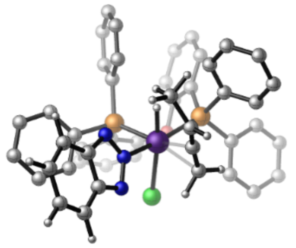

SMD(DCE)-B3LYP-D3/6-31G(d)/LanL2DZ
Thermal correction to Gibbs Free Energy = 0.646725
Sum of electronic and thermal Free Energies = -3267.791867
SMD(DCE)-B3LYP-D3/6-311++G(d,p)/SDD
Single point energy = -3270.1544352
Corrected quasi-harmonic Free Energy = -3269.516038
N_{imag} = 0

Rh	-0.294214	-0.720260	-0.532806
P	-0.658817	1.493707	0.213639
P	2.055359	-0.828891	-0.127981
C	0.042742	2.885871	-0.784634
C	-0.122540	1.797994	1.943506
C	-2.451024	1.897260	0.200308
C	2.881448	0.523614	0.803204
C	2.316829	-2.316861	0.925193
C	3.237281	-1.061953	-1.522318
C	1.345210	2.861661	-1.309788
O	2.186821	1.798258	-1.036815
C	2.837870	1.777840	0.183277
C	-0.746683	4.010933	-1.081045
C	-0.268291	5.040623	-1.892893
C	1.012184	4.963791	-2.441027
C	1.823618	3.869021	-2.143080
C	3.584341	0.400372	2.008511
C	4.197656	1.516318	2.582908
C	4.130376	2.759098	1.948225
C	3.453412	2.897874	0.735534
C	1.828032	-2.312700	2.243210
C	1.961273	-3.441167	3.050315
C	2.554343	-4.601578	2.542367
C	3.020930	-4.620689	1.227041
C	2.910456	-3.482961	0.422391
C	4.609323	-1.172677	-1.232770
C	5.531656	-1.359424	-2.261100
C	5.095736	-1.431152	-3.588565
C	3.736246	-1.310882	-3.880275
C	2.806027	-1.126036	-2.852467
C	0.490656	2.998418	2.326260
C	0.869311	3.201775	3.655607
C	0.625049	2.217959	4.614999
C	-0.010406	1.028540	4.244356
C	-0.380902	0.817611	2.916760
C	-3.119883	2.349749	1.343895
C	-4.486703	2.637988	1.284222
C	-5.187125	2.476056	0.088164
C	-4.516679	2.033956	-1.057881

C	-3.154213	1.749155	-1.007210
H	-1.752130	4.083117	-0.685229
H	-0.905658	5.894196	-2.103774
H	3.653671	-0.560862	2.504297
H	4.730234	1.411066	3.523353
H	4.609633	3.626000	2.394044
H	0.686269	3.771113	1.590794
H	1.361435	4.129048	3.935310
H	0.924999	2.376602	5.647226
H	-0.214974	0.262169	4.986814
H	-0.881449	-0.104317	2.638621
H	-2.588792	2.472565	2.281697
H	-5.000773	2.984115	2.176892
H	-6.251682	2.690410	0.047083
H	-5.057777	1.900037	-1.990406
H	-2.635728	1.389473	-1.890224
H	1.383910	5.751846	-3.089408
H	5.815801	-1.573881	-4.389993
H	6.589212	-1.445832	-2.026549
H	4.957468	-1.114896	-0.205453
H	1.755356	-0.995986	-3.086531
H	3.392486	-1.353171	-4.910361
H	2.647744	-5.485024	3.168007
H	3.476517	-5.520005	0.821607
H	3.285380	-3.514396	-0.594937
H	1.341689	-1.426110	2.639229
H	1.589272	-3.418166	4.070857
H	2.829591	3.780286	-2.540460
H	3.397301	3.855195	0.228897
Cl	-0.382938	0.240593	-2.947656
H	-0.328494	-1.133699	0.953444
C	-4.311170	-1.431030	0.276362
C	-4.317553	-1.683470	-1.123011
C	-5.498739	-2.084514	-1.784840
C	-6.640454	-2.221281	-1.014572
C	-6.632696	-1.970161	0.388334
C	-5.483089	-1.576380	1.050229
H	-5.505664	-2.272534	-2.855086
H	-7.571747	-2.525994	-1.485323
H	-7.558513	-2.090557	0.945370
H	-5.477176	-1.376774	2.118361
N	-3.062688	-1.447990	-1.592961
N	-2.369888	-1.071231	-0.523664
N	-3.052375	-1.053116	0.620383
C	-0.535248	-3.268539	-0.477657
C	-0.080704	-2.801089	-1.649034
H	-0.769289	-2.552113	-2.452633
H	0.970602	-2.901955	-1.910255
C	-0.993443	-3.973520	0.524467
C	-1.394527	-3.562713	1.911130
H	-2.460227	-3.770186	2.066074
H	-0.833730	-4.148898	2.648016
H	-1.220232	-2.501282	2.086326
H	-1.102610	-5.037095	0.294393

TS17-19

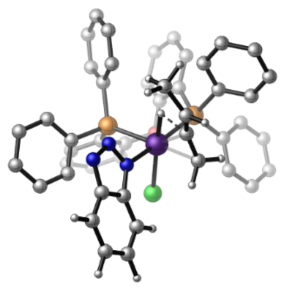

SMD(DCE)-B3LYP-D3/6-31G(d)/LanL2DZ
Thermal correction to Gibbs Free Energy = 0.647276
Sum of electronic and thermal Free Energies = -3267.780158
SMD(DCE)-B3LYP-D3/6-311++G(d,p)/SDD
Single point energy = -3270.1466442
Corrected quasi-harmonic Free Energy = -3269.50804
N_{imag} = 1, ν = -709.1948i cm^{-1}

Rh	-0.366786	-0.900041	-0.192773
P	-0.818900	1.499827	0.208543
P	2.029270	-0.724843	-0.210477
C	-0.454161	2.713818	-1.145403
C	-0.031636	2.184200	1.719011
C	-2.616874	1.798090	0.449753
C	2.833941	0.869089	0.227128
C	2.668600	-1.879002	1.074457
C	2.936783	-1.215988	-1.733553
C	0.699771	2.617126	-1.940119
O	1.667562	1.671104	-1.644461
C	2.527279	1.954757	-0.601146
C	-1.366379	3.733943	-1.469243
C	-1.152953	4.583726	-2.556077
C	-0.023182	4.424512	-3.359404
C	0.911413	3.437974	-3.044465
C	3.754052	1.067379	1.266431
C	4.318635	2.326014	1.481850
C	3.983000	3.396477	0.649681
C	3.087319	3.215411	-0.404415
C	2.400835	-1.605301	2.427610
C	2.818234	-2.486553	3.422487
C	3.483611	-3.668191	3.078685
C	3.738793	-3.954174	1.736857
C	3.340671	-3.061196	0.736957
C	4.310024	-0.931506	-1.834241
C	5.033993	-1.342954	-2.952850
C	4.396716	-2.042991	-3.982000
C	3.033518	-2.327987	-3.886487
C	2.303139	-1.917293	-2.767904
C	0.499312	3.478998	1.785955
C	1.080169	3.937900	2.970994
C	1.120131	3.116970	4.100118
C	0.572700	1.831267	4.045396
C	0.005244	1.366019	2.859495
C	-3.138969	2.382604	1.608737
C	-4.519321	2.567905	1.738006
C	-5.380360	2.171922	0.714121
C	-4.859486	1.595630	-0.450335
C	-3.486057	1.410257	-0.584507
H	-2.263513	3.862727	-0.875533
H	-1.879211	5.360274	-2.777525
H	4.033949	0.243971	1.912371
H	5.021594	2.465746	2.297533
H	4.421565	4.376387	0.815349

H	0.471928	4.125957	0.915479
H	1.503754	4.937795	3.007717
H	1.574579	3.477007	5.019123
H	0.592286	1.188468	4.921305
H	-0.420847	0.369142	2.815809
H	-2.480866	2.688724	2.415303
H	-4.917405	3.017847	2.643620
H	-6.453227	2.308752	0.820459
H	-5.524488	1.278558	-1.248980
H	-3.082606	0.951735	-1.482258
H	0.139076	5.068702	-4.218725
H	4.960915	-2.360406	-4.854910
H	6.094391	-1.115337	-3.020238
H	4.815974	-0.390117	-1.040551
H	1.240451	-2.115691	-2.717385
H	2.529915	-2.864606	-4.685997
H	3.799988	-4.360611	3.853951
H	4.253928	-4.870549	1.461964
H	3.553282	-3.293693	-0.301326
H	1.863598	-0.702816	2.703725
H	2.613174	-2.256584	4.464273
H	1.811851	3.296252	-3.633572
H	2.820238	4.035948	-1.061373
Cl	-0.758712	-0.403699	-2.616723
H	-0.165133	-1.345889	1.347758
C	-4.505200	-2.013352	0.273430
C	-3.398872	-1.902810	-0.604795
C	-3.469290	-2.384451	-1.927597
C	-4.666592	-2.958207	-2.327878
C	-5.781713	-3.060268	-1.453319
C	-5.717045	-2.592780	-0.150644
H	-2.625674	-2.286097	-2.599470
H	-4.759148	-3.338230	-3.342304
H	-6.699181	-3.514031	-1.819181
H	-6.563552	-2.665331	0.526806
N	-2.421777	-1.285104	0.125695
N	-2.899248	-1.062519	1.365787
N	-4.142192	-1.476331	1.483138
C	-0.304767	-2.884366	0.940657
C	-0.146530	-3.062640	-0.431547
H	-0.997482	-3.389872	-1.024831
H	0.829650	-3.336020	-0.826425
C	-0.542832	-3.410631	2.129229
C	-0.774654	-2.731110	3.440431
H	-1.801863	-2.910248	3.782049
H	-0.098706	-3.134850	4.203789
H	-0.623948	-1.651464	3.373007
H	-0.565340	-4.502156	2.133318

TS18-20

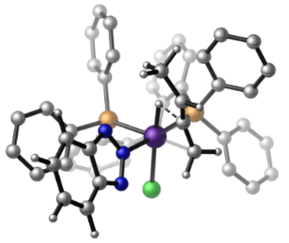

SMD(DCE)-B3LYP-D3/6-31G(d)/LanL2DZ
Thermal correction to Gibbs Free Energy = 0.645532
Sum of electronic and thermal Free Energies = -3267.780858
SMD(DCE)-B3LYP-D3/6-311++G(d,p)/SDD
Single point energy = -3270.1446103

Corrected quasi-harmonic Free Energy = -3269.507069
N_{imag} = 1, ν = -711.2037i cm^{-1}

Rh	-0.268674	-0.823342	-0.545432
P	-0.714295	1.468465	0.244459
P	2.108503	-0.774253	-0.190952
C	-0.128918	2.892429	-0.789159
C	-0.142284	1.829272	1.951335
C	-2.523149	1.795622	0.286445
C	2.874878	0.664355	0.659651
C	2.515496	-2.184793	0.920681
C	3.225620	-1.019689	-1.631977
C	1.129863	2.892539	-1.412392
O	2.025120	1.863719	-1.169384
C	2.722822	1.900891	0.021850
C	-0.963452	3.996386	-1.038722
C	-0.573705	5.024545	-1.898799
C	0.661532	4.966925	-2.545222
C	1.519786	3.896193	-2.295043
C	3.629769	0.615729	1.840141
C	4.184467	1.780421	2.374174
C	4.007186	3.003557	1.723304
C	3.278649	3.070672	0.535664
C	2.060115	-2.154342	2.250448
C	2.296119	-3.230350	3.103272
C	2.964454	-4.365307	2.631632
C	3.404203	-4.409663	1.307892
C	3.188786	-3.322098	0.455593
C	4.604896	-0.797249	-1.473646
C	5.482499	-1.038838	-2.530190
C	4.994500	-1.506316	-3.754359
C	3.625778	-1.727388	-3.916686
C	2.741562	-1.485583	-2.861642
C	0.389360	3.069335	2.329406
C	0.799405	3.284446	3.647643
C	0.666547	2.272296	4.600649
C	0.118637	1.038977	4.233815
C	-0.279352	0.817846	2.915567
C	-3.200910	2.160759	1.454970
C	-4.583610	2.370027	1.425456
C	-5.291795	2.218799	0.232736
C	-4.614001	1.863255	-0.939098
C	-3.237602	1.652637	-0.915466
H	-1.937116	4.052145	-0.566822
H	-1.244460	5.861400	-2.069851
H	3.786048	-0.327291	2.349960
H	4.756033	1.728438	3.295914
H	4.439575	3.910289	2.136656
H	0.494597	3.863276	1.597557
H	1.224530	4.244673	3.926624
H	0.988178	2.442464	5.624531
H	0.005799	0.248118	4.970456
H	-0.708358	-0.137042	2.630491
H	-2.663008	2.276966	2.390223
H	-5.103608	2.647349	2.338656
H	-6.367407	2.372480	0.213810
H	-5.160684	1.734703	-1.869171
H	-2.714620	1.353744	-1.818940
H	0.963449	5.753313	-3.230905
H	5.678863	-1.692939	-4.577611
H	6.546183	-0.861214	-2.396777
H	4.995984	-0.436898	-0.526998

H	1.678710	-1.628233	-3.007252
H	3.238321	-2.082507	-4.867747
H	3.139187	-5.209247	3.293311
H	3.921158	-5.288726	0.932813
H	3.542248	-3.370085	-0.569086
H	1.519302	-1.287767	2.618456
H	1.947789	-3.187584	4.131453
H	2.496028	3.826634	-2.764172
H	3.136809	4.011961	0.016067
Cl	-0.286567	0.110821	-2.862782
H	-0.317117	-1.576509	0.885771
C	-4.290006	-1.577350	0.152141
C	-4.286595	-1.763487	-1.257868
C	-5.460558	-2.141544	-1.945726
C	-6.604351	-2.327689	-1.189397
C	-6.606185	-2.145118	0.224325
C	-5.464837	-1.771609	0.911202
H	-5.459911	-2.276835	-3.023890
H	-7.530042	-2.618541	-1.679517
H	-7.533395	-2.302402	0.769719
H	-5.466970	-1.622663	1.987534
N	-3.031165	-1.494533	-1.709076
N	-2.350287	-1.160970	-0.619955
N	-3.037768	-1.203151	0.520805
C	-0.429587	-2.996168	0.160760
C	-0.080524	-2.905652	-1.183984
H	-0.852480	-3.024929	-1.941299
H	0.932657	-3.152949	-1.493193
C	-0.848138	-3.729773	1.176856
C	-1.246263	-3.306023	2.554050
H	-2.315942	-3.494334	2.711619
H	-0.696844	-3.884996	3.305982
H	-1.059715	-2.243461	2.724817
H	-0.903988	-4.798015	0.958917

INT19

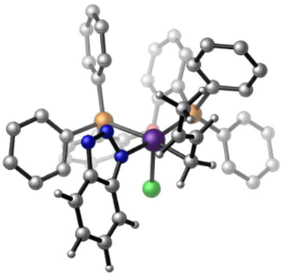

SMD(DCE)-B3LYP-D3/6-31G(d)/LanL2DZ
Thermal correction to Gibbs Free Energy = 0.650190
Sum of electronic and thermal Free Energies = -3267.811415
SMD(DCE)-B3LYP-D3/6-311++G(d,p)/SDD
Single point energy = -3270.1711773
Corrected quasi-harmonic Free Energy = -3269.528617
N_{imag} = 0

Rh	-0.260183	-0.882171	-0.325802
P	-0.816268	1.584602	0.260262
P	2.113491	-0.684748	-0.240738
C	-0.368497	2.917218	-0.949852
C	-0.202544	2.148092	1.895760
C	-2.640860	1.830438	0.358114
C	2.842728	0.877482	0.399159
C	2.556717	-1.890528	1.071998
C	3.207969	-1.086090	-1.659417

C	0.822890	2.865185	-1.693683
O	1.765014	1.881522	-1.433556
C	2.556842	2.047720	-0.313833
C	-1.253731	3.971178	-1.240156
C	-0.978277	4.899928	-2.245032
C	0.190976	4.789295	-2.999718
C	1.098758	3.768529	-2.717500
C	3.677466	0.969692	1.522503
C	4.190425	2.203811	1.926068
C	3.886759	3.357320	1.199006
C	3.070299	3.285872	0.070061
C	1.965360	-1.717375	2.337409
C	2.154216	-2.666635	3.340794
C	2.931134	-3.802675	3.091007
C	3.528039	-3.975710	1.839695
C	3.343291	-3.025046	0.831559
C	4.594169	-0.879505	-1.540839
C	5.447447	-1.214779	-2.591378
C	4.927353	-1.754379	-3.772298
C	3.551767	-1.955578	-3.897583
C	2.692453	-1.623723	-2.846489
C	0.375143	3.403104	2.124635
C	0.860637	3.731804	3.393340
C	0.758367	2.818745	4.445035
C	0.162201	1.571808	4.228907
C	-0.310484	1.234845	2.960530
C	-3.291402	2.319090	1.497848
C	-4.681938	2.466794	1.505637
C	-5.431953	2.129898	0.377832
C	-4.786622	1.644442	-0.764508
C	-3.401570	1.494119	-0.775096
H	-2.180266	4.060300	-0.684498
H	-1.684881	5.700783	-2.442547
H	3.922129	0.078976	2.089913
H	4.825658	2.261192	2.804865
H	4.284167	4.319446	1.509490
H	0.457407	4.120239	1.314384
H	1.320866	4.702703	3.556410
H	1.138163	3.077507	5.429765
H	0.069929	0.859683	5.044631
H	-0.784073	0.269563	2.795977
H	-2.722627	2.583498	2.383309
H	-5.175353	2.844860	2.397240
H	-6.513137	2.239619	0.387987
H	-5.362652	1.365501	-1.642762
H	-2.908735	1.101142	-1.659250
H	0.403396	5.496974	-3.795891
H	5.593198	-2.012371	-4.591433
H	6.516936	-1.052427	-2.488583
H	5.007677	-0.458995	-0.628975
H	1.624813	-1.760983	-2.960026
H	3.140431	-2.366957	-4.815270
H	3.072772	-4.547083	3.869665
H	4.135915	-4.854435	1.642519
H	3.803041	-3.177638	-0.139312
H	1.364315	-0.835475	2.540925
H	1.691207	-2.521948	4.312945
H	2.027621	3.663016	-3.269189
H	2.826395	4.174896	-0.501309
Cl	-0.534166	-0.329348	-2.622602
H	0.825269	-3.857401	0.858504
C	-4.389096	-1.881456	0.219673

C	-3.362135	-1.704394	-0.742060
C	-3.579983	-1.979462	-2.107004
C	-4.843412	-2.423414	-2.464438
C	-5.878244	-2.597806	-1.505065
C	-5.668499	-2.332797	-0.161767
H	-2.793808	-1.833269	-2.837540
H	-5.053794	-2.644030	-3.507946
H	-6.852150	-2.945395	-1.839852
H	-6.455781	-2.460019	0.576124
N	-2.288013	-1.247875	-0.036072
N	-2.636169	-1.158184	1.255030
N	-3.887933	-1.531163	1.447480
C	-0.139746	-3.656111	0.399778
C	-0.129884	-2.942674	-0.880212
H	-1.015835	-3.095610	-1.496601
H	0.778288	-3.102117	-1.461690
C	-1.244583	-4.034474	1.082117
C	-1.225785	-4.703853	2.419612
H	-1.782873	-4.105083	3.155322
H	-1.722544	-5.684112	2.384190
H	-0.204668	-4.844117	2.791521
H	-2.225994	-3.840917	0.651956

INT20

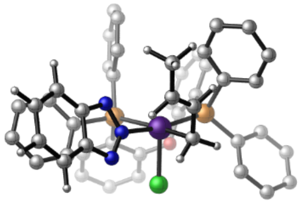

SMD(DCE)-B3LYP-D3/6-31G(d)/LanL2DZ
Thermal correction to Gibbs Free Energy = 0.651305
Sum of electronic and thermal Free Energies = -3267.811572
SMD(DCE)-B3LYP-D3/6-311++G(d,p)/SDD
Single point energy = -3270.1713486
Corrected quasi-harmonic Free Energy = -3269.52791
N_{imag} = 0

Rh	-0.145664	-0.814165	-0.645856
P	-0.726602	1.565905	0.211583
P	2.187662	-0.736078	-0.160463
C	-0.061823	3.044500	-0.687498
C	-0.361217	1.820187	1.991878
C	-2.537751	1.876609	0.070301
C	2.846072	0.688334	0.798590
C	2.397383	-2.133964	1.012020
C	3.476202	-0.986735	-1.445460
C	1.223543	3.041514	-1.256516
O	2.092382	1.987954	-1.012989
C	2.705958	1.957452	0.224425
C	-0.865191	4.172550	-0.934584
C	-0.419712	5.223502	-1.737405
C	0.842754	5.164792	-2.330710
C	1.670371	4.069514	-2.083575
C	3.503726	0.586957	2.033682
C	3.988072	1.727262	2.676953
C	3.832580	2.982311	2.083713
C	3.192829	3.104653	0.850338
C	1.651091	-2.102804	2.204676
C	1.669489	-3.190110	3.077011
C	2.426046	-4.324560	2.764580
C	3.172435	-4.359214	1.584004

C	3.160787	-3.269231	0.708951
C	4.829617	-0.801491	-1.111339
C	5.825962	-1.024599	-2.061014
C	5.483398	-1.433278	-3.353806
C	4.141449	-1.617290	-3.691339
C	3.138981	-1.396301	-2.742896
C	0.235717	2.975668	2.511140
C	0.529900	3.061557	3.874888
C	0.217502	2.003687	4.731316
C	-0.397683	0.854806	4.222799
C	-0.681390	0.760131	2.860441
C	-3.353258	2.194432	1.163676
C	-4.728999	2.369651	0.988007
C	-5.299455	2.233073	-0.278736
C	-4.487569	1.925255	-1.375174
C	-3.115781	1.747408	-1.204130
H	-1.860509	4.223239	-0.507795
H	-1.066296	6.079410	-1.907919
H	3.634357	-0.382709	2.500455
H	4.486295	1.632517	3.637120
H	4.209994	3.872170	2.579444
H	0.481635	3.803110	1.853726
H	1.005513	3.957089	4.265709
H	0.450275	2.073254	5.790465
H	-0.652280	0.031224	4.884588
H	-1.170617	-0.128264	2.467283
H	-2.924966	2.298939	2.155169
H	-5.352552	2.609924	1.845250
H	-6.370571	2.358807	-0.412003
H	-4.924899	1.808116	-2.362846
H	-2.493820	1.487393	-2.055278
H	1.188642	5.969591	-2.972976
H	6.260514	-1.602568	-4.094338
H	6.868394	-0.877767	-1.791595
H	5.106808	-0.483571	-0.110766
H	2.100125	-1.516476	-3.021160
H	3.867669	-1.927543	-4.696059
H	2.435814	-5.175299	3.440329
H	3.764814	-5.236577	1.339356
H	3.738444	-3.311963	-0.208588
H	1.063437	-1.224110	2.455486
H	1.091336	-3.153253	3.996183
H	2.666146	4.000095	-2.509924
H	3.065961	4.073697	0.380287
Cl	-0.017851	0.018184	-2.862635
H	0.556753	-3.929017	0.427896
C	-4.083930	-1.425233	0.184395
C	-4.212695	-1.579285	-1.223753
C	-5.463855	-1.856097	-1.813660
C	-6.551388	-1.972400	-0.964593
C	-6.420587	-1.821544	0.445586
C	-5.199455	-1.549752	1.038520
H	-5.563955	-1.966868	-2.889903
H	-7.534209	-2.182664	-1.378596
H	-7.307790	-1.920662	1.065799
H	-5.099681	-1.425092	2.113173
N	-2.982050	-1.384061	-1.778983
N	-2.193280	-1.122706	-0.749225
N	-2.778217	-1.141650	0.443623
C	-0.277877	-3.645312	-0.208246
C	0.024186	-2.814019	-1.380091
H	-0.725620	-2.849438	-2.171184

H	1.025808	-2.971527	-1.780774
C	-1.515157	-4.046374	0.161200
C	-1.821518	-4.836049	1.393783
H	-2.523808	-4.283787	2.035822
H	-2.314256	-5.788074	1.149487
H	-0.918307	-5.051553	1.975785
H	-2.364964	-3.774578	-0.463673

TS19-21

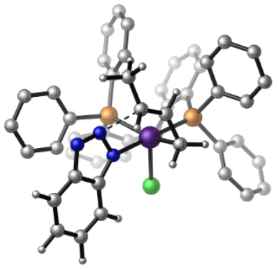

SMD(DCE)-B3LYP-D3/6-31G(d)/LanL2DZ
Thermal correction to Gibbs Free Energy = 0.652672
Sum of electronic and thermal Free Energies = -3267.790701
SMD(DCE)-B3LYP-D3/6-311++G(d,p)/SDD
Single point energy = -3270.1519642
Corrected quasi-harmonic Free Energy = -3269.507748
N_{imag} = 1, ν = -219.0327i cm^{-1}

Rh	-0.246793	-1.038789	-0.065607
P	-0.925323	1.390469	0.122662
P	2.125879	-0.664696	-0.164183
C	-0.784059	2.500426	-1.353307
C	-0.060258	2.295730	1.472641
C	-2.703202	1.585403	0.577107
C	2.827081	1.043077	-0.063349
C	3.013957	-1.520669	1.216955
C	2.959446	-1.358915	-1.656335
C	0.335020	2.391211	-2.195529
O	1.375692	1.549030	-1.839240
C	2.310989	2.008693	-0.939600
C	-1.787033	3.406729	-1.737818
C	-1.693146	4.131469	-2.927710
C	-0.592850	3.959305	-3.769788
C	0.430654	3.086980	-3.398004
C	3.864452	1.432923	0.798472
C	4.325120	2.750171	0.819842
C	3.763949	3.699368	-0.036534
C	2.759076	3.329963	-0.929344
C	2.891824	-1.024904	2.528820
C	3.492084	-1.685931	3.599460
C	4.214692	-2.863102	3.380488
C	4.337286	-3.365922	2.084739
C	3.743806	-2.699216	1.008050
C	4.154000	-0.800391	-2.136346
C	4.822727	-1.382008	-3.215837
C	4.312012	-2.533089	-3.820070
C	3.125768	-3.097200	-3.343726
C	2.448435	-2.511257	-2.273223
C	0.249390	3.661326	1.418557
C	0.890500	4.279899	2.494763
C	1.212593	3.546659	3.639785
C	0.894903	2.186800	3.706603
C	0.270291	1.564900	2.624728
C	-3.114785	1.996916	1.852269
C	-4.473229	2.033688	2.180068

C	-5.435790	1.666240	1.238852
C	-5.033906	1.264128	-0.038814
C	-3.680126	1.218474	-0.365655
H	-2.659908	3.540935	-1.109345
H	-2.486498	4.822595	-3.197589
H	4.318922	0.709993	1.464305
H	5.120472	3.029636	1.504345
H	4.113413	4.727844	-0.020643
H	0.001198	4.240558	0.534492
H	1.139693	5.336099	2.436354
H	1.711069	4.031548	4.474783
H	1.141472	1.608857	4.593472
H	0.036643	0.504623	2.665969
H	-2.382590	2.284353	2.599404
H	-4.774919	2.350853	3.175011
H	-6.491139	1.690111	1.496985
H	-5.772649	0.965930	-0.777580
H	-3.383473	0.891291	-1.357110
H	-0.523157	4.507188	-4.705102
H	4.833191	-2.986400	-4.659163
H	5.743335	-0.934777	-3.581315
H	4.568215	0.088043	-1.670633
H	1.515042	-2.938042	-1.930059
H	2.717183	-3.988722	-3.812048
H	4.679075	-3.381898	4.214638
H	4.897860	-4.279025	1.903604
H	3.854401	-3.105000	0.008394
H	2.331916	-0.113981	2.713414
H	3.392041	-1.283006	4.603718
H	1.309906	2.942110	-4.017608
H	2.325757	4.052909	-1.611433
Cl	-0.413132	-0.965274	-2.548502
H	0.768293	-1.996334	2.275702
C	-4.463460	-2.114719	0.223315
C	-3.365574	-1.940572	-0.665753
C	-3.524512	-2.168318	-2.051211
C	-4.777786	-2.558331	-2.489481
C	-5.876355	-2.725617	-1.599328
C	-5.736074	-2.507496	-0.241886
H	-2.695107	-2.029477	-2.731319
H	-4.932554	-2.742389	-3.549633
H	-6.838802	-3.030743	-2.001466
H	-6.565031	-2.624972	0.450058
N	-2.318957	-1.532829	0.111367
N	-2.781326	-1.460381	1.379217
N	-4.041682	-1.818719	1.485660
C	-0.067872	-2.438126	1.745278
C	0.134061	-3.067878	0.462789
H	-0.652326	-3.729088	0.098170
H	1.135369	-3.418412	0.222803
C	-1.251872	-2.554821	2.496355
C	-1.391684	-1.991064	3.868189
H	-2.436222	-1.756766	4.092533
H	-1.062841	-2.753501	4.590444
H	-0.776429	-1.098681	4.012939
H	-1.935275	-3.360015	2.243523

TS20-22

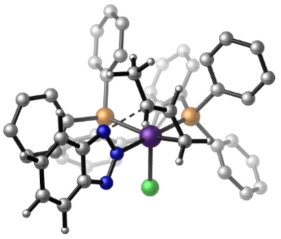

SMD(DCE)-B3LYP-D3/6-31G(d)/LanL2DZ
Thermal correction to Gibbs Free Energy = 0.652631
Sum of electronic and thermal Free Energies = -3267.790853
SMD(DCE)-B3LYP-D3/6-311++G(d,p)/SDD
Single point energy = -3270.1509646
Corrected quasi-harmonic Free Energy = -3269.506658
N_{imag} = 1, ν = -232.1859i cm^{-1}

Rh	-0.173459	-0.950718	-0.497199
P	-0.816990	1.388876	0.217774
P	2.195290	-0.717983	-0.178031
C	-0.409719	2.835435	-0.865633
C	-0.168900	1.851254	1.877812
C	-2.642489	1.577536	0.418888
C	2.904672	0.850708	0.492987
C	2.848978	-1.973488	1.014924
C	3.229591	-1.019366	-1.674482
C	0.821924	2.871714	-1.539779
O	1.769060	1.895055	-1.279010
C	2.558754	2.042777	-0.159539
C	-1.308774	3.885124	-1.120650
C	-1.006716	4.893322	-2.038161
C	0.203943	4.869720	-2.733308
C	1.126425	3.854402	-2.478695
C	3.792519	0.932783	1.576865
C	4.271660	2.166043	2.021390
C	3.881752	3.339899	1.373813
C	3.028533	3.283380	0.272608
C	2.532303	-1.857333	2.381591
C	2.954373	-2.821887	3.295611
C	3.689907	-3.928117	2.858552
C	4.004819	-4.055242	1.505128
C	3.590881	-3.083758	0.588154
C	4.488610	-0.415631	-1.815195
C	5.298044	-0.716929	-2.912706
C	4.864489	-1.632115	-3.874624
C	3.614605	-2.241561	-3.737123
C	2.797790	-1.933012	-2.647940
C	0.171476	3.160425	2.243742
C	0.648644	3.429732	3.528878
C	0.776414	2.399336	4.464013
C	0.425433	1.092869	4.111049
C	-0.036475	0.821293	2.822474
C	-3.260519	1.650290	1.675248
C	-4.653914	1.695511	1.776493
C	-5.445592	1.674682	0.626734
C	-4.836335	1.610917	-0.629726
C	-3.447330	1.554747	-0.733669
H	-2.263362	3.911023	-0.608022
H	-1.723587	5.690334	-2.213298
H	4.114080	0.032652	2.085850
H	4.948378	2.205050	2.869836
H	4.247535	4.303381	1.717514
H	0.076162	3.968349	1.524808
H	0.923715	4.446195	3.797601

H	1.149437	2.613018	5.462062
H	0.520660	0.285508	4.832555
H	-0.295385	-0.195088	2.538906
H	-2.662290	1.666506	2.580379
H	-5.117825	1.747371	2.758165
H	-6.528881	1.702251	0.707023
H	-5.442410	1.583986	-1.530840
H	-2.988858	1.484474	-1.714564
H	0.436782	5.641677	-3.461142
H	5.495188	-1.867117	-4.727988
H	6.267618	-0.236134	-3.012315
H	4.843933	0.290623	-1.071793
H	1.819591	-2.388013	-2.563738
H	3.266487	-2.950233	-4.483846
H	4.015318	-4.683027	3.569065
H	4.577420	-4.909941	1.155198
H	3.849374	-3.200797	-0.458658
H	1.960922	-1.004265	2.733707
H	2.705408	-2.710151	4.347512
H	2.085895	3.815638	-2.984743
H	2.730423	4.186237	-0.248489
Cl	-0.021502	-0.267252	-2.881955
H	0.427131	-2.552263	1.614900
C	-4.207837	-1.782874	0.016907
C	-4.235845	-1.654210	-1.393602
C	-5.438704	-1.787712	-2.113434
C	-6.583116	-2.068442	-1.383186
C	-6.549658	-2.211993	0.031018
C	-5.373649	-2.070609	0.751503
H	-5.462462	-1.668060	-3.192788
H	-7.533596	-2.179327	-1.898251
H	-7.476002	-2.428012	0.556857
H	-5.352325	-2.159459	1.833654
N	-2.967208	-1.359933	-1.815766
N	-2.235323	-1.317190	-0.725553
N	-2.922320	-1.550284	0.410562
C	-0.326863	-2.782100	0.870106
C	0.058649	-3.066395	-0.490744
H	-0.676679	-3.552712	-1.132649
H	1.076404	-3.405059	-0.670359
C	-1.618228	-3.019498	1.381651
C	-1.944111	-2.832268	2.824958
H	-3.009433	-2.636314	2.973002
H	-1.704096	-3.766194	3.355519
H	-1.360188	-2.026657	3.278951
H	-2.264770	-3.697990	0.833417

INT21

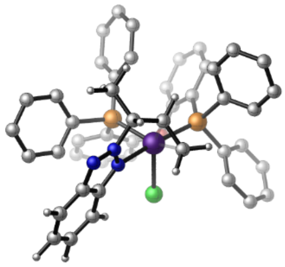

SMD(DCE)-B3LYP-D3/6-31G(d)/LanL2DZ
Thermal correction to Gibbs Free Energy = 0.658467
Sum of electronic and thermal Free Energies = -3267.836321
SMD(DCE)-B3LYP-D3/6-311++G(d,p)/SDD
Single point energy = -3270.1998529
Corrected quasi-harmonic Free Energy = -3269.54989

$N_{imag} = 0$

Rh	-0.200212	-1.043938	-0.005112
P	-0.893167	1.362404	0.076598
P	2.125407	-0.703374	-0.088525
C	-0.775043	2.378886	-1.469891
C	-0.054015	2.427547	1.332183
C	-2.669313	1.573101	0.547993
C	2.831932	1.004472	-0.042955
C	3.050481	-1.535367	1.287795
C	2.920931	-1.437357	-1.580853
C	0.367203	2.250821	-2.278102
O	1.411109	1.441793	-1.861153
C	2.318526	1.940007	-0.953942
C	-1.788545	3.237670	-1.926344
C	-1.680058	3.903461	-3.149615
C	-0.551926	3.719319	-3.951071
C	0.480483	2.890827	-3.509583
C	3.844099	1.430949	0.829619
C	4.282577	2.756313	0.828877
C	3.720980	3.676274	-0.058447
C	2.740970	3.269361	-0.963258
C	2.921150	-1.058702	2.605092
C	3.571420	-1.693037	3.663545
C	4.355046	-2.826246	3.427364
C	4.489315	-3.310296	2.125462
C	3.845774	-2.669164	1.062594
C	4.086099	-0.881086	-2.128088
C	4.721524	-1.495902	-3.209453
C	4.206339	-2.678632	-3.744965
C	3.049617	-3.241637	-3.198556
C	2.404554	-2.621653	-2.127444
C	-0.004938	3.825809	1.225513
C	0.586564	4.590162	2.232853
C	1.127539	3.969034	3.362554
C	1.075982	2.578058	3.480218
C	0.491715	1.814077	2.467887
C	-3.062065	2.031532	1.815237
C	-4.413374	2.065506	2.171583
C	-5.392557	1.652452	1.266122
C	-5.013295	1.212969	-0.005621
C	-3.665017	1.171887	-0.358697
H	-2.679813	3.384778	-1.326897
H	-2.482086	4.559851	-3.475312
H	4.292617	0.732058	1.524718
H	5.058311	3.066077	1.522865
H	4.050037	4.711632	-0.056930
H	-0.423338	4.319686	0.353694
H	0.625269	5.671852	2.134498
H	1.588832	4.566446	4.144418
H	1.496561	2.086341	4.353554
H	0.455277	0.733656	2.553779
H	-2.318968	2.364005	2.532325
H	-4.696926	2.418947	3.159541
H	-6.442367	1.676493	1.545876
H	-5.763434	0.888457	-0.721618
H	-3.386939	0.833109	-1.350396
H	-0.468398	4.223322	-4.909709
H	4.701581	-3.157851	-4.585495
H	5.619395	-1.050261	-3.629632
H	4.502526	0.031372	-1.713101
H	1.492970	-3.045086	-1.724106

H	2.639025	-4.157997	-3.614337
H	4.858096	-3.324461	4.251623
H	5.098237	-4.188589	1.928665
H	3.967160	-3.061488	0.059092
H	2.326156	-0.175110	2.805332
H	3.463829	-1.301620	4.671595
H	1.378309	2.735019	-4.099282
H	2.306847	3.969220	-1.668749
Cl	-0.437790	-1.073268	-2.645139
H	0.605517	-1.816210	2.541885
C	-4.324616	-2.532309	0.358440
C	-3.374315	-1.958320	-0.534681
C	-3.693066	-1.737496	-1.893098
C	-4.964998	-2.089222	-2.296664
C	-5.922799	-2.651945	-1.400089
C	-5.623216	-2.884003	-0.074485
H	-2.953557	-1.317132	-2.563088
H	-5.251838	-1.935376	-3.333276
H	-6.908478	-2.905597	-1.779918
H	-6.340585	-3.315014	0.616420
N	-2.252022	-1.707977	0.191804
N	-2.552250	-2.152251	1.417835
N	-3.758631	-2.647656	1.584749
C	-0.169063	-2.094595	1.834180
C	0.161042	-3.007134	0.774111
H	-0.586833	-3.711759	0.409263
H	1.178193	-3.383899	0.707519
C	-1.539176	-2.124031	2.487948
C	-1.814566	-0.956336	3.434314
H	-2.812854	-1.044890	3.874139
H	-1.074414	-0.964293	4.241303
H	-1.748858	-0.006221	2.902927
H	-1.680659	-3.067455	3.028148

INT22

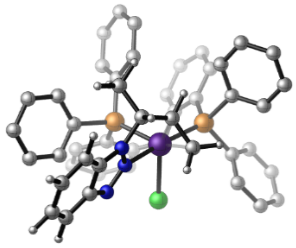

SMD(DCE)-B3LYP-D3/6-31G(d)/LanL2DZ
Thermal correction to Gibbs Free Energy = 0.657448
Sum of electronic and thermal Free Energies = -3267.837186
SMD(DCE)-B3LYP-D3/6-311++G(d,p)/SDD
Single point energy = -3270.1998114
Corrected quasi-harmonic Free Energy = -3269.550769
N_{imag} = 0

Rh	-0.252326	-0.905880	-0.378701
P	-0.657352	1.502820	0.107866
P	2.080948	-0.905113	-0.076890
C	-0.193215	2.749078	-1.182373
C	0.108817	2.186979	1.642807
C	-2.448464	1.866128	0.387568
C	2.976133	0.645170	0.385068
C	2.651660	-2.083234	1.236203
C	3.022365	-1.486850	-1.552584
C	1.034072	2.605611	-1.850833
O	1.901150	1.593353	-1.474963

C	2.726386	1.787535	-0.390640
C	-1.022473	3.808586	-1.585254
C	-0.656949	4.658611	-2.631650
C	0.549612	4.462261	-3.306005
C	1.402359	3.431251	-2.909779
C	3.895885	0.758256	1.438199
C	4.500671	1.981154	1.734949
C	4.200006	3.111282	0.972102
C	3.316229	3.017992	-0.102447
C	2.368291	-1.808404	2.586386
C	2.747181	-2.697096	3.592294
C	3.405562	-3.886522	3.266105
C	3.686981	-4.172845	1.929632
C	3.316769	-3.277516	0.921337
C	4.332649	-1.048476	-1.796169
C	5.061109	-1.563079	-2.871062
C	4.492988	-2.528815	-3.705251
C	3.190603	-2.974216	-3.463671
C	2.455035	-2.451790	-2.398526
C	0.412527	3.547230	1.800762
C	0.949393	4.015496	3.001755
C	1.178576	3.134336	4.062353
C	0.869902	1.779454	3.916767
C	0.343249	1.310590	2.711779
C	-2.966246	2.133210	1.664732
C	-4.342073	2.287228	1.856480
C	-5.219232	2.188174	0.774958
C	-4.711819	1.940208	-0.503893
C	-3.340053	1.776398	-0.696105
H	-1.970831	3.968905	-1.084991
H	-1.320606	5.468342	-2.921565
H	4.141648	-0.107930	2.040504
H	5.201422	2.047431	2.561998
H	4.660234	4.067706	1.204078
H	0.238803	4.241742	0.984587
H	1.191093	5.069848	3.107267
H	1.598153	3.501830	4.995018
H	1.047115	1.085754	4.734526
H	0.112145	0.257469	2.592394
H	-2.301009	2.219963	2.517318
H	-4.723648	2.489549	2.853941
H	-6.289081	2.306268	0.924944
H	-5.384292	1.862595	-1.353770
H	-2.961385	1.574098	-1.691549
H	0.832190	5.110317	-4.130745
H	5.060836	-2.930496	-4.540455
H	6.072877	-1.210074	-3.052601
H	4.790745	-0.306332	-1.150084
H	1.436629	-2.779324	-2.230672
H	2.739375	-3.720607	-4.112093
H	3.698079	-4.581618	4.048413
H	4.200636	-5.093085	1.664537
H	3.549755	-3.518626	-0.110006
H	1.866744	-0.885783	2.856045
H	2.526626	-2.459408	4.629520
H	2.353010	3.256347	-3.403683
H	3.084109	3.884091	-0.712494
Cl	-0.127187	-0.447053	-2.959076
H	0.013382	-2.275020	2.036921
C	-4.229096	-2.110378	0.131791
C	-4.385082	-1.572316	-1.164317
C	-5.635612	-1.598547	-1.808083

C	-6.686703	-2.178823	-1.117146
C	-6.512979	-2.725647	0.182447
C	-5.288695	-2.705508	0.833592
H	-5.757968	-1.177758	-2.800960
H	-7.671018	-2.219506	-1.574097
H	-7.369289	-3.170919	0.680902
H	-5.156654	-3.117457	1.828160
N	-3.180127	-1.062634	-1.579538
N	-2.332179	-1.250959	-0.619106
N	-2.919484	-1.887846	0.426467
C	-0.655528	-2.292737	1.181678
C	-0.258049	-3.016900	0.006181
H	-1.009742	-3.523714	-0.599799
H	0.709730	-3.511192	-0.003574
C	-2.117725	-2.251987	1.601754
C	-2.413159	-1.299875	2.760836
H	-3.479237	-1.312929	3.011355
H	-1.845441	-1.610715	3.644480
H	-2.132197	-0.278134	2.499104
H	-2.447881	-3.263389	1.874911

INT13*

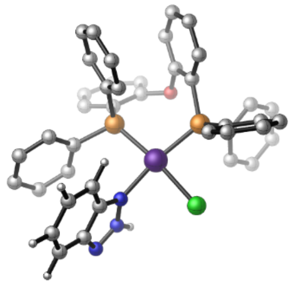

SMD(DCE)-B3LYP-D3/6-31G(d)/LanL2DZ
Thermal correction to Gibbs Free Energy = 0.568039
Sum of electronic and thermal Free Energies = -3111.887036
SMD(DCE)-B3LYP-D3/6-311++G(d,p)/SDD
Single point energy = -3114.1171517
Corrected quasi-harmonic Free Energy = -3113.555388
N_{imag} = 0

Rh	0.220484	-0.939521	0.603813
P	0.901382	1.179628	0.028709
P	-2.006800	-0.752132	0.066392
C	0.341078	2.677241	0.996568
C	0.638660	1.697598	-1.725122
C	2.738922	1.330782	0.203058
C	-2.569762	0.694141	-0.924555
C	-2.550004	-2.156158	-1.005448
C	-3.205269	-0.731227	1.474561
C	-1.005193	2.855669	1.363419
O	-1.964245	1.935251	0.979687
C	-2.406793	1.952863	-0.333819
C	1.243822	3.673750	1.412009
C	0.833032	4.759496	2.187891
C	-0.501912	4.878847	2.576442
C	-1.425676	3.923050	2.155142
C	-3.062903	0.635852	-2.233584
C	-3.362756	1.809386	-2.930632
C	-3.177367	3.052623	-2.321249
C	-2.701299	3.132992	-1.010614
C	-1.641950	-2.659262	-1.951304
C	-2.020407	-3.673174	-2.831729
C	-3.310471	-4.208232	-2.770857

C	-4.217420	-3.721016	-1.827186
C	-3.841995	-2.698282	-0.951663
C	-4.501733	-0.212943	1.318641
C	-5.387481	-0.182735	2.397350
C	-4.987056	-0.663087	3.647232
C	-3.697587	-1.174463	3.811370
C	-2.810157	-1.208049	2.732898
C	0.666441	3.041756	-2.126033
C	0.558898	3.376427	-3.476916
C	0.426787	2.373613	-4.442050
C	0.398243	1.032958	-4.050905
C	0.500784	0.698504	-2.699124
C	3.591322	1.449168	-0.901098
C	4.979848	1.437895	-0.727961
C	5.529603	1.311660	0.547229
C	4.684606	1.206868	1.658553
C	3.302736	1.214421	1.486683
H	2.288157	3.602870	1.132977
H	1.561728	5.505725	2.491144
H	-3.197054	-0.326036	-2.717026
H	-3.732609	1.751099	-3.950146
H	-3.403063	3.966128	-2.864213
H	0.770068	3.829150	-1.385686
H	0.576454	4.421427	-3.774987
H	0.342081	2.637277	-5.492960
H	0.289369	0.247373	-4.793913
H	0.469833	-0.341619	-2.389675
H	3.182760	1.535017	-1.902133
H	5.627548	1.519147	-1.596725
H	6.607969	1.293289	0.678843
H	5.102728	1.108170	2.656486
H	2.654567	1.120662	2.353590
H	-0.827021	5.713159	3.191343
H	-5.675397	-0.635304	4.487957
H	-6.387734	0.220290	2.261480
H	-4.820961	0.175334	0.355840
H	-1.811780	-1.610582	2.862559
H	-3.377187	-1.546125	4.781287
H	-3.604469	-5.003627	-3.450616
H	-5.219984	-4.136571	-1.768099
H	-4.560650	-2.332261	-0.225892
H	-0.631191	-2.264682	-1.984836
H	-1.305112	-4.051424	-3.557421
H	-2.476408	3.989937	2.419428
H	-2.551937	4.091101	-0.523771
Cl	-0.272807	-3.248710	1.392682
H	2.162602	-1.584293	3.021643
C	3.220589	-1.904975	0.148509
C	4.310449	-2.335650	0.958532
C	5.520013	-2.774951	0.371241
C	5.581718	-2.766806	-1.006031
C	4.482049	-2.338749	-1.812466
C	3.296394	-1.902501	-1.260809
H	6.352843	-3.099805	0.986379
H	6.490394	-3.095945	-1.501899
H	4.593841	-2.355312	-2.892764
H	2.459947	-1.564499	-1.862375
N	3.951902	-2.227725	2.262578
N	2.723568	-1.755405	2.193134
N	2.223963	-1.530988	0.985136

INT14*

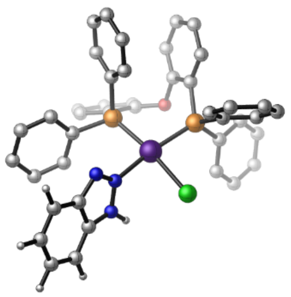

SMD(DCE)-B3LYP-D3/6-31G(d)/LanL2DZ
Thermal correction to Gibbs Free Energy = 0.564895
Sum of electronic and thermal Free Energies = -3111.890850
SMD(DCE)-B3LYP-D3/6-311++G(d,p)/SDD
Single point energy = -3114.1174255
Corrected quasi-harmonic Free Energy = -3113.557858
N_{imag} = 0

Rh	0.325957	-0.941061	0.169330
P	0.664662	1.327408	0.001617
P	-1.969371	-0.968795	0.039751
C	0.105068	2.513445	1.335005
C	0.066923	2.138647	-1.545929
C	2.478092	1.702119	-0.025141
C	-2.844750	0.544768	-0.535529
C	-2.532049	-2.207212	-1.209696
C	-2.902047	-1.383976	1.580910
C	-1.174763	2.432852	1.912634
O	-2.078697	1.476845	1.484668
C	-2.729994	1.678044	0.277800
C	0.951454	3.528079	1.819262
C	0.558409	4.382301	2.851268
C	-0.700817	4.242143	3.436029
C	-1.572640	3.264201	2.958504
C	-3.537408	0.665858	-1.746391
C	-4.079663	1.894311	-2.133111
C	-3.941664	3.011788	-1.306524
C	-3.267668	2.909145	-0.087432
C	-1.757106	-2.378815	-2.368752
C	-2.168820	-3.253009	-3.375213
C	-3.355465	-3.978192	-3.231470
C	-4.127093	-3.820916	-2.078260
C	-3.720656	-2.938147	-1.073826
C	-4.259008	-1.048865	1.723194
C	-4.943838	-1.350984	2.901549
C	-4.280178	-1.985908	3.955047
C	-2.929683	-2.317702	3.822694
C	-2.242494	-2.018153	2.643713
C	-0.132431	3.524177	-1.645009
C	-0.505231	4.096425	-2.862413
C	-0.679000	3.292682	-3.993088
C	-0.480285	1.912798	-3.902467
C	-0.110852	1.339119	-2.684310
C	3.124896	2.224960	-1.150541
C	4.512328	2.406561	-1.148315
C	5.265148	2.068397	-0.023954
C	4.625033	1.549208	1.107410
C	3.244090	1.366635	1.105742
H	1.937419	3.653172	1.388795
H	1.242272	5.151636	3.198257
H	-3.639446	-0.195271	-2.398322
H	-4.603565	1.977649	-3.080834
H	-4.357177	3.969216	-1.607907

H	0.000768	4.157830	-0.773380
H	-0.663022	5.169823	-2.926563
H	-0.972149	3.740271	-4.939057
H	-0.618811	1.280981	-4.775725
H	0.038221	0.265611	-2.607556
H	2.556954	2.484433	-2.037581
H	5.000385	2.809918	-2.031695
H	6.343371	2.203443	-0.026383
H	5.203149	1.279614	1.987247
H	2.756759	0.952423	1.984252
H	-1.009552	4.893651	4.248460
H	-4.812306	-2.216591	4.874305
H	-5.993491	-1.085429	2.997025
H	-4.782682	-0.543193	0.917120
H	-1.195452	-2.281773	2.541854
H	-2.406128	-2.808479	4.639104
H	-3.673462	-4.664773	-4.011698
H	-5.047392	-4.386022	-1.955374
H	-4.333083	-2.828110	-0.185335
H	-0.822039	-1.836499	-2.473223
H	-1.558100	-3.374903	-4.265970
H	-2.566994	3.134424	3.374030
H	-3.151769	3.768185	0.565249
Cl	0.221998	-3.407163	0.518235
H	2.547547	-2.121281	1.951973
C	4.431170	-1.662463	-0.566467
C	4.357337	-2.122575	0.767685
C	5.462132	-2.671800	1.435983
C	6.642398	-2.732334	0.710429
C	6.732074	-2.267445	-0.628771
C	5.638114	-1.728943	-1.285355
H	5.394757	-3.024171	2.459700
H	7.529391	-3.147789	1.180037
H	7.685712	-2.337433	-1.143564
H	5.696410	-1.366273	-2.306408
N	3.068531	-1.867931	1.120322
N	2.406871	-1.313918	0.084394
N	3.198497	-1.179016	-0.934701

TS13-15*

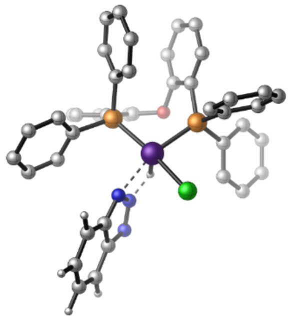

SMD(DCE)-B3LYP-D3/6-31G(d)/LanL2DZ
Thermal correction to Gibbs Free Energy = 0.559929
Sum of electronic and thermal Free Energies = -3111.848121
SMD(DCE)-B3LYP-D3/6-311++G(d,p)/SDD
Single point energy = -3114.0745069
Corrected quasi-harmonic Free Energy = -3113.520462
N_{imag} = 1, ν = -1225.2012i cm^{-1}

Rh	-0.441237	-0.787006	0.479527
P	-0.680448	1.476899	0.077004
P	1.806409	-0.975829	0.084814
C	-0.673658	2.117024	-1.655032

C	0.513110	2.526391	1.000706
C	-2.320664	2.001613	0.749440
C	2.747485	0.532053	-0.388190
C	2.786595	-1.640657	1.493579
C	2.163538	-2.099819	-1.338794
C	0.243582	1.611412	-2.595919
O	1.228133	0.718293	-2.202301
C	2.315141	1.245246	-1.515069
C	-1.598099	3.074663	-2.106587
C	-1.643450	3.462161	-3.446710
C	-0.765530	2.892708	-4.370512
C	0.187777	1.968887	-3.940908
C	3.845456	1.027615	0.327649
C	4.488355	2.199841	-0.075022
C	4.047093	2.883740	-1.209578
C	2.956526	2.407157	-1.938407
C	2.334896	-1.407165	2.801210
C	3.088483	-1.831496	3.896472
C	4.295927	-2.505732	3.696209
C	4.745370	-2.755292	2.396940
C	3.996427	-2.324682	1.299761
C	3.345997	-1.949390	-2.083756
C	3.615734	-2.793012	-3.162616
C	2.705811	-3.792247	-3.516559
C	1.527340	-3.945764	-2.782729
C	1.254808	-3.106291	-1.700117
C	0.937137	3.776215	0.530509
C	1.811177	4.551934	1.295421
C	2.260942	4.092285	2.535614
C	1.836437	2.849119	3.012656
C	0.970329	2.069206	2.246727
C	-2.422007	2.581406	2.022633
C	-3.674871	2.864347	2.574612
C	-4.839344	2.579358	1.860810
C	-4.747120	2.009928	0.586758
C	-3.500206	1.718068	0.036699
H	-2.300101	3.515626	-1.408574
H	-2.372205	4.201127	-3.766168
H	4.194030	0.503032	1.210354
H	5.329691	2.576239	0.499034
H	4.546747	3.793742	-1.529172
H	0.597910	4.141484	-0.433696
H	2.143899	5.514847	0.917613
H	2.943248	4.698112	3.125584
H	2.184228	2.481468	3.974101
H	0.644092	1.102739	2.617439
H	-1.530989	2.815645	2.594862
H	-3.733983	3.311809	3.563107
H	-5.812556	2.798788	2.291502
H	-5.646776	1.782230	0.021917
H	-3.448139	1.266466	-0.947723
H	-0.808239	3.176335	-5.418026
H	2.912792	-4.443877	-4.361323
H	4.534298	-2.662675	-3.728624
H	4.057258	-1.169781	-1.829950
H	0.338495	-3.236882	-1.136082
H	0.811954	-4.717901	-3.052891
H	4.880525	-2.841525	4.548541
H	5.678601	-3.287740	2.234257
H	4.358318	-2.525057	0.296530
H	1.384145	-0.911578	2.962516
H	2.726714	-1.645057	4.904030

H	0.905632	1.530290	-4.626668
H	2.597691	2.933621	-2.816209
Cl	-0.447836	-3.060799	1.412314
H	-0.987598	-1.101842	-1.104340
C	-3.808053	-1.639950	0.321319
C	-4.258411	-2.222891	-0.897512
C	-5.519666	-2.854412	-0.969357
C	-6.279813	-2.883665	0.185145
C	-5.821572	-2.297966	1.401551
C	-4.594084	-1.668937	1.491750
H	-5.870125	-3.294732	-1.898059
H	-7.255010	-3.362732	0.172383
H	-6.462744	-2.346950	2.277504
H	-4.245844	-1.209646	2.411738
N	-3.304244	-2.026654	-1.857441
N	-2.362507	-1.330296	-1.251816
N	-2.600547	-1.091223	0.053945

TS14-16*

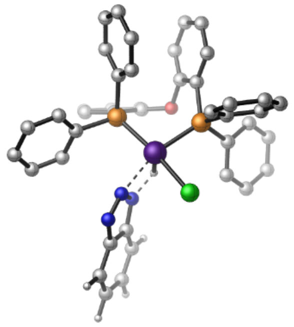

SMD(DCE)-B3LYP-D3/6-31G(d)/LanL2DZ
Thermal correction to Gibbs Free Energy = 0.560356
Sum of electronic and thermal Free Energies = -3111.849481
SMD(DCE)-B3LYP-D3/6-311++G(d,p)/SDD
Single point energy = -3114.0760336
Corrected quasi-harmonic Free Energy = -3113.521716
N_{imag} = 1, ν = -1232.5437i cm^{-1}

Rh	0.387578	-0.546147	-0.872555
P	0.510761	1.625209	-0.056723
P	-1.700350	-1.112167	-0.108589
C	0.776232	1.905985	1.751555
C	-0.907032	2.705340	-0.508960
C	1.943376	2.477608	-0.854976
C	-2.693331	0.167248	0.757052
C	-2.845682	-1.684699	-1.429764
C	-1.651397	-2.468867	1.144906
C	0.064435	1.154014	2.705270
O	-0.905399	0.249873	2.303282
C	-2.131210	0.758011	1.896342
C	1.706372	2.841979	2.236035
C	1.953891	2.979101	3.602865
C	1.277312	2.175158	4.521300
C	0.322459	1.263582	4.069773
C	-3.944619	0.621386	0.321674
C	-4.605444	1.644952	1.004008
C	-4.022424	2.218776	2.135871
C	-2.780431	1.774130	2.592662
C	-2.711529	-1.143053	-2.717417
C	-3.603085	-1.496634	-3.730651
C	-4.631936	-2.406377	-3.471979
C	-4.764450	-2.959872	-2.196171
C	-3.878444	-2.600178	-1.178045

C	-2.694834	-2.607435	2.076673
C	-2.660959	-3.625462	3.031166
C	-1.581881	-4.511907	3.074167
C	-0.539164	-4.376658	2.154892
C	-0.570720	-3.362029	1.195550
C	-1.228518	3.857041	0.223044
C	-2.263118	4.691316	-0.204163
C	-2.978331	4.387856	-1.365923
C	-2.658799	3.243936	-2.101948
C	-1.629542	2.405247	-1.672811
C	1.746684	3.360974	-1.926590
C	2.841201	3.935861	-2.579234
C	4.141253	3.640495	-2.167663
C	4.345386	2.762519	-1.098497
C	3.257602	2.180393	-0.450845
H	2.253108	3.466856	1.539889
H	2.682052	3.708815	3.944240
H	-4.397792	0.184720	-0.561736
H	-5.569214	1.995715	0.647261
H	-4.531605	3.017454	2.667717
H	-0.679517	4.101266	1.127301
H	-2.511184	5.577619	0.373498
H	-3.784122	5.038857	-1.694030
H	-3.213286	2.998507	-3.003533
H	-1.383708	1.514269	-2.241945
H	0.744639	3.608804	-2.259495
H	2.670894	4.617961	-3.407822
H	4.990831	4.089472	-2.674968
H	5.353420	2.523482	-0.770905
H	3.432923	1.497064	0.372702
H	1.477845	2.264766	5.584967
H	-1.552887	-5.300338	3.821677
H	-3.475205	-3.719409	3.744799
H	-3.533309	-1.917946	2.066361
H	0.241113	-3.270205	0.484167
H	0.305977	-5.059392	2.182020
H	-5.322308	-2.687674	-4.262632
H	-5.556954	-3.674319	-1.990408
H	-3.994376	-3.039837	-0.192865
H	-1.899454	-0.457549	-2.935730
H	-3.486486	-1.069815	-4.723093
H	-0.241371	0.641641	4.757725
H	-2.315447	2.215471	3.467796
Cl	0.419694	-2.693103	-2.091641
H	1.294370	-1.054777	0.455728
C	4.455979	-1.689359	-0.942744
C	3.877796	-1.807869	0.349319
C	4.525685	-2.504151	1.386827
C	5.752388	-3.072795	1.085294
C	6.340317	-2.951653	-0.203969
C	5.711580	-2.261216	-1.226880
H	4.078684	-2.590367	2.372591
H	6.285775	-3.626470	1.853321
H	7.306137	-3.415571	-0.384334
H	6.157494	-2.164764	-2.212412
N	2.710324	-1.103038	0.295070
N	2.589487	-0.655083	-0.982635
N	3.601013	-0.968408	-1.740102

INT15*

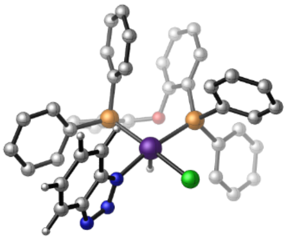

SMD(DCE)-B3LYP-D3/6-31G(d)/LanL2DZ
Thermal correction to Gibbs Free Energy = 0.565304
Sum of electronic and thermal Free Energies = -3111.878798
SMD(DCE)-B3LYP-D3/6-311++G(d,p)/SDD
Single point energy = -3114.1143309
Corrected quasi-harmonic Free Energy = -3113.555943
N_{imag} = 0

Rh	0.305186	-0.912272	0.547712
P	1.010007	1.238987	-0.098063
P	-2.023976	-0.702294	0.098598
C	0.522097	2.724379	0.884296
C	0.475818	1.543130	-1.824349
C	2.843151	1.352127	-0.153261
C	-2.656441	0.813083	-0.721868
C	-2.607353	-2.059609	-0.991990
C	-3.074706	-0.794358	1.607992
C	-0.769747	2.864353	1.423330
O	-1.743993	1.919570	1.147511
C	-2.413447	2.025091	-0.063428
C	1.444334	3.752939	1.148752
C	1.104947	4.840310	1.954408
C	-0.164880	4.919283	2.527812
C	-1.107960	3.928118	2.256331
C	-3.360734	0.843753	-1.932621
C	-3.796350	2.058177	-2.467567
C	-3.543960	3.252236	-1.788973
C	-2.850993	3.243155	-0.576985
C	-2.003159	-2.218112	-2.250092
C	-2.396756	-3.253855	-3.097468
C	-3.391327	-4.149366	-2.693303
C	-3.992573	-3.998805	-1.442024
C	-3.604768	-2.959342	-0.593032
C	-4.405590	-0.345588	1.557452
C	-5.213524	-0.405511	2.693438
C	-4.701944	-0.911063	3.891932
C	-3.381520	-1.361922	3.946756
C	-2.569509	-1.307245	2.811394
C	0.072174	2.803452	-2.282381
C	-0.318675	2.969738	-3.612904
C	-0.295820	1.887131	-4.495243
C	0.122270	0.630445	-4.047539
C	0.502557	0.458642	-2.716924
C	3.544457	1.370425	-1.366559
C	4.942208	1.382162	-1.367967
C	5.646544	1.377282	-0.164038
C	4.950332	1.371589	1.049477
C	3.558251	1.358898	1.057012
H	2.442149	3.703945	0.730269
H	1.840469	5.617137	2.139916
H	-3.566418	-0.079172	-2.463173
H	-4.334093	2.067780	-3.410827
H	-3.886184	4.197603	-2.200097
H	0.047044	3.649173	-1.603211
H	-0.646311	3.946796	-3.956428

H	-0.603617	2.020849	-5.528516
H	0.145761	-0.215271	-4.729000
H	0.827187	-0.519938	-2.373045
H	3.014075	1.369923	-2.312395
H	5.475515	1.390157	-2.314507
H	6.733065	1.375631	-0.167937
H	5.490546	1.361915	1.991546
H	3.028826	1.338138	2.003113
H	-0.428025	5.753585	3.171247
H	-5.329725	-0.952059	4.778023
H	-6.240375	-0.053914	2.641678
H	-4.813264	0.054026	0.633809
H	-1.550386	-1.671019	2.863086
H	-2.976553	-1.757320	4.874311
H	-3.694221	-4.960508	-3.349781
H	-4.764053	-4.692717	-1.119551
H	-4.075649	-2.860268	0.378932
H	-1.236014	-1.523764	-2.576495
H	-1.924626	-3.362305	-4.070192
H	-2.112655	3.970024	2.664191
H	-2.647516	4.164537	-0.042366
Cl	-0.186190	-3.265377	1.040903
H	0.069358	-0.334667	1.927688
C	3.235576	-1.916064	0.319296
C	4.336297	-2.131106	1.181205
C	5.544574	-2.658378	0.683355
C	5.604663	-2.955900	-0.669074
C	4.491326	-2.743930	-1.527114
C	3.297373	-2.224920	-1.052233
H	6.394365	-2.820527	1.340706
H	6.520198	-3.363592	-1.089643
H	4.583935	-2.995883	-2.580382
H	2.446106	-2.063160	-1.708292
N	3.968714	-1.730069	2.440290
N	2.724260	-1.292976	2.362178
N	2.258618	-1.383720	1.105393

INT16*

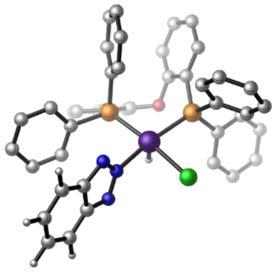

SMD(DCE)-B3LYP-D3/6-31G(d)/LanL2DZ
Thermal correction to Gibbs Free Energy = 0.564393
Sum of electronic and thermal Free Energies = -3111.881460
SMD(DCE)-B3LYP-D3/6-311++G(d,p)/SDD
Single point energy = -3114.1148461
Corrected quasi-harmonic Free Energy = -3113.55698
N_{imag} = 0

Rh	0.294301	-0.954714	0.256913
P	0.846901	1.309454	-0.082807
P	-2.072267	-0.820726	0.092322
C	0.355053	2.586139	1.162800
C	0.192828	1.852588	-1.703281
C	2.664395	1.564975	-0.193979
C	-2.869592	0.742555	-0.444395
C	-2.581362	-2.026621	-1.194255

C	-3.027820	-1.236515	1.605088
C	-0.888914	2.561936	1.818625
O	-1.836404	1.612762	1.478116
C	-2.626687	1.858578	0.364944
C	1.247716	3.612909	1.523235
C	0.930626	4.534290	2.521751
C	-0.287488	4.446012	3.196974
C	-1.202807	3.456918	2.839055
C	-3.695426	0.897323	-1.565344
C	-4.245536	2.145028	-1.868389
C	-3.984085	3.243780	-1.046359
C	-3.173005	3.107584	0.082164
C	-2.065383	-1.862909	-2.491329
C	-2.373735	-2.781726	-3.494633
C	-3.192329	-3.878792	-3.210870
C	-3.703661	-4.048308	-1.922143
C	-3.401092	-3.128136	-0.915693
C	-4.421177	-1.050753	1.607964
C	-5.169145	-1.352556	2.746137
C	-4.533364	-1.833507	3.894549
C	-3.148463	-2.013133	3.898609
C	-2.395872	-1.718526	2.759243
C	-0.411495	3.100002	-1.901822
C	-0.904123	3.443293	-3.162889
C	-0.784843	2.552431	-4.231831
C	-0.161439	1.314866	-4.043915
C	0.323249	0.962886	-2.784255
C	3.289591	1.983494	-1.376027
C	4.676862	2.149843	-1.413441
C	5.446342	1.903550	-0.275474
C	4.824715	1.495807	0.908924
C	3.442412	1.327733	0.951697
H	2.207725	3.691927	1.028081
H	1.643858	5.312624	2.775649
H	-3.904796	0.047336	-2.205273
H	-4.877400	2.255867	-2.744465
H	-4.412588	4.214267	-1.279908
H	-0.510581	3.796595	-1.076285
H	-1.386874	4.405929	-3.305576
H	-1.172829	2.822321	-5.210114
H	-0.056677	0.621281	-4.873490
H	0.816450	0.003723	-2.645591
H	2.707539	2.179332	-2.269800
H	5.151949	2.471176	-2.336281
H	6.525380	2.026192	-0.309138
H	5.416404	1.296478	1.797665
H	2.970437	1.001228	1.871880
H	-0.532119	5.147557	3.989028
H	-5.115616	-2.064537	4.782602
H	-6.246032	-1.207829	2.736265
H	-4.923165	-0.668058	0.723896
H	-1.324051	-1.874261	2.767027
H	-2.648119	-2.384782	4.788670
H	-3.428328	-4.598371	-3.990090
H	-4.336693	-4.901651	-1.694550
H	-3.795467	-3.278377	0.083451
H	-1.433385	-1.010964	-2.723252
H	-1.972247	-2.641355	-4.494577
H	-2.168639	3.370696	3.326487
H	-2.962120	3.954883	0.725374
Cl	-0.083880	-3.341340	0.701520
H	0.232124	-0.620561	1.734518

C	4.216906	-1.919623	-0.426053
C	4.250994	-1.993516	0.993731
C	5.441239	-2.319244	1.677796
C	6.566488	-2.563774	0.909279
C	6.531055	-2.492792	-0.512593
C	5.370104	-2.176665	-1.197087
H	5.468159	-2.369457	2.762887
H	7.504954	-2.814429	1.397229
H	7.443732	-2.691899	-1.068606
H	5.344545	-2.116440	-2.281650
N	3.010882	-1.670302	1.457871
N	2.305349	-1.421296	0.364201
N	2.954941	-1.555411	-0.787175

INT13-p

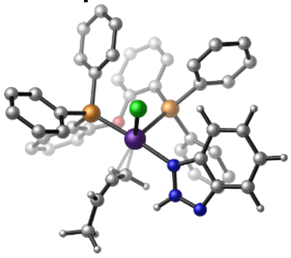

SMD(DCE)-B3LYP-D3/6-31G(d)/LanL2DZ
Thermal correction to Gibbs Free Energy = 0.650220
Sum of electronic and thermal Free Energies = -3267.798806
SMD(DCE)-B3LYP-D3/6-311++G(d,p)/SDD
Single point energy = -3270.1596291
Corrected quasi-harmonic Free Energy = -3269.517118
N_{imag} = 0

Rh	-0.105463	-1.105647	-0.069456
P	2.097777	-0.436547	-0.363070
P	-1.079526	1.269396	0.290690
C	3.064407	-0.220394	1.197690
C	2.366753	1.163267	-1.247407
C	3.138577	-1.575169	-1.373527
C	0.014418	2.713607	0.719508
C	-2.244357	2.064719	-0.900988
C	-2.114802	1.044713	1.812136
C	2.571179	0.652141	2.186340
O	1.328390	1.229052	1.989217
C	1.159306	2.514104	1.510445
C	4.240972	-0.927928	1.483464
C	4.909619	-0.751608	2.697708
C	4.405744	0.128582	3.656205
C	3.222318	0.825828	3.405310
C	-0.208281	4.011529	0.232333
C	0.663813	5.060163	0.531093
C	1.782501	4.830042	1.333148
C	2.033283	3.551660	1.830594
C	-1.972163	1.907767	-2.269028
C	-2.791572	2.502290	-3.230942
C	-3.899576	3.256448	-2.836928
C	-4.177654	3.422222	-1.476725
C	-3.352308	2.837360	-0.514311
C	-3.397323	0.479298	1.690850
C	-4.144635	0.145731	2.820715
C	-3.627838	0.374117	4.098981
C	-2.362524	0.949245	4.232312
C	-1.611370	1.281296	3.101233
C	3.333614	2.095416	-0.843477

C	3.522437	3.274720	-1.568306
C	2.755483	3.532366	-2.705997
C	1.805767	2.596910	-3.125371
C	1.614732	1.418089	-2.404469
C	4.417455	-1.173017	-1.797940
C	5.212730	-2.033666	-2.554630
C	4.735826	-3.300267	-2.906858
C	3.459621	-3.697903	-2.504063
C	2.662595	-2.839091	-1.742920
H	4.641479	-1.625074	0.756550
H	5.820709	-1.309552	2.893040
H	-1.068146	4.208529	-0.397189
H	0.465851	6.052455	0.136318
H	2.465107	5.640808	1.571891
H	3.935985	1.916770	0.040445
H	4.264602	3.994364	-1.233638
H	2.898524	4.454643	-3.262756
H	1.207231	2.784339	-4.012980
H	0.890850	0.683523	-2.739788
H	4.796053	-0.189395	-1.536532
H	6.200897	-1.712701	-2.873167
H	5.354096	-3.968574	-3.500456
H	3.075837	-4.674198	-2.787986
H	1.660898	-3.131556	-1.461413
H	4.919342	0.263145	4.603705
H	-4.207459	0.112305	4.980152
H	-5.131757	-0.292056	2.697113
H	-3.824924	0.307799	0.710293
H	-0.628817	1.714933	3.237839
H	-1.952112	1.142533	5.220179
H	-4.544304	3.711694	-3.584091
H	-5.035983	4.010687	-1.163170
H	-3.574308	2.982781	0.538194
H	-1.140273	1.287026	-2.581001
H	-2.570300	2.363445	-4.285849
H	2.787151	1.489070	4.145925
H	2.905638	3.354145	2.443548
Cl	-0.352083	-1.460414	-2.654698
C	-4.260854	-2.670586	-0.021397
C	-3.282907	-1.834600	-0.635996
C	-3.616559	-1.007835	-1.730250
C	-4.929879	-1.031453	-2.149005
C	-5.914619	-1.857206	-1.525525
C	-5.602274	-2.686238	-0.470007
H	-2.863992	-0.405645	-2.213959
H	-5.227545	-0.404373	-2.984657
H	-6.932606	-1.830520	-1.903577
H	-6.340231	-3.322999	0.006980
N	-2.120406	-2.002482	0.049150
N	-2.439032	-2.903959	0.975416
N	-3.675146	-3.348588	0.995833
H	-1.719015	-3.303702	1.571522
C	0.500054	-2.726904	1.063175
C	0.192886	-1.657261	1.946378
H	0.991126	-1.152917	2.481589
H	-0.750867	-1.626390	2.496229
C	0.806971	-4.020477	1.015212
H	0.928830	-4.515807	0.052165
C	1.012683	-4.888393	2.234126
H	0.326090	-5.746822	2.229241
H	2.031625	-5.300390	2.257988
H	0.851669	-4.322953	3.158573

INT14-p

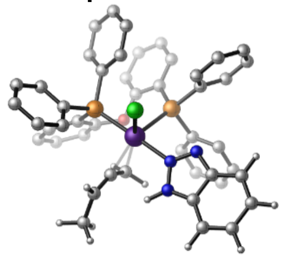

SMD(DCE)-B3LYP-D3/6-31G(d)/LanL2DZ
Thermal correction to Gibbs Free Energy = 0.648945
Sum of electronic and thermal Free Energies = -3267.803658
SMD(DCE)-B3LYP-D3/6-311++G(d,p)/SDD
Single point energy = -3270.1630063
Corrected quasi-harmonic Free Energy = -3269.521654
N_{imag} = 0

Rh	-0.253569	-0.955406	-0.116434
P	2.056235	-0.745077	-0.278718
P	-0.744530	1.540311	0.152324
C	2.952083	-0.707295	1.337802
C	2.637009	0.770830	-1.156486
C	2.932155	-2.061925	-1.229085
C	0.614032	2.724223	0.640948
C	-1.635693	2.484956	-1.157674
C	-1.921252	1.645840	1.590805
C	2.556039	0.221181	2.318980
O	1.466352	1.035874	2.062250
C	1.608427	2.312763	1.546045
C	3.958275	-1.622745	1.682145
C	4.556809	-1.596895	2.944206
C	4.150041	-0.660849	3.895788
C	3.135637	0.246576	3.585537
C	0.753505	4.009149	0.091875
C	1.805579	4.851568	0.457137
C	2.750820	4.424486	1.389464
C	2.654817	3.146143	1.937027
C	-1.628673	1.968492	-2.460768
C	-2.272835	2.652370	-3.496035
C	-2.937704	3.852730	-3.238970
C	-2.962448	4.369088	-1.938860
C	-2.316827	3.690081	-0.905253
C	-3.310342	1.673203	1.373264
C	-4.206069	1.631147	2.443790
C	-3.735020	1.565487	3.756713
C	-2.357597	1.542543	3.986671
C	-1.459617	1.578263	2.917281
C	3.765154	1.494790	-0.745749
C	4.182616	2.616212	-1.466769
C	3.482209	3.021689	-2.604748
C	2.364224	2.296141	-3.024921
C	1.943584	1.176021	-2.307879
C	4.282598	-1.897157	-1.585316
C	4.952937	-2.894351	-2.293866
C	4.280549	-4.063014	-2.665097
C	2.935049	-4.224534	-2.330123
C	2.262492	-3.228271	-1.617850
H	4.278272	-2.368055	0.963412
H	5.335657	-2.315592	3.181632
H	0.041262	4.359683	-0.644563
H	1.881713	5.838205	0.009387

H	3.569187	5.075229	1.684592
H	4.313248	1.199151	0.142904
H	5.049806	3.177289	-1.129237
H	3.803976	3.899793	-3.158379
H	1.810598	2.604140	-3.907985
H	1.081121	0.609591	-2.640776
H	4.815019	-0.992438	-1.308198
H	5.997484	-2.755670	-2.559676
H	4.802119	-4.837921	-3.220748
H	2.400732	-5.122353	-2.629419
H	1.211394	-3.335608	-1.394081
H	4.607003	-0.642566	4.880920
H	-4.432029	1.534388	4.590065
H	-5.274645	1.651558	2.244943
H	-3.701570	1.723450	0.365251
H	-0.398935	1.536908	3.127326
H	-1.974122	1.494638	5.002816
H	-3.441304	4.381923	-4.043882
H	-3.484824	5.299028	-1.729623
H	-2.345023	4.096044	0.101759
H	-1.146341	1.016984	-2.659874
H	-2.260876	2.238220	-4.500913
H	2.775812	0.965994	4.314188
H	3.393839	2.786832	2.644570
Cl	-0.419185	-1.443070	-2.710764
C	-4.258821	-2.440255	0.039339
C	-4.462359	-1.217052	-0.637966
C	-5.743968	-0.840995	-1.079883
C	-6.778849	-1.724947	-0.825066
C	-6.559219	-2.955228	-0.148885
C	-5.304180	-3.340239	0.297399
H	-5.899446	0.100726	-1.596331
H	-7.786186	-1.478916	-1.147587
H	-7.405246	-3.614152	0.024032
H	-5.137434	-4.278590	0.815430
N	-3.259440	-0.563278	-0.734867
N	-2.364677	-1.305742	-0.164710
N	-2.927551	-2.434059	0.316335
H	-2.313716	-3.147890	0.707797
C	-0.075666	-2.694286	1.005959
C	-0.246973	-1.602804	1.896520
H	0.580628	-1.261570	2.512416
H	-1.222971	-1.421817	2.352565
C	-0.017420	-4.024066	0.951815
H	0.083662	-4.526372	-0.010353
C	-0.060241	-4.926682	2.162819
H	-0.876664	-5.658544	2.084713
H	0.870888	-5.503559	2.257239
H	-0.199253	-4.350944	3.084338

TS13-15-p

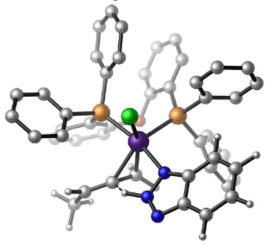

SMD(DCE)-B3LYP-D3/6-31G(d)/LanL2DZ
Thermal correction to Gibbs Free Energy = 0.645790
Sum of electronic and thermal Free Energies = -3267.775593
SMD(DCE)-B3LYP-D3/6-311++G(d,p)/SDD

Single point energy = -3270.1322745
Corrected quasi-harmonic Free Energy = -3269.494619
N_{imag} = 1, ν = -1504.1931i cm^{-1}

Rh	-0.311324	-0.953859	0.083986
P	1.944818	-0.522776	-0.327207
P	-1.000271	1.331803	0.144079
C	2.952052	-0.131128	1.174990
C	2.382568	0.804403	-1.532913
C	2.808819	-1.966445	-1.093057
C	0.258538	2.678542	0.059588
C	-2.227645	1.886647	-1.111166
C	-1.862509	1.700844	1.741184
C	2.552482	0.892937	2.056419
O	1.370330	1.584981	1.835590
C	1.343268	2.646946	0.947701
C	4.095237	-0.868785	1.523060
C	4.811247	-0.592699	2.689655
C	4.391269	0.425630	3.545813
C	3.252311	1.166791	3.229525
C	0.180310	3.752630	-0.838548
C	1.146951	4.760110	-0.833926
C	2.199825	4.717089	0.082766
C	2.301528	3.656435	0.983144
C	-2.107140	1.412399	-2.426024
C	-3.012722	1.821166	-3.406954
C	-4.049658	2.699761	-3.083467
C	-4.173402	3.177697	-1.775405
C	-3.265316	2.778705	-0.793065
C	-2.910325	0.858065	2.147739
C	-3.611478	1.108410	3.327087
C	-3.272833	2.203586	4.126679
C	-2.235338	3.048980	3.730427
C	-1.537274	2.804102	2.544407
C	3.484850	1.649954	-1.343697
C	3.835550	2.578795	-2.326246
C	3.094742	2.669093	-3.506163
C	1.999750	1.823067	-3.701998
C	1.645334	0.894057	-2.723921
C	4.091155	-1.796961	-1.645032
C	4.762497	-2.876263	-2.220485
C	4.156018	-4.134659	-2.267143
C	2.874699	-4.305430	-1.739685
C	2.202643	-3.228274	-1.157469
H	4.428474	-1.677685	0.884112
H	5.690795	-1.182917	2.929285
H	-0.637191	3.802329	-1.549010
H	1.072331	5.578611	-1.543649
H	2.947899	5.504668	0.095604
H	4.067772	1.595785	-0.430429
H	4.685864	3.235173	-2.162113
H	3.368307	3.394893	-4.267301
H	1.417666	1.883683	-4.617742
H	0.809576	0.222960	-2.886946
H	4.572043	-0.824086	-1.625124
H	5.755317	-2.730347	-2.637358
H	4.676275	-4.973920	-2.721064
H	2.388096	-5.275864	-1.787082
H	1.199672	-3.360727	-0.776115
H	4.938958	0.640154	4.458824
H	-3.814503	2.396753	5.048726
H	-4.419462	0.443585	3.620572

H	-3.180857	0.000665	1.545829
H	-0.747307	3.486444	2.252547
H	-1.966060	3.907226	4.340409
H	-4.760913	3.008375	-3.844956
H	-4.978094	3.860986	-1.517413
H	-3.370938	3.157756	0.218203
H	-1.336726	0.690986	-2.669790
H	-2.914914	1.439218	-4.419544
H	2.886279	1.953026	3.881938
H	3.118319	3.597510	1.694930
Cl	-0.590725	-1.788946	-2.400190
C	-4.083984	-3.183296	0.167022
C	-3.552348	-1.903049	-0.154400
C	-4.344714	-0.924914	-0.791182
C	-5.650732	-1.269948	-1.083382
C	-6.185615	-2.550438	-0.762812
C	-5.420555	-3.517782	-0.140029
H	-3.944496	0.047393	-1.042010
H	-6.290991	-0.543390	-1.576284
H	-7.219730	-2.764876	-1.017920
H	-5.818870	-4.497060	0.107296
N	-2.263423	-1.902315	0.286281
N	-2.070575	-3.119967	0.809752
N	-3.106032	-3.919303	0.773583
H	-0.847943	-3.126404	1.168834
C	0.303325	-2.453558	1.681065
C	-0.120040	-1.176730	2.179044
H	0.590710	-0.502050	2.647312
H	-1.120471	-1.098004	2.605478
C	1.313169	-3.275976	2.046550
H	1.584029	-4.104322	1.391398
C	2.072336	-3.205551	3.338091
H	1.852076	-4.077993	3.970580
H	3.153316	-3.227493	3.143157
H	1.833892	-2.299188	3.902857

TS14-16-p

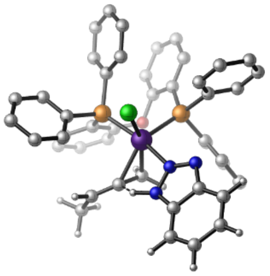

SMD(DCE)-B3LYP-D3/6-31G(d)/LanL2DZ
Thermal correction to Gibbs Free Energy = 0.646417
Sum of electronic and thermal Free Energies = -3267.777031
SMD(DCE)-B3LYP-D3/6-311++G(d,p)/SDD
Single point energy = -3270.1335674
Corrected quasi-harmonic Free Energy = -3269.495512
N_{imag} = 1, ν = -1515.9423i cm^{-1}

Rh	-0.493404	-0.702009	-0.095103
P	1.831872	-0.876029	-0.302485
P	-0.601732	1.669652	0.123163
C	2.770353	-0.861438	1.290620
C	2.689563	0.371288	-1.356658
C	2.358729	-2.455235	-1.108625
C	0.951649	2.659794	0.256664
C	-1.563206	2.587315	-1.149507

C	-1.520147	2.094149	1.675047
C	2.568763	0.181411	2.214746
O	1.625000	1.162527	1.953517
C	1.939065	2.258105	1.168655
C	3.640768	-1.896520	1.667979
C	4.287803	-1.884560	2.905669
C	4.069427	-0.837684	3.801978
C	3.199689	0.197443	3.456513
C	1.222685	3.780932	-0.542009
C	2.426643	4.476344	-0.416311
C	3.379329	4.066025	0.518786
C	3.136816	2.951505	1.321170
C	-1.676991	2.058866	-2.441894
C	-2.366131	2.766587	-3.430750
C	-2.948393	4.000769	-3.136452
C	-2.843168	4.530390	-1.845749
C	-2.155665	3.828646	-0.855756
C	-2.890919	1.784031	1.730225
C	-3.631070	2.038143	2.884452
C	-3.017220	2.607970	4.004044
C	-1.660842	2.931550	3.952578
C	-0.915715	2.679144	2.796699
C	3.997135	0.799149	-1.082632
C	4.649399	1.672796	-1.955358
C	4.003751	2.127961	-3.106845
C	2.699461	1.708538	-3.381247
C	2.043616	0.835446	-2.513005
C	3.680156	-2.604339	-1.565454
C	4.096166	-3.798652	-2.155373
C	3.195353	-4.855944	-2.308687
C	1.876495	-4.708667	-1.874818
C	1.458380	-3.515690	-1.281081
H	3.811127	-2.728620	0.995484
H	4.954598	-2.700605	3.168057
H	0.495597	4.109177	-1.275731
H	2.618098	5.336171	-1.051317
H	4.315556	4.607144	0.621773
H	4.507574	0.460239	-0.186687
H	5.659949	2.001448	-1.728041
H	4.512078	2.810411	-3.782789
H	2.186365	2.062577	-4.271438
H	1.036202	0.502614	-2.735177
H	4.391731	-1.791880	-1.460426
H	5.122247	-3.898310	-2.498934
H	3.518008	-5.784411	-2.772340
H	1.163802	-5.518589	-2.005452
H	0.428001	-3.398925	-0.976056
H	4.564178	-0.827272	4.768725
H	-3.593372	2.804849	4.904219
H	-4.689656	1.792574	2.905602
H	-3.382667	1.350263	0.867118
H	0.131326	2.953826	2.783803
H	-1.174428	3.387418	4.811020
H	-3.487159	4.547598	-3.905914
H	-3.297844	5.488803	-1.609423
H	-2.077084	4.247787	0.142840
H	-1.254191	1.085448	-2.667666
H	-2.453365	2.344628	-4.428504
H	2.990466	1.016024	4.137745
H	3.868244	2.607816	2.044979
Cl	-0.706854	-1.345403	-2.645539
C	-4.230407	-2.439539	0.086343

C	-4.688786	-1.241754	-0.520827
C	-6.038921	-1.089207	-0.892911
C	-6.885852	-2.154686	-0.640797
C	-6.418506	-3.352995	-0.033216
C	-5.095250	-3.520556	0.339528
H	-6.388815	-0.172269	-1.357278
H	-7.935235	-2.081249	-0.912004
H	-7.125397	-4.159422	0.141548
H	-4.739287	-4.435124	0.803008
N	-3.629412	-0.379882	-0.634843
N	-2.601104	-1.003982	-0.130552
N	-2.904193	-2.238556	0.305227
H	-1.806946	-2.651156	0.768027
C	-0.538405	-2.407589	1.428864
C	-0.589915	-1.086644	1.986487
H	0.251439	-0.689054	2.547468
H	-1.550784	-0.713829	2.344452
C	0.161036	-3.505232	1.797257
H	0.226520	-4.347157	1.106950
C	0.824020	-3.717153	3.126480
H	0.324094	-4.515459	3.694507
H	1.863410	-4.043354	2.981876
H	0.821775	-2.806788	3.733709

INT15-p

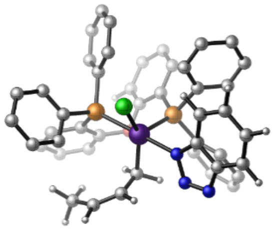

SMD(DCE)-B3LYP-D3/6-31G(d)/LanL2DZ
Thermal correction to Gibbs Free Energy = 0.654362
Sum of electronic and thermal Free Energies = -3267.824434
SMD(DCE)-B3LYP-D3/6-311++G(d,p)/SDD
Single point energy = -3270.1874295
Corrected quasi-harmonic Free Energy = -3269.542087
N_{imag} = 0

Rh	-0.263355	-0.997379	0.196424
P	2.010360	-0.436562	-0.364725
P	-1.111409	1.149408	0.247802
C	3.114570	0.247432	0.956707
C	2.225333	0.724496	-1.779112
C	2.947664	-1.926742	-0.908916
C	0.009905	2.596961	0.015326
C	-2.278364	1.374483	-1.154261
C	-2.073992	1.536723	1.764033
C	2.672664	1.234055	1.855223
O	1.388898	1.751481	1.744318
C	1.158271	2.740523	0.803525
C	4.419645	-0.243026	1.135783
C	5.227700	0.207294	2.180176
C	4.743244	1.153528	3.084600
C	3.458146	1.670423	2.919695
C	-0.262767	3.599948	-0.931158
C	0.580628	4.701814	-1.068554
C	1.706812	4.828909	-0.252535
C	1.998486	3.846986	0.692230
C	-1.896636	0.890448	-2.413804
C	-2.681743	1.142299	-3.538322

C	-3.866770	1.870776	-3.414997
C	-4.253626	2.358191	-2.164041
C	-3.460681	2.123799	-1.038946
C	-3.346866	0.964707	1.933281
C	-4.074507	1.192419	3.100418
C	-3.539484	1.983881	4.119289
C	-2.275133	2.554878	3.959416
C	-1.544277	2.334937	2.790455
C	3.083612	1.830583	-1.713803
C	3.223851	2.679224	-2.814583
C	2.523865	2.422834	-3.994770
C	1.691902	1.302528	-4.076562
C	1.545406	0.455079	-2.978807
C	3.885596	-1.861025	-1.949803
C	4.622838	-2.994131	-2.303826
C	4.433678	-4.197907	-1.622115
C	3.500513	-4.267354	-0.583506
C	2.758263	-3.140364	-0.231756
H	4.806220	-0.998984	0.462791
H	6.230165	-0.194786	2.292280
H	-1.133581	3.521909	-1.569881
H	0.353375	5.459888	-1.811758
H	2.361988	5.689561	-0.350817
H	3.638531	2.042102	-0.807240
H	3.880911	3.541607	-2.743947
H	2.631494	3.086922	-4.848125
H	1.155457	1.084266	-4.996066
H	0.920183	-0.428203	-3.054014
H	4.049027	-0.931643	-2.484768
H	5.344349	-2.931675	-3.114029
H	5.007740	-5.077862	-1.899675
H	3.341916	-5.201339	-0.051150
H	2.026166	-3.207711	0.564544
H	5.360193	1.493584	3.911110
H	-4.104560	2.155375	5.031380
H	-5.055535	0.739760	3.211631
H	-3.783954	0.356356	1.151740
H	-0.565535	2.786960	2.695653
H	-1.851813	3.175855	4.744067
H	-4.485501	2.059407	-4.288034
H	-5.171627	2.929868	-2.060195
H	-3.764725	2.535084	-0.083371
H	-0.997391	0.297219	-2.511748
H	-2.371814	0.755262	-4.505015
H	3.050240	2.412140	3.598874
H	2.872885	3.923177	1.329364
Cl	-0.232080	-2.454277	-1.980741
C	-4.398899	-2.318288	0.572565
C	-3.338966	-1.909633	-0.273615
C	-3.537066	-1.807440	-1.664987
C	-4.802497	-2.090389	-2.153135
C	-5.868392	-2.486017	-1.301719
C	-5.680027	-2.612090	0.063808
H	-2.726086	-1.537408	-2.323193
H	-4.984388	-2.012821	-3.222231
H	-6.841588	-2.699632	-1.736285
H	-6.481049	-2.924476	0.728196
N	-2.266837	-1.690743	0.560178
N	-2.669621	-1.968226	1.818261
N	-3.926568	-2.347540	1.859170
H	-0.602015	-3.024959	2.422196
C	0.262832	-2.380623	2.561170

C	0.028763	-0.956517	2.303154
H	0.843030	-0.295264	2.575648
H	-0.920332	-0.608388	2.703807
C	1.410075	-2.968998	2.976487
H	1.389024	-4.051544	3.109439
C	2.714554	-2.320240	3.317503
H	3.037464	-2.629210	4.321375
H	3.499727	-2.650717	2.623741
H	2.674737	-1.230136	3.290026

INT16-p

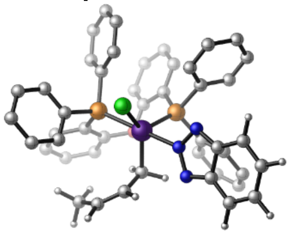

SMD(DCE)-B3LYP-D3/6-31G(d)/LanL2DZ
Thermal correction to Gibbs Free Energy = 0.652199
Sum of electronic and thermal Free Energies = -3267.827210
SMD(DCE)-B3LYP-D3/6-311++G(d,p)/SDD
Single point energy = -3270.1872618
Corrected quasi-harmonic Free Energy = -3269.543323
N_{imag} = 0

Rh	-0.293484	-0.892868	-0.252929
P	2.072152	-0.493461	-0.386929
P	-0.930740	1.246850	0.338952
C	3.063630	-0.316787	1.168604
C	2.511064	0.975960	-1.401795
C	2.955213	-1.871722	-1.230712
C	0.349097	2.547262	0.608236
C	-1.947124	1.980105	-1.004502
C	-2.023058	1.306654	1.815614
C	2.600915	0.431974	2.264789
O	1.396546	1.117973	2.180963
C	1.394230	2.332699	1.514804
C	4.290548	-0.985451	1.324237
C	4.998410	-0.935580	2.525624
C	4.487467	-0.224287	3.612119
C	3.282414	0.465842	3.479046
C	0.327588	3.770154	-0.084225
C	1.300592	4.741659	0.148562
C	2.312221	4.511143	1.083054
C	2.363161	3.300675	1.771591
C	-1.740220	1.592626	-2.334818
C	-2.442698	2.217375	-3.365785
C	-3.364599	3.226185	-3.077564
C	-3.582253	3.611863	-1.751879
C	-2.875954	2.995328	-0.718150
C	-3.350296	0.862827	1.678241
C	-4.194966	0.811596	2.785946
C	-3.727307	1.198671	4.044673
C	-2.412049	1.645583	4.186833
C	-1.560815	1.698996	3.080736
C	3.532914	1.862858	-1.036970
C	3.839790	2.953427	-1.854244
C	3.138167	3.161317	-3.043515
C	2.131854	2.267597	-3.421308
C	1.819949	1.178724	-2.607634
C	3.958921	-1.628852	-2.178572

C	4.651800	-2.696625	-2.756182
C	4.353488	-4.009952	-2.388548
C	3.353390	-4.255629	-1.442399
C	2.654021	-3.193823	-0.870696
H	4.692829	-1.567499	0.503749
H	5.941620	-1.466552	2.612775
H	-0.445545	3.965134	-0.816511
H	1.266516	5.675591	-0.403951
H	3.068340	5.267668	1.271652
H	4.083541	1.714779	-0.114354
H	4.625085	3.642031	-1.554881
H	3.376020	4.013792	-3.674020
H	1.586227	2.416613	-4.349093
H	1.056447	0.472970	-2.914839
H	4.206682	-0.613486	-2.469116
H	5.424875	-2.497246	-3.493398
H	4.893684	-4.838348	-2.838936
H	3.109634	-5.275127	-1.155785
H	1.867626	-3.392449	-0.151546
H	5.023836	-0.198095	4.556010
H	-4.385106	1.154983	4.908455
H	-5.216463	0.463426	2.660938
H	-3.732514	0.572535	0.707038
H	-0.543967	2.043401	3.219114
H	-2.042025	1.955641	5.160276
H	-3.915331	3.707535	-3.881288
H	-4.299885	4.393820	-1.519403
H	-3.045539	3.311660	0.305898
H	-1.063235	0.779388	-2.564067
H	-2.278097	1.902480	-4.392530
H	2.859711	1.038233	4.298459
H	3.149863	3.096098	2.489531
Cl	-0.130837	-1.904984	-2.601993
C	-4.254533	-2.212611	0.161481
C	-4.357762	-1.483395	-1.054449
C	-5.588001	-1.375984	-1.740350
C	-6.681778	-2.007516	-1.176192
C	-6.578855	-2.738285	0.043916
C	-5.380253	-2.853549	0.725272
H	-5.664491	-0.815946	-2.668305
H	-7.648696	-1.948971	-1.669429
H	-7.470753	-3.213886	0.443964
H	-5.302567	-3.408301	1.656284
N	-3.130547	-0.970515	-1.334268
N	-2.354433	-1.376789	-0.333584
N	-2.969220	-2.118915	0.589327
H	-1.011618	-3.443695	1.256825
C	-0.162812	-2.928698	1.701718
C	-0.308885	-1.471503	1.798048
H	0.467305	-0.958364	2.355561
H	-1.301021	-1.177562	2.134739
C	0.874106	-3.690922	2.121639
H	0.791647	-4.766467	1.958541
C	2.123199	-3.256429	2.820901
H	2.246458	-3.818354	3.757248
H	3.004302	-3.482812	2.205339
H	2.137892	-2.190921	3.055183

INT19*

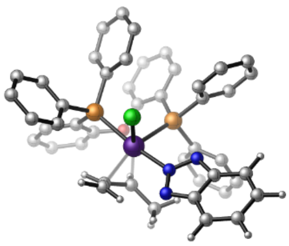

SMD(DCE)-B3LYP-D3/6-31G(d)/LanL2DZ
Thermal correction to Gibbs Free Energy = 0.656552
Sum of electronic and thermal Free Energies = -3267.812433
SMD(DCE)-B3LYP-D3/6-311++G(d,p)/SDD
Single point energy = -3270.1773595
Corrected quasi-harmonic Free Energy = -3269.530328
N_{imag} = 0

Rh	0.221181	1.137795	0.375207
P	-2.130252	0.790285	-0.101722
P	0.825602	-1.290932	-0.075613
C	-3.123755	0.246596	1.362569
C	-2.505834	-0.438901	-1.423140
C	-3.075158	2.253845	-0.722935
C	-0.531725	-2.574688	-0.103245
C	1.768726	-1.706733	-1.603796
C	1.908637	-1.969407	1.278310
C	-2.673140	-0.838382	2.136412
O	-1.508835	-1.492993	1.766316
C	-1.555994	-2.538321	0.854799
C	-4.246514	0.949325	1.830824
C	-4.890298	0.576490	3.012895
C	-4.416367	-0.500267	3.763309
C	-3.296238	-1.208474	3.325239
C	-0.587020	-3.615034	-1.045726
C	-1.583823	-4.591127	-0.996636
C	-2.556477	-4.550708	0.002544
C	-2.547437	-3.513529	0.933025
C	1.788921	-0.803736	-2.673785
C	2.476098	-1.115362	-3.848653
C	3.161065	-2.327231	-3.964678
C	3.159307	-3.227640	-2.895954
C	2.466885	-2.921149	-1.722907
C	3.309121	-1.910403	1.169903
C	4.127310	-2.347469	2.213370
C	3.566282	-2.852594	3.387810
C	2.176979	-2.927712	3.502462
C	1.356272	-2.494231	2.459288
C	-3.595155	-1.317238	-1.328702
C	-3.893861	-2.180412	-2.384789
C	-3.112267	-2.174832	-3.542265
C	-2.029945	-1.297471	-3.642075
C	-1.729495	-0.430539	-2.591511
C	-4.361904	2.061640	-1.259815
C	-5.112501	3.147517	-1.709907
C	-4.586782	4.440953	-1.642355
C	-3.304643	4.637986	-1.128269
C	-2.552126	3.551641	-0.674555
H	-4.620451	1.803538	1.279238
H	-5.756828	1.138496	3.348311
H	0.147622	-3.664521	-1.838880
H	-1.596221	-5.379078	-1.743938
H	-3.329022	-5.312726	0.053051
H	-4.204038	-1.340836	-0.431208
H	-4.732756	-2.864936	-2.294765

H	-3.343350	-2.853663	-4.358667
H	-1.411069	-1.287477	-4.535133
H	-0.898794	0.258158	-2.678678
H	-4.786510	1.065368	-1.325890
H	-6.105244	2.979924	-2.118632
H	-5.169924	5.286198	-1.997841
H	-2.877539	5.636314	-1.088770
H	-1.543494	3.711067	-0.325854
H	-4.908161	-0.785551	4.688545
H	4.203748	-3.187492	4.201719
H	5.206826	-2.287891	2.102775
H	3.763581	-1.512056	0.273953
H	0.285172	-2.559985	2.590706
H	1.722933	-3.326269	4.405940
H	3.701190	-2.565552	-4.877251
H	3.695761	-4.169722	-2.971899
H	2.474113	-3.630649	-0.901232
H	1.302311	0.158563	-2.575127
H	2.485382	-0.400674	-4.667406
H	-2.891807	-2.040980	3.891983
H	-3.308681	-3.445540	1.702653
Cl	0.301182	2.317039	-1.865921
C	4.141476	2.646887	0.296720
C	4.304237	1.376430	-0.317462
C	5.547830	0.968524	-0.844840
C	6.599746	1.861807	-0.738083
C	6.438529	3.137044	-0.122865
C	5.223345	3.548576	0.397228
H	5.664274	-0.001886	-1.319709
H	7.576059	1.591363	-1.132144
H	7.297180	3.801295	-0.066341
H	5.102349	4.522180	0.864235
N	3.105163	0.740623	-0.257017
N	2.290511	1.585982	0.353450
N	2.847774	2.744225	0.704288
H	-1.509986	1.806513	2.530694
C	-0.441359	1.922333	2.373582
C	0.006347	3.055614	1.686544
H	1.048722	3.351628	1.664677
H	-0.716866	3.818535	1.422620
C	0.399850	0.803951	2.627075
H	-0.113101	-0.103180	2.928447
C	1.805151	0.914532	3.162226
H	2.470098	0.162721	2.732722
H	2.243903	1.899130	2.999456
H	1.761902	0.728489	4.245317

INT20*

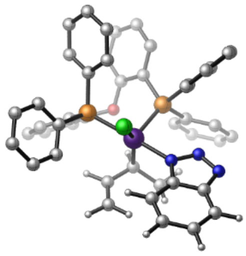

SMD(DCE)-B3LYP-D3/6-31G(d)/LanL2DZ
Thermal correction to Gibbs Free Energy = 0.655900
Sum of electronic and thermal Free Energies = -3267.812557
SMD(DCE)-B3LYP-D3/6-311++G(d,p)/SDD
Single point energy = -3270.1775106
Corrected quasi-harmonic Free Energy = -3269.530985

N_{imag} = 0

Rh	0.529103	0.781718	0.185031
P	-1.888380	0.799780	-0.067463
P	0.795661	-1.546319	-0.054661
C	-2.902812	0.399764	1.426944
C	-2.569882	-0.256503	-1.414797
C	-2.527189	2.474733	-0.520953
C	-0.755987	-2.569122	-0.159031
C	1.685743	-1.971253	-1.610303
C	1.749744	-2.451190	1.256368
C	-2.587223	-0.717839	2.218084
O	-1.544130	-1.551654	1.843179
C	-1.751666	-2.468552	0.822810
C	-3.938348	1.236273	1.876046
C	-4.612129	0.972875	3.070317
C	-4.256750	-0.130008	3.849087
C	-3.237183	-0.981264	3.420483
C	-0.986946	-3.473793	-1.208580
C	-2.132674	-4.268340	-1.243047
C	-3.079556	-4.178366	-0.222090
C	-2.891217	-3.269203	0.816128
C	1.898053	-1.000694	-2.597266
C	2.550835	-1.336141	-3.785850
C	3.001455	-2.639505	-4.000137
C	2.797473	-3.613198	-3.017924
C	2.145125	-3.283776	-1.830069
C	3.123899	-2.703204	1.092215
C	3.850311	-3.361529	2.085052
C	3.225712	-3.773276	3.263758
C	1.862093	-3.530451	3.436215
C	1.128797	-2.878982	2.443187
C	-3.848589	-0.825527	-1.320948
C	-4.379950	-1.543286	-2.394822
C	-3.641616	-1.699933	-3.569695
C	-2.366004	-1.138050	-3.666257
C	-1.830894	-0.419964	-2.596831
C	-3.591058	2.626536	-1.423250
C	-4.092304	3.897269	-1.717790
C	-3.540313	5.029740	-1.117056
C	-2.480057	4.885342	-0.218065
C	-1.975955	3.618701	0.074718
H	-4.214268	2.107609	1.293496
H	-5.408936	1.636412	3.393092
H	-0.276925	-3.552720	-2.020942
H	-2.282937	-4.951568	-2.073323
H	-3.970211	-4.799712	-0.238939
H	-4.433218	-0.712024	-0.413586
H	-5.369409	-1.983766	-2.307210
H	-4.056318	-2.261166	-4.402721
H	-1.780204	-1.260976	-4.573216
H	-0.845035	0.020160	-2.681976
H	-4.035250	1.762104	-1.903169
H	-4.913866	3.996662	-2.422077
H	-3.929784	6.017145	-1.349843
H	-2.036802	5.759752	0.251056
H	-1.143033	3.526769	0.757146
H	-4.770378	-0.331586	4.784562
H	3.795300	-4.279418	4.038525
H	4.910411	-3.544982	1.933168
H	3.634439	-2.373034	0.199388
H	0.078984	-2.695090	2.619595

H	1.359630	-3.847040	4.346225
H	3.513383	-2.896501	-4.923652
H	3.147399	-4.630129	-3.172534
H	1.994906	-4.050652	-1.077005
H	1.568915	0.019661	-2.440498
H	2.709979	-0.569766	-4.539549
H	-2.937651	-1.848948	3.999690
H	-3.625355	-3.160235	1.607472
Cl	0.451587	2.179330	-2.022025
C	3.264523	2.267578	-0.036372
C	4.632264	1.905152	-0.086368
C	5.641530	2.883222	-0.190275
C	5.244799	4.209551	-0.237211
C	3.871077	4.569870	-0.190819
C	2.867404	3.618495	-0.094698
H	6.689081	2.597265	-0.230664
H	5.992920	4.994113	-0.316581
H	3.602168	5.622026	-0.241418
H	1.820627	3.896842	-0.088577
N	4.711628	0.536745	-0.023356
N	3.478383	0.086432	0.068037
N	2.583599	1.082232	0.069306
H	-1.023615	1.940599	2.600107
C	0.059476	1.921225	2.493745
C	0.716998	3.104571	2.444854
H	1.795480	3.164781	2.355386
H	0.182101	4.047944	2.514550
C	0.689991	0.606842	2.341314
H	0.040179	-0.208693	2.642860
C	2.090179	0.441214	2.905590
H	2.581217	-0.453756	2.528025
H	2.740925	1.291253	2.692768
H	2.010008	0.342196	3.998436

TS19-21*

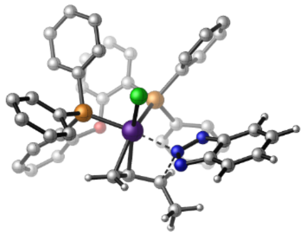

SMD(DCE)-B3LYP-D3/6-31G(d)/LanL2DZ
Thermal correction to Gibbs Free Energy = 0.651406
Sum of electronic and thermal Free Energies = -3267.773357
SMD(DCE)-B3LYP-D3/6-311++G(d,p)/SDD
Single point energy = -3270.1297682
Corrected quasi-harmonic Free Energy = -3269.486709
N_{imag} = 1, ν = -335.0536i cm^{-1}

Rh	-0.209924	-0.994093	0.308607
P	2.046338	-0.883199	-0.131874
P	-0.737148	1.515348	-0.096673
C	3.052516	-0.426967	1.351805
C	2.603139	0.274018	-1.454726
C	2.794421	-2.483312	-0.682760
C	0.723566	2.672720	-0.153500
C	-1.741825	2.024485	-1.556859
C	-1.713893	2.245113	1.315591
C	2.715098	0.723115	2.089417
O	1.629307	1.489917	1.696665
C	1.777594	2.504838	0.760256

C	4.088099	-1.233701	1.849566
C	4.759330	-0.902213	3.028674
C	4.400162	0.241375	3.743562
C	3.367598	1.055450	3.274655
C	0.869498	3.693489	-1.105616
C	1.990417	4.525680	-1.120874
C	3.000604	4.353538	-0.174377
C	2.895701	3.336036	0.772616
C	-1.953760	1.076290	-2.567196
C	-2.715052	1.402324	-3.693825
C	-3.276076	2.674227	-3.819164
C	-3.077945	3.624332	-2.811079
C	-2.318461	3.301832	-1.686503
C	-3.116897	2.323046	1.243320
C	-3.868419	2.782292	2.326072
C	-3.238734	3.170437	3.510940
C	-1.847223	3.100234	3.596767
C	-1.092325	2.644348	2.512672
C	3.837996	0.936890	-1.383688
C	4.262152	1.749382	-2.437353
C	3.458251	1.911000	-3.567888
C	2.225620	1.257458	-3.641265
C	1.798469	0.442133	-2.592747
C	4.064460	-2.499574	-1.285491
C	4.650854	-3.705542	-1.672844
C	3.974300	-4.911254	-1.471118
C	2.705832	-4.902231	-0.887234
C	2.116456	-3.697318	-0.499103
H	4.371908	-2.133933	1.316855
H	5.557461	-1.544772	3.388721
H	0.105410	3.836390	-1.859791
H	2.070956	5.302442	-1.875750
H	3.874004	4.999580	-0.174572
H	4.467778	0.828973	-0.506282
H	5.217136	2.263047	-2.366975
H	3.787998	2.549095	-4.383489
H	1.588886	1.384785	-4.512680
H	0.844511	-0.068025	-2.656751
H	4.602797	-1.573214	-1.456985
H	5.633748	-3.699632	-2.136281
H	4.429358	-5.849877	-1.776214
H	2.164017	-5.833006	-0.741817
H	1.118115	-3.697593	-0.081907
H	4.914454	0.498352	4.665024
H	-3.825034	3.524934	4.354569
H	-4.950798	2.833392	2.239588
H	-3.629493	2.014134	0.342670
H	-0.017807	2.591137	2.620949
H	-1.339126	3.402518	4.509028
H	-3.870608	2.925505	-4.693736
H	-3.517844	4.614306	-2.898919
H	-2.180658	4.041749	-0.903499
H	-1.538322	0.078508	-2.465758
H	-2.873256	0.655513	-4.467449
H	3.054558	1.944083	3.813913
H	3.680104	3.172008	1.503670
Cl	-0.500064	-2.130408	-1.989571
C	-3.911527	-2.727994	0.105564
C	-4.279248	-1.360896	-0.115900
C	-5.530808	-1.021640	-0.691516
C	-6.368347	-2.063400	-1.021012
C	-6.002028	-3.431919	-0.798504

C	-4.791824	-3.784555	-0.244519
H	-5.802287	0.015046	-0.866770
H	-7.336739	-1.853858	-1.467309
H	-6.707467	-4.207799	-1.083588
H	-4.512614	-4.821375	-0.082740
N	-3.265768	-0.586201	0.301834
N	-2.323534	-1.452179	0.725855
N	-2.682254	-2.747355	0.646048
H	0.421720	-0.376606	2.815693
C	-0.236607	-1.152847	2.434615
C	0.248627	-2.397999	1.929170
H	-0.411864	-3.259770	1.885572
H	1.300338	-2.636446	2.063054
C	-1.649427	-0.939325	2.636125
H	-1.970569	0.098605	2.639579
C	-2.533416	-1.863648	3.401207
H	-3.587959	-1.711458	3.154165
H	-2.261060	-2.913031	3.286437
H	-2.397005	-1.582480	4.458313

TS20-22*

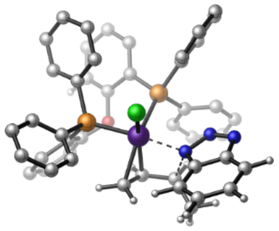

SMD(DCE)-B3LYP-D3/6-31G(d)/LanL2DZ
Thermal correction to Gibbs Free Energy = 0.653629
Sum of electronic and thermal Free Energies = -3267.774570
SMD(DCE)-B3LYP-D3/6-311++G(d,p)/SDD
Single point energy = -3270.1348294
Corrected quasi-harmonic Free Energy = -3269.490258
N_{imag} = 1, ν = -299.9621i cm^{-1}

Rh	0.440417	0.874227	0.214185
P	-1.859097	0.966549	-0.075885
P	0.661424	-1.670109	-0.125047
C	-2.825001	0.655635	1.471327
C	-2.592504	-0.163627	-1.333375
C	-2.483500	2.618050	-0.629081
C	-0.916892	-2.666217	-0.051249
C	1.485327	-2.306633	-1.645063
C	1.646356	-2.485770	1.236285
C	-2.547369	-0.483161	2.249976
O	-1.564817	-1.371194	1.839378
C	-1.871407	-2.388209	0.941740
C	-3.761543	1.571566	1.977422
C	-4.391550	1.358434	3.205626
C	-4.087764	0.226056	3.962712
C	-3.156391	-0.696959	3.484420
C	-1.245122	-3.671060	-0.975842
C	-2.437929	-4.390749	-0.883136
C	-3.338150	-4.122481	0.148064
C	-3.055563	-3.112070	1.065423
C	1.740852	-1.396581	-2.680118
C	2.354267	-1.823823	-3.861836
C	2.725214	-3.160233	-4.017936
C	2.487333	-4.073238	-2.984581
C	1.874122	-3.649808	-1.805765
C	3.002264	-2.809777	1.047665

C	3.762562	-3.353063	2.084238
C	3.192147	-3.577081	3.339587
C	1.849646	-3.255270	3.543540
C	1.084631	-2.716428	2.505252
C	-3.863185	-0.734155	-1.166796
C	-4.412338	-1.534377	-2.171239
C	-3.700165	-1.771977	-3.348980
C	-2.434209	-1.205946	-3.519292
C	-1.881825	-0.405068	-2.519514
C	-3.743874	2.733384	-1.239548
C	-4.234046	3.982765	-1.624619
C	-3.471147	5.132800	-1.409186
C	-2.212482	5.025242	-0.812873
C	-1.718983	3.777079	-0.429376
H	-3.997080	2.465924	1.412036
H	-5.112556	2.083867	3.570931
H	-0.570725	-3.890304	-1.794212
H	-2.658460	-5.157738	-1.619827
H	-4.263965	-4.684192	0.234347
H	-4.424277	-0.564767	-0.253043
H	-5.393198	-1.979006	-2.025971
H	-4.127254	-2.400613	-4.125809
H	-1.868678	-1.390913	-4.428713
H	-0.902836	0.037955	-2.658438
H	-4.349765	1.851536	-1.419926
H	-5.210272	4.053325	-2.096781
H	-3.850928	6.104787	-1.712666
H	-1.605274	5.912733	-0.655392
H	-0.728129	3.697486	-0.001786
H	-4.568066	0.060124	4.922658
H	3.786595	-3.997162	4.146650
H	4.806619	-3.597300	1.906115
H	3.475998	-2.631131	0.091669
H	0.051609	-2.467224	2.703971
H	1.387023	-3.422843	4.512929
H	3.204102	-3.491100	-4.935875
H	2.781622	-5.113608	-3.095478
H	1.701830	-4.363760	-1.005810
H	1.470975	-0.351934	-2.559085
H	2.546033	-1.106288	-4.655258
H	-2.891200	-1.582788	4.052960
H	-3.752975	-2.869280	1.859773
Cl	0.637121	1.979167	-2.093373
C	3.299529	2.242876	0.085034
C	4.555438	1.795561	-0.366163
C	5.549958	2.689545	-0.789971
C	5.245985	4.043558	-0.742411
C	3.979599	4.493521	-0.297271
C	2.988990	3.609948	0.114849
H	6.510941	2.328066	-1.143722
H	5.984491	4.773500	-1.061911
H	3.772556	5.560307	-0.291242
H	2.012481	3.963924	0.418768
N	4.559237	0.410611	-0.312114
N	3.409455	0.026721	0.136485
N	2.589414	1.099956	0.407511
H	-0.001457	0.258412	2.740282
C	0.656914	1.024991	2.341275
C	0.181876	2.294679	1.897588
H	0.855947	3.145283	1.875938
H	-0.851303	2.560012	2.102503
C	2.068397	0.757241	2.443522

H	2.347023	-0.292691	2.474399
C	3.058743	1.683795	3.056541
H	4.080992	1.434239	2.758319
H	2.853695	2.737030	2.856083
H	2.981653	1.524245	4.144213

INT21*

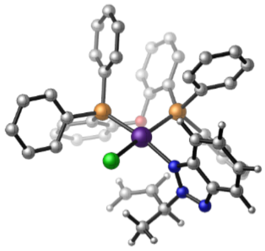

SMD(DCE)-B3LYP-D3/6-31G(d)/LanL2DZ
Thermal correction to Gibbs Free Energy = 0.655072
Sum of electronic and thermal Free Energies = -3267.829082
SMD(DCE)-B3LYP-D3/6-311++G(d,p)/SDD
Single point energy = -3270.1872574
Corrected quasi-harmonic Free Energy = -3269.540085
N_{imag} = 0

Rh	-0.209265	-0.896698	-0.327524
P	2.071003	-0.485866	-0.407409
P	-0.859049	1.237202	0.283941
C	2.981863	-0.407877	1.226260
C	2.629507	1.030164	-1.299145
C	3.050848	-1.813884	-1.248236
C	0.383821	2.539732	0.697069
C	-1.826854	2.094606	-1.042770
C	-2.034753	1.322000	1.722290
C	2.453955	0.259358	2.345994
O	1.275043	0.981927	2.243480
C	1.367347	2.232024	1.643694
C	4.212130	-1.065091	1.413625
C	4.844589	-1.105747	2.657558
C	4.258916	-0.487428	3.762621
C	3.060796	0.207292	3.600148
C	0.439619	3.801355	0.084891
C	1.436621	4.718087	0.422082
C	2.392667	4.390212	1.386110
C	2.361554	3.140811	2.004116
C	-1.527439	1.813820	-2.382487
C	-2.174790	2.488803	-3.419221
C	-3.141590	3.453054	-3.127104
C	-3.448938	3.743821	-1.794570
C	-2.792107	3.076115	-0.759068
C	-3.356943	0.879193	1.542180
C	-4.243432	0.801585	2.615650
C	-3.829280	1.176152	3.896422
C	-2.527233	1.643101	4.085215
C	-1.637598	1.718155	3.009801
C	3.673097	1.852331	-0.855552
C	4.045757	2.977508	-1.595752
C	3.393447	3.281921	-2.792703
C	2.366320	2.453024	-3.253560
C	1.986912	1.336397	-2.508982
C	3.825297	-1.578052	-2.390509
C	4.554368	-2.617523	-2.979835
C	4.529852	-3.896380	-2.423657
C	3.764335	-4.138323	-1.277301

C	3.020857	-3.109888	-0.703114
H	4.685060	-1.565359	0.577886
H	5.790749	-1.629563	2.758577
H	-0.290028	4.068206	-0.670689
H	1.466086	5.685054	-0.071440
H	3.167996	5.101998	1.655057
H	4.187026	1.627619	0.073223
H	4.844450	3.618298	-1.231699
H	3.682901	4.160287	-3.363464
H	1.855495	2.677727	-4.186074
H	1.195759	0.687104	-2.869372
H	3.871964	-0.587291	-2.828948
H	5.147086	-2.418032	-3.868887
H	5.102116	-4.701222	-2.877713
H	3.736458	-5.132267	-0.838513
H	2.418944	-3.307056	0.177768
H	4.734844	-0.528451	4.738081
H	-4.516657	1.112636	4.735688
H	-5.256417	0.446635	2.446269
H	-3.708409	0.608588	0.553722
H	-0.634456	2.080926	3.193173
H	-2.196447	1.953807	5.072852
H	-3.654096	3.974615	-3.931049
H	-4.198413	4.494672	-1.558641
H	-3.033446	3.325227	0.269244
H	-0.796385	1.045874	-2.606186
H	-1.930656	2.251363	-4.451244
H	2.585461	0.725130	4.427251
H	3.102386	2.864699	2.746629
Cl	-0.033185	-3.075308	-1.533068
C	-4.454876	-1.911455	-0.357631
C	-3.286738	-1.420366	-1.003241
C	-3.334014	-0.927693	-2.325292
C	-4.568641	-0.917383	-2.939528
C	-5.748839	-1.381655	-2.282067
C	-5.715382	-1.884596	-0.999003
H	-2.436995	-0.593780	-2.827718
H	-4.650865	-0.550583	-3.958694
H	-6.692369	-1.344604	-2.819086
H	-6.603587	-2.253838	-0.496525
N	-2.256404	-1.547416	-0.129508
N	-2.809292	-2.110949	0.951556
N	-4.109111	-2.351149	0.872856
H	-1.080204	-0.898963	2.717867
C	-0.878097	-1.948737	2.532374
C	0.308764	-2.466115	2.862347
H	0.559132	-3.512424	2.714246
H	1.085324	-1.838552	3.286190
C	-2.055812	-2.761026	2.060467
H	-2.800202	-2.773215	2.864235
C	-1.742567	-4.199053	1.635945
H	-2.661456	-4.696823	1.312291
H	-1.023396	-4.205619	0.811983
H	-1.333578	-4.754166	2.485099

INT22*

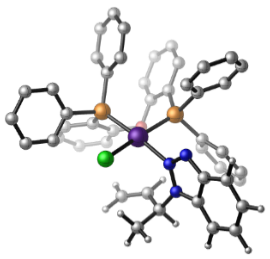

SMD(DCE)-B3LYP-D3/6-31G(d)/LanL2DZ
Thermal correction to Gibbs Free Energy = 0.655239
Sum of electronic and thermal Free Energies = -3267.831175
SMD(DCE)-B3LYP-D3/6-311++G(d,p)/SDD
Single point energy = -3270.1885512
Corrected quasi-harmonic Free Energy = -3269.541644
N_{imag} = 0

Rh	-0.172276	-0.777769	-0.480739
P	2.129439	-0.664812	-0.338681
P	-0.586124	1.385914	0.169589
C	2.861209	-0.841922	1.372771
C	2.945549	0.841898	-1.024318
C	3.022967	-2.019718	-1.229168
C	0.763322	2.464387	0.826408
C	-1.290362	2.403709	-1.204635
C	-1.917929	1.558222	1.460732
C	2.293579	-0.202266	2.490246
O	1.245057	0.692347	2.334951
C	1.575270	1.952193	1.845923
C	3.964312	-1.678335	1.623471
C	4.431251	-1.914031	2.918086
C	3.800465	-1.317150	4.009898
C	2.731619	-0.448099	3.790557
C	1.085965	3.731061	0.315394
C	2.161694	4.459757	0.823853
C	2.935893	3.934479	1.860619
C	2.644206	2.672955	2.377007
C	-1.188741	1.947253	-2.525848
C	-1.696383	2.709129	-3.581273
C	-2.320104	3.932063	-3.325642
C	-2.439366	4.390823	-2.009610
C	-1.930787	3.630685	-0.956013
C	-3.263450	1.449116	1.067634
C	-4.290972	1.447383	2.011325
C	-3.995807	1.564667	3.371909
C	-2.665193	1.695968	3.774189
C	-1.634854	1.693043	2.829906
C	4.115747	1.390745	-0.483304
C	4.709133	2.507415	-1.076769
C	4.148975	3.076473	-2.223350
C	2.989059	2.526193	-2.775706
C	2.390156	1.417005	-2.177540
C	3.878529	-1.770423	-2.309832
C	4.522805	-2.827154	-2.962994
C	4.329131	-4.140453	-2.534452
C	3.479243	-4.397317	-1.453042
C	2.821304	-3.348163	-0.814635
H	4.463771	-2.167300	0.796304
H	5.282785	-2.571575	3.067603
H	0.507888	4.148459	-0.500179
H	2.396855	5.433072	0.403421
H	3.772318	4.498980	2.262849
H	4.560574	0.956401	0.406502

H	5.608221	2.933763	-0.639636
H	4.611463	3.947004	-2.681019
H	2.544006	2.963253	-3.665655
H	1.486806	0.993056	-2.602759
H	4.052077	-0.755223	-2.649927
H	5.180652	-2.615938	-3.802090
H	4.834396	-4.960432	-3.038176
H	3.318398	-5.417818	-1.115322
H	2.153518	-3.557467	0.014075
H	4.144595	-1.509018	5.022101
H	-4.793911	1.561459	4.109536
H	-5.321318	1.353429	1.678407
H	-3.517478	1.373422	0.016976
H	-0.614124	1.783114	3.177593
H	-2.421078	1.801194	4.828219
H	-2.718387	4.523679	-4.145850
H	-2.930945	5.337940	-1.803327
H	-2.031041	3.993257	0.062943
H	-0.731442	0.981447	-2.717715
H	-1.612029	2.340403	-4.600048
H	2.232955	0.059932	4.610030
H	3.243685	2.240515	3.170808
Cl	-0.192930	-2.949180	-1.727133
C	-4.364350	-1.632348	-0.085207
C	-4.317769	-0.925240	-1.302133
C	-5.492049	-0.646500	-2.025379
C	-6.682981	-1.108147	-1.490504
C	-6.718026	-1.828565	-0.266859
C	-5.569075	-2.108168	0.457643
H	-5.451644	-0.092023	-2.957434
H	-7.615888	-0.917490	-2.012744
H	-7.676788	-2.171362	0.111389
H	-5.599837	-2.659182	1.391317
N	-3.013231	-0.588791	-1.556463
N	-2.286395	-1.048585	-0.581210
N	-3.064154	-1.682794	0.336420
H	-1.371577	-0.910199	2.371457
C	-1.325905	-1.961932	2.106152
C	-0.273969	-2.697571	2.478227
H	-0.181266	-3.755328	2.250336
H	0.554079	-2.243814	3.013672
C	-2.573988	-2.516821	1.463736
H	-3.376908	-2.416342	2.205639
C	-2.494367	-3.977760	1.013189
H	-3.448782	-4.288413	0.577054
H	-1.706150	-4.102844	0.265919
H	-2.289686	-4.621703	1.873787
